# Benzotriazole-Mediated Synthesis and Antibacterial Activity of Novel *N*-Acylcephalexins

**DOI:** 10.3390/scipharm84030484

**Published:** 2016-04-13

**Authors:** Khalid A. Agha, Nader E. Abo-Dya, Tarek S. Ibrahim, Eatedal H. Abdel-Aal, Wael A. Hegazy

**Affiliations:** 1 Department of Pharmaceutical Organic Chemistry, Faculty of Pharmacy, Zagazig University, Zagazig 44519, Egypt; aghanet2010@yahoo.com (K.A.A.); Tarekeldeeb1976@yahoo.com (T.S.I.); eatedalabdelaal@yahoo.com (E.H.A.-A.); 2 Department of Pharmaceutical Chemistry, Faculty of Pharmacy, University of Tabuk, Tabuk 71491, Saudi Arabia; 3 Department of Microbiology and Immunology, Faculty of Pharmacy, Zagazig University, Zagazig 44519, Egypt; Wmhegazy@yahoo.com

**Keywords:** acylation, antibiotics, benzotriazolides, drug research

## Abstract

Cephalexin (**1**) was acylated using *N*-acylbenzotriazoles (**3a**–**k′**) derived from various carboxylic acids including aromatic, heterocyclic and *N*-Pg-α-amino acid to afford *N*-acylcephalexins in excellent yields (82%–96%). Antibacterial screening of the novel cephalosporins revealed that all targets (**4a**–**j**) retained the antibacterial activity of cephalexin against *Staphylococcus aureus* (ATCC 6538). *N*-Nicotinylcephalexin (**4c**) and *N*-(3,4,5-trimethoxybenzoyl)cephalexin (**4g**) exhibited a broader spectrum of antibacterial activity towards standard strains of *Staphylococcus aureus* (ATCC 6538), *Paenibacillus polymyxa* (ATCC 842), and *Escherichia coli* (ATCC 10536) as well as a resistant strain of *Pseudomonas aeruginosa* (ATCC 27853).

## 1. Introduction

Effective antibiotics are needed to combat the growing bacterial resistance [1]. Cephalosporins are an important class of antimicrobials with a broad-spectrum of activity and a favourable safety profile [2]. The traditional approach to develop new antibiotics involves antimicrobial screening of natural products [3,4]. However, building new analogues of those antibiotics which are currently in use is more financially feasible because these analogues tend to have similar solubility, protein binding, and toxicity as the parent compounds [5].

Acylation reactions enabled the development of various generations of cephalosporins [6]. In addition, cephalosporins that contain nitrogen nucleophiles can be further acylated in order to modulate their antimicrobial activity, improve their solubility, and introduce fluorescent tags [7,8,9]. Such acylations have been accomplished utilizing acid chlorides, acid anhydrides, and ethyl chloroformate [7,10,11].

*N*-acylbenzotriazoles are advantageous acylating agents showing numerous merits over acid chlorides because: (i) they are usually isolated in high yields; (ii) they form crystals easily; (iii) they are stable in air; and (iv) chirality is preserved during the course of their preparation and reaction. These carboxylic acid surrogates are widely used when the corresponding acid chlorides are unstable or difficult to prepare [12].

**Figure 1:**
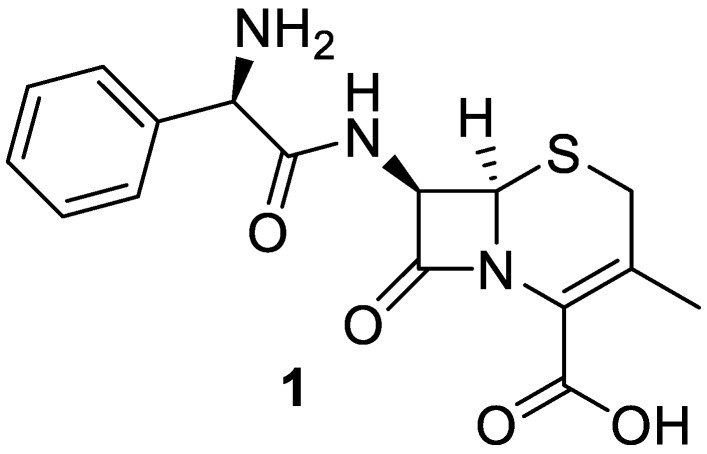
Cephalexin.

Amino acid conjugates of quinolone, metronidazole, and sulfadiazine antibiotics were recently synthesized in good yields using the benzotriazole methodology [13]. In the current work, cephalexin (Figure 1), which is a first generation cephalosporin on the World Health Organization’s list of essential medicines, was selected for further acylation with a variety of *N*-acylbenzotriazoles (**3a**–**k′**). *N*-acylcephalexins (**4a**–**k′**) were obtained in pure form in high yields by a simple work-up and their antibacterial activity was evaluated [4].

## 2. Materials and Methods

### 2.1. Chemistry

Starting materials and solvents were purchased from common commercial sources and used without further purification. Melting points were determined on the Fisher Melting Apparatus, (Pittsburgh, PA, USA). ^1^H-NMR (400 MHz) and ^13^C-NMR (100 MHz) spectra were recorded on a Bruker 400 MHz NMR Spectrometer (Bruker, Fällanden, Switzerland) in DMSO-*d*_6_. *J* values are given in Hz, using tetramethylsilane (TMS) as the internal standard at the Faculty of Science, Zagazig University. ^1^H and ^13^C NMR spectra of Compounds **7d**–**j** and **8a**–**j** can be found in Supplementary Figures S1 and S2. Elemental analyses were performed on the Carlo Erba-1106 (Thermo Fisher Scientific Inc., Waltham, MA, USA) instrument at the Regional Center for Mycology & Biotechnology, Al-Azhar University, Cairo. The reactions were followed by Thin Layer Chromatography (TLC) (silica gel, aluminum sheets 60 F254, Merck, Darmstadt, Germany). The purity of the newly synthesized compounds was assessed by TLC and elemental analysis.

*Procedure for the Synthesis of 4-methyl-2-(3,4,5trimethoxybenzamido) thiazole-5-carboxylic acid* (**2j**): Thiourea (0.152 g, 2 mmol) was refluxed in absolute ethanol with ethyl 2-chloroacetoacetate (0.28 mL, 2 mmol) for 4 h. At the end of the reaction, the solvent was evaporated and 5 mL of H_2_O was added, followed by neutralization with NH_4_OH to give compound **5** (0.32 g, off-white microcrystals). Compound (**5**) (0.32 g, 1.7 mmol) was subjected to hydrolysis with NaOH (0.272 g, 6.8 mmol) in (THF-H_2_O 3:1) under overnight reflux. At the end of the reaction, THF was evaporated and the pH was adjusted to 6–7 with HCl. After it was filtered, the solid was washed with water and then dried to give compound (**6**) (0.21 g). Compound (**6**) (0.21 g, 1.3 mmol) was refluxed with compound (**3g**) (0.4 g, 1.3 mmol) in dioxane in the presence of one equivalent TEA (0.18 mL). At the end of the reaction, the solvent was evaporated; ethylacetate was added and washed with diluted HCl. The organic layer was then dried over anhydrous sodium sulfate, filtered, and the filtrate was evaporated to give compound (**2j**) (0.40 g, 90%).

*Procedure for the Synthesis of N-acylbenzotriazoles* (**3a**–**k′**): To 0.95 g BtH (8 mmol) dissolved in 50 mL CH_2_Cl_2_, 0.14 mL SOCl_2_ (2 mmol) were added. The mixture was stirred at 25 °C for 30 min, followed by the addition of the corresponding acid **2a**–**k′** (2 mmol) and the reaction was allowed to stir for an additional 3 h at 25 °C. The reaction was diluted with CH_2_Cl_2_ (50 mL) and the organic layer was washed with saturated Na_2_CO_3_ (20 mL, 3×), H_2_O (20 mL, 2×), and brine (10 mL, 1×). The organic layer was dried over anhydrous sodium sulfate. Hexane (50 mL) was added to the filtrate, then the solid obtained was dried under vacuum to give compounds **3a**–**k′**.

*Procedure for the Synthesis of N-acylcephalexines* **4a**–**k′**: Cephalexin sodium salt (0.37 g, 1 mmol) was dissolved in water (1 mL) and added to the solution of the corresponding *N*-acylbenzotriazoles (1 mmol) in CH_3_CN (7 mL). The mixture was stirred for 6–20 h (until complete consumption of *N*-acylbenzotriazole as monitored by TLC). The pH was adjusted to 5 using 2 N HCl, and the solvent was evaporated under reduced pressure. The residue was then extracted with ethyl acetate (20 mL, 2×) and the organic layer was washed with 4 N HCl (10 mL, 3×), water (10 mL, 1×), and brine (10 mL, 1×) then dried over anhydrous sodium sulfate. Hexane (10 mL) was added to the filtrate and the solution was left overnight in the freezer to give the desired products **4a**–**k′**.

*(1H-Benzo[d][1,2,3]triazol-1-yl)(phenyl)methanone* (**3d**): White microcrystals; yield: (0.42 g, 94%); m.p. 111–112 °C, lit. (110–112 °C) [14]. ^1^H-NMR (400 MHz, DMSO-*d*_6_) δ 8.33–8.28 (m, 2H, Ar-H), 8.13–8.10 (m, 2H,Ar-H), 7.85–7.76 (m, 2H, Ar-H), 7.68–7.63 (m, 3H, Ar-H). ^13^C-NMR (100 MHz, DMSO-*d*_6_) δ 166.5 (C=O), 145.2 (C-N=N), 133.5 (Ar-C), 131.7 (Ar-C), 131.5 (Ar-C), 131.3 (Ar-C), 130.7 (Ar-C), 128.3 (Ar-C), 126.6 (Ar-C), 120.0 (Ar-C), 114.4 (Ar-C).

*(1H-Benzo[d][1,2,3]triazol-1-yl)(pyridin-3-yl)methanone* (**3e**): White microcrystals; yield: (0.42 g, 94%); m.p. 101–102 °C, lit. (101–102 °C) [15]. ^1^H-NMR (400 MHz, DMSO-*d*_6_) δ 9.22 (s, 1H, N=CH-C-C=O), 8.90 (d, *J* = 6.5 Hz, 1H, Ar-H), 8.50–8.48 (m, 1H, Ar-H), 8.33 (dd, *J* = 17.6 Hz, 8.4 Hz, 2H,Ar-H), 7.86 (t, *J* = 7.8 Hz, 1H, Ar-H), 7.71–7.66 (m, 2H, Ar-H). ^13^C-NMR (100 MHz, DMSO-*d*_6_) δ 165.3 (C=O), 153.3 (CH-N), 151.3 (N=CH-CC=O), 145.2 (C-N=N), 138.8 (Ar-C), 131.4 (Ar-C), 130.9 (Ar-C), 128.0 (Ar-C), 126.8 (Ar-C), 123.3 (Ar-C), 120.1 (Ar-C), 114.3 (Ar-C).

*(1H-Benzo[d][1,2,3]triazol-1-yl)(3-nitrophenyl)methanone* (**3f**): White microcrystals; yield: (0.48 g, 90%); m.p. 159–162 °C, lit. (155.0–156 °C) [16]. ^1^H-NMR (400 MHz, DMSO-*d*_6_) δ 8.91 (t, *J* = 2.0 Hz, 1H, Ar-H), 8.60–8.57 (m, 1H, Ar-H), 8.54–8.51 (m, 1H, Ar-H), 8.34 (dd, *J* = 16.0, 8.0 Hz, 2H, Ar-H), 7.95 (t, *J* = 8.0 Hz, 1H, Ar-H), 7.89–7.85 (m, 1H, Ar-H), 7.72–7.67 (m,1H, Ar-H). ^13^C-NMR (100 MHz, DMSO-*d*_6_) δ 164.7 (C=O), 147.3 (C-NO_2_), 145.2 (C-N=N),137.2 (C=CH-CH=C-NO_2_), 133.2 (Ar-C), 131.5 (Ar-C), 131.0 (Ar-C), 130.1(Ar-C), 127.5 (Ar-C), 126.8 (Ar-C), 125.9 (Ar-C), 120.1 (Ar-C), 114.4 (Ar-C).

*(1H-Benzo[d][1,2,3]triazol-1-yl)(3,4,5-trimethoxyphenyl)methanone* (**3g**): White microcrystals; yield: (0.6 g, 96%); m.p. 126–128 °C, (126–128 °C) [17]. ^1^H-NMR (400 MHz, DMSO-*d*_6_) δ 8.28 (d, *J* = 10.8 Hz, 2H, Ar-H), 7.83 (t, *J* = 7.8 Hz, 1H, Ar-H), 7.65 (t, *J* = 7.8 Hz, 1H, Ar-H), 7.47 (s, 2H, Ar-H), 3.86 (s, 6H, *m*-(OCH_3_)), 3.82 (s, 3H, *p*-(OCH_3_)).

*(S)-N-(1-(1H-Benzo[d][1,2,3]triazol-1-yl)-3-(1H-indol-3-yl)-1-oxopropan-2-yl)-4-methylbenzenesulfonamide* (**3h**): Brown microcrystals; yield: (0.80 g, 87%); m.p. 175–176 °C; ^1^H-NMR (400 MHz, DMSO-*d*_6_) δ 10.77 (s, 1H, NH), 8.90 (d, *J =* 8.4 Hz, 1H, Ar-H), 8.23 (d, *J =* 8.0 Hz, 1H, Ar-H), 8.03 (d, *J =* 8.4 Hz, 1H, Ar-H), 7.77 (t, *J =* 7.8 Hz, 1H, Ar-H), 7.61 (t, *J =* 7.6 Hz, 1H, Ar-H), 7.45 (d, *J =* 7.6 Hz, 1H, Ar-H), 7.27(d, *J =* 7.2 Hz, 2H, Ar-H), 7.24 (s, 1H, NH-SO_2_), 7.13 (s, 1H, CH-NH-C), 7.02 (t, *J =* 7.4 Hz, 1H, Ar-H), 6.92 (t, *J =* 7.4 Hz, 1H, Ar-H), 6.87 (d, *J =* 8 Hz, 2H, Ar-H), 5.55 (dd, *J =* 8.8, 5.6 Hz, 1H, CH-NH-SO_2_), 3.41 (dd, *J =* 14.4, 5.6 Hz, 1H, CH_2_-CH-NH), 3.12 (dd, *J =* 14.4, 8.8 Hz, 1H, CH_2_-CH-NH), 2.06 (s, 3H, CH_3_). ^13^C-NMR (100 MHz, DMSO-*d*_6_) δ 171.2 (C=O), 145.4 (C-N=N), 142.4 (=C-SO_2_), 136.9 (Ar-C), 136.1 (Ar-C), 131.0 (Ar-C), 130.2 (Ar-C), 128.9 (Ar-C), 126.7 (Ar-C), 126.6 (Ar-C), 126.0 (Ar-C), 124.5 (Ar-C), 120.9 (Ar-C), 120.1 (Ar-C), 118.5 (Ar-C), 117.9 (Ar-C), 113.8 (Ar-C), 111.4 (Ar-C), 108.0 (Ar-C), 55.7 (CH-NH-SO_2_), 28.2 (CH_2_-CH-NH), 20.7 (CH_3_). Anal. Calcd. for C_24_H_21_N_5_O_3_S: C, 62.73; H, 4.61; N, 15.24; S, 6.98; found: C, 62.85; H, 4.68; N, 15.37; S, 7.02.

*N-(2-(1H-Benzo[d][1,2,3]triazole-1-carbonyl)phenyl)-4-methylbenzenesulfonamide* (**3i**): White microcrystals; yield: (0.70 g, 89%); m.p. 148–150 °C; ^1^H-NMR (400 MHz, DMSO-*d*_6_) δ 10.06 (s, 1H, NHSO_2_), 8.29 (t, *J =* 8.2 Hz, 2H, Ar-H), 7.85 (t, *J =* 8.2 Hz, 1H,Ar-H), 7.78 (dd, *J =* 7.6, 1.6 Hz, 1H, Ar-H), 7.66 (t, *J =* 7.2 Hz, 1H,Ar-H), 7.55–7.50 (m, 1H,Ar-H), 7.45 (d, *J =* 8.0 Hz, 2H, Ar-H), 7.38 (t, *J =* 7.6 Hz, 1H, Ar-H), 7.24 (d, *J =* 8.0 Hz, 2H, Ar-H), 7.04 (d, *J =* 8.4 Hz, 1H, Ar-H), 2.27 (s, 3H, CH_3_). ^13^C-NMR (100 MHz, DMSO-*d*_6_) δ 165.7 (C=O), 145.4 (C-N=N), 143.2 (C-NH), 136.2 (Ar-C), 135.3 (Ar-C), 132.6 (Ar-C), 131.3 (Ar-C), 131.3 (Ar-C), 130.5 (Ar-C), 129.4 (Ar-C), 128.6 (Ar-C), 126.6 (Ar-C), 126.4 (Ar-C), 125.5 (Ar-C), 125.2 (Ar-C), 119.9 (Ar-C), 114.3 (Ar-C), 20.8 (CH_3_). Anal. Calcd. for C_20_H_16_N_4_O_3_S: C, 61.21; H, 4.11; N, 14.28; S, 8.17; found: C, 61.39; H, 4.17; N, 14.45; S, 8.29.

*N-(5-(1H-Benzo[d][1,2,3]triazole-1-carbonyl)-4-methylthiazol-2-yl)-3,4,5-trimethoxybenzamide* (**3j**): White microcrystals; yield: (0.74 g, 82%); m.p. 107–110 °C; ^1^H-NMR (400 MHz, DMSO-*d*_6_) δ 8.29 (d, *J =* 8.4 Hz, 2H, Ar-H), 7.83 (t, *J* = 6.8 Hz, 1H, Ar-H), 7.66 (t, *J* = 7.0 Hz, 1H, Ar-H), 7.47 (s, 2H, Ar-H), 7.14 (s, 1H, NH), 3.86 (s, 6H, *m*-(OCH_3_)), 3.82 (s, 3H, *p*-(OCH_3_)) 1.17 (s, 3H, CH_3_). ^13^C-NMR (100 MHz, DMSO-*d*_6_) δ 165.80 (C=O), 152.42 (C=O), 145.2 (N-C-CH_3_), 142.3 (C-OCH_3_), 131.9 (Ar-C), 130.7 (Ar-C), 128.2 (Ar-C), 127.4 (Ar-C), 126.6 (Ar-C), 126.2 (Ar-C), 125.7 (Ar-C), 120.0 (Ar-C), 114.3 (Ar-C), 109.4 (Ar-C), 60.3 (p-OCH_3_), 56.2 (m-OCH_3_), 14.1 (CH_3_). Anal. Calcd. for C_21_H_19_N_5_O_5_S: C, 55.62; H, 4.22; N, 15.44; S, 7.07.; found: C, 55.78; H, 4.30; N, 15.61; S, 7.15.

*(S)-N-(1-(1H-Benzo[d][1,2,3]triazole-1-yl)-1-oxopropan-2-yl)-4-methylbenzenesulfonamide* (**3k**): White microcrystals; yield: (0.62 g, 90%); m.p. 142–144 °C; ^1^H-NMR (400 MHz, DMSO-*d*_6_) δ 8.78 (d, *J* = 7.8 Hz, 1H, NH-SO_2_), 8.26 (d, *J* = 8.2 Hz, 1H, Ar-H), 8.03 (d, *J* = 8.2 Hz, 1H, Ar-H), 7.78 (t, *J* = 7.6 Hz, 1H, Ar-H), 7.63 (t, *J* = 7.6 Hz, 1H, Ar-H), 7.57 (d, *J* = 8.0 Hz, 2H, Ar-H), 7.16 (d, *J* = 8.0 Hz, 2H, Ar-H), 5.35–5.28 (m, 1H, NH-CH-CH_3_), 2.18 (s, 3H, *p*-CH_3_), 1.45(d, *J* = 6.8 Hz, 3H, NH-CH-CH_3_). Anal. Calcd. for C_16_H_16_N_4_O_3_S: C, 55.80; H, 4.68; N, 16.27; S, 9.31; found: C, 55.96; H, 4.76; N, 16.43; S, 9.41.

*(RS)-N-(1-(1H-Benzo[d][1,2,3]triazole-1-yl)-1-oxopropan-2-yl)-4-methylbenzenesulfonamide* (**3k, 3k′**): White microcrystals; yield: (0.62 g, 90%); m.p. 142–144 °C; ^1^H-NMR (400 MHz, DMSO-*d*_6_) δ 8.78 (d, *J* = 8.4 Hz, 1H, NH-SO_2_), 8.27(d, *J* = 8.4 Hz, 1H, Ar-H), 8.03 (d, *J* = 8.4 Hz, 1H, Ar-H), 7.79 (t, *J* = 8.0 Hz, 1H, Ar-H), 7.63 (t, *J* = 8.0 Hz, 1H, Ar-H), 7.57 (d, *J* = 8.0 Hz, 2H, Ar-H), 7.16 (d, *J* = 8.0 Hz, 2H, Ar-H), 5.35–5.27 (m, 1H, NH-CH-CH_3_), 2.18 (s, 3H, *p*-CH_3_), 1.44 (d, *J* = 6.8 Hz, 3H NH-CH-CH_3_). Anal. Calcd. for C_16_H_16_N_4_O_3_S: C, 55.80; H, 4.68; N, 16.27; S, 9.31; found: C, 55.94; H, 4.71; N, 16.60; S, 9.44.

*(6R,7R)-7-((R)-2-(2-(((Benzyloxy)carbonyl)amino)acetamido)-2-phenylacetamido)-3-methyl-8-oxo-5-thia-1-azabicyclo[4.2.0]oct-2-ene-2-carboxylic acid* (**4a**): White microcrystals; yield: (0.47 g, 87%); m.p. 210–212 °C; ^1^H-NMR (400 MHz, DMSO-*d*_6_) δ 13.21 (s, 1H, COOH), 9.35 (d, *J* = 8.4 Hz, 1H, NH-CH(CO)-CH-), 8.53 (d, *J* = 8.4 Hz, 1H, NH-CH(CO)-Ph), 7.49–7.43 (m, 3H, NH-CO-O-, 2Ar-H), 7.35–7.24 (m, 8H, Ar-H), 5.70 (d, *J* = 8.4 Hz, 1H, NH-CH(CO)-Ph), 5.62 (dd, *J* = 8.4, 4.8 Hz, 1H, NH-CH(CO)-CH-), 5.03 (s, 2H, Ph-CH_2_-O-), 4.96 (d, *J* = 4.8 Hz, 1H, -CH-S-), 3.73 (d, *J* = 6.0 Hz, 2H, NH-CH_2_-CO-), 3.46 (d, *J* = 18.0 Hz, 1H, -S-CH_2_-), 3.28 (d, *J* = 18.0 Hz, 1H, -S-CH_2_-), 1.98 (s, 3H, CH_3_). ^13^C-NMR (100 MHz, DMSO-*d*_6_) δ 170.6 (CO-NH-CH), 168.7 (CO-NH-CH-Ph), 163.9 (CO-N-), 163.5 (COOH), 156.5(CO-O-CH_2_), 138.1 (Ar-C), 137.1 (Ar-C), 128.3 (Ar-C), 128.2 (Ar-C), 127.8 (Ar-C), 127.6 (Ar-C), 127.0 (CH_3_-C=C), 122.8 (-C=C-COOH), 65.4 (O-CH_2_-Ph), 58.4 (-CH-S-), 57.1 (CH-CH-S-), 55.4 (CH(CO)Ph), 43.3 (CH_2_-NH-CO-O-), 28.9 (-S-CH_2_-), 19.4 (CH_3_). Anal. Calcd. for C_26_H_26_N_4_O_7_S: C, 57.98; H, 4.87; N, 10.4; S, 5.95; found: C, 58.13; H, 4.91; N, 10.62; S, 6.04.

*(6R,7R)-7-((R)-2-((S)-2-(((benzyloxy)carbonyl)amino)-3-phenylpropanamido)-2-phenylacetamido)-3-methyl-8-oxo-5-thia-1-azabicyclo[4.2.0]oct-2-ene-2-carboxylic acid* (**4b**): White microcrystals; yield: (0.54 g, 86%); m.p. 216–218 °C; ^1^H-NMR (400 MHz, DMSO-*d*_6_) δ 9.37 (d, *J* = 8.4 Hz, 1H, NH-CH(CO)-CH-), 8.83 (d, *J* = 8.4 Hz, 1H, NH-CH(CO)-Ph), 7.56 (d, *J* = 8.4 Hz, 1H, NH-CH(CO)CH_2_-Ph), 7.40–7.19 (m, 15H, Ar-H), 5.74 (d, *J* = 8.4 Hz, 1H, NH-CH(CO)-Ph), 5.63 (dd, *J* = 8.4, 4.4 Hz, 1H, NH-CH(CO)-CH-), 4.96 (d, *J* = 4.4 Hz, 1H, -CH-S-), 4.94 (s, 2H, Ph-CH_2_-O-), 4.50–4.45 (m, 1H, NH-CH(CO)-CH_2_-Ph), 3.46 (d, *J* = 18.4 Hz,1H, -S-CH_2_-), 3.25 (d, *J* = 18.4 Hz, 1H, -S-CH_2_-), 2.96 (dd, *J* = 13.6, 4.0 Hz, 1H, NH-CH(CO)-CH_2_-Ph), 2.71 (dd, *J* = 13.6, 10.4 Hz, 1H, NH-CH(CO)-CH_2_Ph ), 1.97 (s, 3H, CH_3_). ^13^C-NMR (100 MHz, DMSO-*d*_6_) δ 171.2 (CO-NH-CH(CO)-Ph), 170.6 (CO-NH-CH(CO)-Ph), 163.9 (CO-N-), 163.5 (COOH), 155.8 (CO-O-CH_2_), 138.3 (Ar-C), 137.9 (Ar-C), 137.0 (Ar-C), 129.8 (Ar-C), 129.3(Ar-C), 128.3 (Ar-C), 128.2 (Ar-C), 128.0 (Ar-C), 127.7 (Ar-C), 127.4 (Ar-C), 126.8 (Ar-C), 126.2 (Ar-C), 125.8 (CH_3_-C=C), 122.7 (-C=C-COOH), 65.2 (O-CH_2_-Ph), 58.4 (-CH-S-), 57.2 (CH(CO)-CH-S-), 56.0 (CH(CO)Ph), 55.3 (CH(CO)-NH-CO-O-), 28.9 (-S-CH_2_-), 19.4 (CH-CH_2_-Ph), 13.9 (CH_3_). Anal. Calcd. for C_33_H_32_N_4_O_7_S: C, 63.04; H, 5.13; N, 8.91; S, 5.1; found: C, 63.21; H, 5.19; N, 9.04; S, 5.18.

*(6R,7R)-7-((R)-2-((S)-2-(((benzyloxy)carbonyl)amino)-3-(1H-indol-3-yl)propanamido)-2-phenylacetamido)-3-methyl-8-oxo-5-thia-1-azabicyclo[4.2.0]oct-2-ene-2-carboxylic acid* (**4c**): White microcrystals; yield: (0.56 g, 84%); m.p. 168–170 °C; ^1^H-NMR (400 MHz, DMSO-*d*_6_) δ 13.16 (s, 1H, COOH), 10.80 (s, 1H, NH), 9.34 (d, *J* = 8.0 Hz, 1H, NH-CH(CO)-CH-), 8.75 (d, *J* = 8.0 Hz, 1H, NH-CH(CO)-Ph), 7.65 (d, *J* = 8.0 Hz, 1H, NH-CO-O), 7.46 (d, *J* = 8.4 Hz, 1H, Ar-H), 7.37–7.22 (m, 11H, Ar-H), 7.14 (s, 1H, =CH-NH), 7.05 (t, *J* = 7.4 Hz, 1H, Ar-H), 6.95 (t, *J* = 7.4 Hz, 1H, Ar-H), 5.71 (d, *J* = 8.0 Hz, 1H, NH-CH(CO)-Ph), 5.65 (dd, *J* = 8.0, 4.4 Hz, 1H, NH-CH(CO)-CH-), 4.99 (d, *J* = 4.4 Hz, 1H, -CH-S-), 4.95 (s, 2H, Ph-CH_2_-O-), 4.53–4.47 (m, 1H, CH-NH-CO-O-), 3.48 (d, *J* = 18.4 Hz, 1H, -S-CH_2_-), 3.28 (d, *J* = 18.4 Hz, 1H, -S-CH_2_-), 3.09 (dd, *J* = 14.0, 4.4 Hz, 1H, CH_2_-CH-NH-CO-O-), 2.91 (dd, *J* = 14.0, 9.6 Hz, 1H, CH_2_-CH-NH-CO-O-), 1.99 (s, 3H, CH_3_). ^13^C-NMR (100 MHz, DMSO-*d*_6_) δ 171.6 (CO-NH-CH(CO)-Ph), 170.6 (CO-NH-CH(CO)-Ph), 163.9 (CO-N-), 163.5 (COOH), 155.8 (CO-O-CH_2_), 138.2 (Ar-C), 137.0 (Ar-C), 136.1 (Ar-C), 129.8(Ar-C), 128.3 (Ar-C), 128.2 (Ar-C), 127.7 (Ar-C), 127.6 (Ar-C), 127.4 (Ar-C), 127.3 (Ar-C), 126.9(Ar-C), 124.0 (CH_3_-C=C), 122.8 (-C=C-COOH), 120.8 (Ar-C), 118.7 (Ar-C), 118.2 (Ar-C), 111.2 (Ar-C), 109.9 (Ar-C), 65.3 (O-CH_2_-Ph), 58.5 (-CH-S-), 57.2 (CH(CO)-CH-S-), 55.5 (CH(CO)Ph), 55.4 (CH(CO)-NH-CO-O-), 28.9 (-S-CH_2_-), 27.9 (CH_2_-CH-NH-(CO)-O-), 19.4 (CH_3_). Anal. Calcd. for C_35_H_33_N_5_O_7_S: C, 62.96; H, 4.98; N, 10.49; S, 4.8; found: C, 63.14; H, 5.06; N, 10.67; S, 4.91.

*(6R,7R)-7-((R)-2-benzamido-2-phenylacetamido)-3-methyl-8-oxo-5-thia-1-azabicyclo[4.2.0]oct-2-ene-2-carboxylic acid* (**4d**): Yellow microcrystals; yield: (0.38 g, 84%); m.p. 132–134 °C; ^1^H-NMR (400 MHz, DMSO-*d*_6_) δ 13.11 (s, 1H, COOH), 9.27 (d, *J* = 8.0 Hz, 1H, NH-CH(CO)-CH-), 8.86 (d, *J* = 8.0 Hz, 1H, NH-CH(CO)-Ph), 7.92 (d, *J* = 7.2 Hz, 2H, Ar-H), 7.59–7.44 (m, 5H, Ar-H), 7.37–7.28 (m, 3H, Ar-H), 5.89 (d, *J* = 8.0 Hz, 1H, NH-CH(CO)-Ph), 5.66 (dd, *J* = 8.0, 4.4 Hz, 1H, NH-CH(CO)-CH-), 5.00 (d, *J* = 4.4 Hz, 1H, -CH-S-), 3.48 (d, *J* = 18.4 Hz, 1H, -S-CH_2_-), 3.28 (d, *J* = 18.4 Hz, 1H, -S-CH_2_-), 1.99 (s, 3H, CH_3_). ^13^C-NMR (100 MHz, DMSO-*d*_6_) δ 170.7 (CO-NH-CH(CO)-Ph), 166.2 ((CO)-Ph), 163.9 (CO-N-), 163.4 (COOH), 137.7 (Ar-C), 133.8 (Ar-C), 132.8 (Ar-C), 131.4 (Ar-C), 129.7 (Ar-C), 129.2 (Ar-C), 128.5 (Ar-C), 128.2 (Ar-C), 128.1 (Ar-C), 127.7 (Ar-C), 127.5 (CH_3_-C=C), 122.7 (-C=C-COOH), 58.5 (-CH-S-), 57.1 (CH(CO)Ph), 56.5 (CH(CO)-CH-S-), 28.9 (-S-CH_2_-), 19.3 (CH_3_). Anal. Calcd. for C_23_H_21_N_3_O_5_S: C, 61.18; H, 4.69; N, 9.31; S, 7.1; found: C, 61.42; H, 4.76; N, 9.43; S, 7.19.

*(6R,7R)-3-methyl-7-((R)-2-(nicotinamido)-2-phenylacetamido)-8-oxo-5-thia-1-azabicyclo[4.2.0]oct-2-ene-2-carboxylic acid* (**4e**): White microcrystals; yield: (0.37 g, 82%); m.p. 200–202 °C; ^1^H-NMR (400 MHz, DMSO-*d*_6_) δ 9.29 (d, *J* = 8.4 Hz, 1H, NH-CH(CO)-CH-), 9.21 (d, *J* = 8.0 Hz, 1H, NH-CH(CO)-Ph), 9.04 (s, 1H, Ar-H), 8.70 (dd, *J* = 4.8, 1.6 Hz, 1H, Ar-H), 8.27–8.24 (m, 1H, Ar-H), 7.54 (d, *J* = 6.8 Hz, 2H, Ar-H), 7.51–7.48 (m, 1H, Ar-H), 7.38–7.29 (m, 3H, Ar-H ), 5.90 (d, *J* = 8.0 Hz, 1H, NH-CH(CO)-Ph), 5.59 (dd, *J* = 8.4, 4.8 Hz, 1H, NH-CH(CO)-CH-), 4.94 (d, *J* = 4.4 Hz, 1H, -CH-S-), 3.42 (d, *J* = 18.4 Hz, 1H, -S-CH_2_-), 3.18 (d, *J* = 18.4Hz, 1H, -S-CH_2_-), 1.95 (s, 3H, CH_3_). ^13^C-NMR (100 MHz, DMSO-*d*_6_) δ 171.1 (CO-NH-CH(CO)-Ph), 165.5 (CO-NH-CH(CO)-Ph), 164.9 (CO-N-), 163.8 (COOH), 152.5 (C=N-C=C-CO), 149.4 (C=N-C=C-CO), 138.0(Ar-C), 136.0 (Ar-C), 130.0 (Ar-C), 128.8 (Ar-C), 128.7(Ar-C), 128.3 (Ar-C), 128.1 (Ar-C), 127.9 (CH_3_-C=C), 123.8 (-C=C-COOH), 58.8 (-CH-S-), 57.6 (CH(CO)-CH-S-), 57.1 (NH-CH(CO)-Ph), 29.2 (-S-CH_2_-), 19.8 (CH_3_). Anal. Calcd. for C_22_H_20_N_4_O_5_S: C, 58.40; H, 4.46; N, 12.38; S, 7.09; found: C, 58.63; H, 4.53; N, 12.61; S, 7.21.

*(6R,7R)-3-methyl-7-((R)-2-(3-nitrobenzamido)-2-phenylacetamido)-8-oxo-5-thia-1-azabicyclo[4.2.0]oct-2-ene-2-carboxylic acid* (**4f**): Yellow microcrystals; yield: (0.43 g, 87%); m.p. 226–228 °C; ^1^H-NMR (400 MHz, DMSO-*d*_6_) δ 13.44 (s, 1H, COOH), 9.40 (d, *J* = 7.6 Hz, 1H, NH-CH(CO)-Ph), 9.33 (d, *J* = 8.0 Hz, 1H, NH-CH(CO)-CH- ), 8.76–8.62 (m, 1H, Ar-H), 8.48–8.46 (m, 1H, Ar-H), 8.40–8.34 (m, 2H, Ar-H), 7.83–7.75 (m, 2H, Ar-H), 7.54 (d, *J* = 7.2 Hz, 1H, Ar-H), 7.39–7.30 (m, 2H, Ar-H), 5.91 (d, *J* = 7.6 Hz, 1H, NH-CH(CO)-Ph), 5.67 (dd, *J* = 8.0, 4.8 Hz, 1H, NH-CH(CO)-CH-), 5.00 (d, *J* = 4.8 Hz, 1H, -CH-S-), 3.48 (d, *J* = 18.0 Hz, 1H, -S-CH_2_-), 3.28 (d, *J* = 18.0 Hz, 1H, -S-CH_2_-), 1.99 (s, 3H, CH_3_). ^13^C-NMR (100 MHz, DMSO-*d*_6_) δ 170.6 (CO-NH-CH(CO)-Ph), 165.5 (CO-NH-CH(CO)-Ph), 164.5 (CO-N-), 163.6 (COOH), 147.60 (C-NO_2_), 137.4(Ar-C), 135.4 (Ar-C), 134.4 (Ar-C), 132.5 (Ar-C), 130.5 (Ar-C), 129.9 (Ar-C), 128.3 (Ar-C), 127.3 (CH_3_-C=C), 126.1 (Ar-C), 123.7(Ar-C), 122.7(-C=C-COOH), 58.6 (-CH-S-), 57.2 (NH-CH(CO)-Ph), 56.9 (CH(CO)-CH-S-), 28.9 (-S-CH_2_-), 19.4 (CH_3_). Anal. Calcd. for C_23_H_20_N_4_O_7_S: C, 55.64; H, 4.06; N, 11.28; S, 6.46; found: C, 55.78; H, 4.03; N, 11.52; S, 6.52.

*(6R,7R)-3-methyl-8-oxo-7-((R)-2-phenyl-2-(3,4,5-trimethoxybenzamido)acetamido)-5-thia-1-azabicyclo[4.2.0]oct-2-ene-2-carboxylic acid* (**4g**): *Yellow microcrystals; yield:* (0.49 g, 91%); m.p. 197–199 °C; ^1^H-NMR (400 MHz, DMSO-*d*_6_) δ 9.26 (d, *J* = 8.4 Hz, 1H, NH-CH(CO)-CH-), 8.92 (d, *J* = 8.0 Hz, 1H, NH-CH(CO)-Ph), 7.52 (d, *J* = 7.2 Hz, 2H, Ar-H ), 7.37–7.29 (m, 3H, Ar-H), 7.26 (s, 2H, Ar-H), 5.92 (d, *J* = 8.0 Hz, 1H, NH-CH(CO)-Ph), 5.57 (dd, *J* = 8.4, 4.4 Hz, 1H, NH-CH(CO)-CH-), 4.92 (d, *J* = 4.4 Hz, 1H, -CH-S-), 3.83 (s, 6H, M-OCH_3_), 3.70 (s, 3H, P-OCH_3_), 3.39 (d, *J* = 20.0 Hz, 1H, -S-CH_2_-), 3.14 (d, *J* = 20.0 Hz, 1H, -S-CH_2_-), 1.93 (s, 3H, CH_3_). ^13^C-NMR (100 MHz, DMSO-*d*_6_) δ 170.8 (CO-NH-CH(CO)-Ph), 165.8 (CO-NH-CH(CO)-Ph), 164.0 (CO-N-), 163.5 (COOH), 152.5 (m-OCH_3_-C=), 140.2 (p-OCH_3_-C=), 137.8 (Ar-C), 129.7 (Ar-C), 129.0 (Ar-C), 128.2 (Ar-C), 127.7 (Ar-C), 127.6 (CH_3_-C=C), 122.7 (-C=C-COOH), 105.4 (Ar-C), 60.1 (-CH-S-), 58.6 (P-OCH_3_), 57.2 (NH-CH(CO)-Ph), 56.7 (CH(CO)-CH-S-), 56.1 (M-OCH_3_), 28.9 (-S-CH_2_-), 19.4 (CH3). Anal. Calcd. for C_26_H_27_N_3_O_8_S: C, 57.66; H, 5.03; N, 7.76; S, 5.92; found: C, 57.94; H, 5.11; N, 7.88; S, 6.11.

*(6R,7R)-7-((R)-2-((S)-3-(1H-indol-3-yl)-2-(4-methylphenylsulfonamido)propanamido)-2-phenylacetamido)-3-methyl-8-oxo-5-thia-1-azabicyclo[4.2.0]oct-2-ene-2-carboxylic acid* (**4h**): Brown microcrystals; yield: (0.59 g, 86%); m.p. 125–127 °C; ^1^H-NMR (400 MHz, DMSO-*d*_6_) δ 12.56 (s, 1H, COOH), 10.76 (s, 1H, NH), 9.32 (d, *J* = 8.0 Hz, 1H, NH-CH(CO)-CH-), 8.70 (d, *J* = 8.0 Hz, 1H, NH-CH(CO)-Ph), 8.09 (d, *J* = 9.2 Hz, 1H, Ar-H), 7.91 (d, *J* = 8.4 Hz, 1H, Ar-H), 7.46 (d, *J* = 8.0 Hz, 2H, Ar-H), 7.40 (d, *J* = 8.0 Hz, 1H, Ar-H), 7.30–7.24 (m, 4H, 3 Ar-H, 1H, NH-SO_2_ ), 7.17 (d, *J* = 8.0 Hz, 2H, Ar-H), 7.08–7.02 (m, 3H, Ar-H), 6.92 (t, *J* = 7.6, 1H, Ar-H), 5.68 (dd, *J* = 8.0, 4.0 Hz, 1H, NH-CH(CO)-CH-), 5.58 (d, *J* = 8.0 Hz, 1H, NH-CH(CO)-Ph), 4.98 (d, *J* = 4.0 Hz, 1H, -CH-S-), 3.88 (dd, *J* = 8.4, 6.8 Hz, 1H, CH-NH-SO_2_), 3.40 (d, *J* = 8.0 Hz, 1H, -S-CH_2_-), 3.37 (d, *J* = 8.0 Hz, 1H, -S-CH_2_-), 3.04 (dd, *J* = 14.4, 6.8 Hz, 1H, CH_2_-CH-NH-SO_2_), 2.84 (dd, *J* = 14.4, 8.4 Hz, 1H, CH-NH-SO_2_), 2.31 (s, 3H, CH_3_), 2.00 (s, 3H, CH_3_). ^13^C-NMR (100 MHz, DMSO-*d*_6_) δ 172.6 (CO-NH-CH(CO)-Ph), 170.3 (CO-NH-CH(CO)-Ph), 163.9 (CO-N-), 163.4 (COOH), 142.1 (SO_2_-C=), 137.9 (Ar-C), 136.0 (Ar-C), 129.0 (Ar-C), 128.9 (Ar-C), 128.0 (Ar-C), 126.9 (Ar-C), 126.6 (Ar-C), 126.2 (Ar-C), 126.0 (CH_3_-C=C), 123.9 (-C=C-COOH), 120.7 (Ar-C), 120.6 (Ar-C), 118.3 (Ar-C), 117.7 (Ar-C), 111.3 (Ar-C), 108.8 (Ar-C), 64.83 (-CH-S-), 57.18 (CH(CO)-CH-S-), 56.76 (NH-CH(CO)-Ph), 56.48 (CH-NH-SO_2_), 28.61 (CH_2_-CH-NH-SO_2_), 28.22 (-S-CH_2_-), 20.88 (CH_3_), 15.07 (CH_3_). Anal. Calcd. for C_34_H_33_N_5_O_7_S_2_: C, 59.37; H, 4.84; N, 10.18; S, 9.32; found: C, 59.51; H, 4.89; N, 10.26; S, 9.46.

*(6R,7R)-3-methyl-7-((R)-2-(2-(4-methylphenylsulfonamido)benzamido)-2-phenylacetamido)-8-oxo-5-thia-1-azabicyclo[4.2.0]oct-2-ene-2-carboxylic acid*(**4i**): White microcrystals; yield: (0.51 g, 82%); m.p. 137–139 °C; ^1^H-NMR (400 MHz, DMSO-*d*_6_) δ 13.16 (s, 1H, COOH), 11.07 (s, 1H, NHSO_2_), 9.32 (d, *J* = 8.0 Hz, 1H, NH-CH(CO)-CH-), 9.11 (d, *J* = 8.0 Hz, 1H, NH-CH(CO)-Ph), 7.84 (d, *J* = 8.4 Hz, 1H, Ar-H), 7.67 (d, *J* = 8.4 Hz, 1H, Ar-H ), 7.59–7.26 (m, 10H, Ar-H), 7.15–7.10 (m, 1H, Ar-H), 5.81 (d, *J* = 8.0 Hz, 1H, NH-CH(CO)-Ph), 5.68 (dd, *J* = 8.0, 4.0 Hz, 1H, NH-CH(CO)-CH-), 5.01 (d, *J* = 4.0 Hz, 1H, -CH-S-), 3.49 (d, *J* = 18.4 Hz, 1H, -S-CH_2_-), 3.28 (d, *J* = 18.4 Hz, 1H, -S-CH_2_-), 2.30 (s, 3H, CH_3_), 1.98 (s, 3H, CH_3_). ^13^C-NMR (100 MHz, DMSO-*d*_6_) δ 170.1 (CO-NH-CH(CO)-Ph), 167.7 (CO-NH-CH(CO)-Ph), 163.8 (CO-N-), 163.4 (COOH), 137.0 (SO_2_-C=), 129.7 (Ar-C), 129.7 (Ar-C), 129.5 (Ar-C), 129.4 (Ar-C), 128.2 (Ar-C), 127.8 (Ar-C), 127.7 (Ar-C), 126.7(Ar-C), 126.7 (CH_3_-C=C), 122.7 (-C=C-COOH), 121.2 (Ar-C), 120.0 (Ar-C), 58.5 (-CH-S-), 57.1 (NH-CH(CO)-Ph), 56.6 (CH(CO)-CH-S-), 28.8 (-S-CH_2_-), 20.9 (CH_3_), 19.3 (CH_3_). Anal. Calcd. for C_30_H_28_N_4_O_7_S_2_: C, 58.05; H, 4.55; N, 9.03; S, 10.33; found: C, 58.31; H, 4.62; N, 9.14; S, 10.52.

*(6R,7R)-3-methyl-7-((R)-2-(4-methyl-2-(3,4,5-trimethoxybenzamido)thiazole-5-carboxamido)-2-phenylacetamido)-8-oxo-5-thia-1-azabicyclo[4.2.0]oct-2-ene-2-carboxylic acid* (**4j**): Yellow microcrystals; yield: (0.61 g, 89%); m.p. 179–181 °C; ^1^H-NMR (400 MHz, DMSO-*d*_6_) δ 9.28 (d, *J* = 8.0 Hz, 1H, NH-CH(CO)-CH-), 8.88 (d, *J* = 8.0 Hz, 1H, NH-CH(CO)-Ph), 7.56 (s, 1H, NH), 7.52 (d, *J* = 7.2 Hz, 2H, Ar-H), 7.38–7.30 (m, 3H, Ar-H), 7.26 (s, 2H, Ar-H), 5.91 (d, *J* = 8.0 Hz, 1H, NH-CH(CO)-Ph), 5.67 (dd, *J* = 8.0, 4.0 Hz, 1H, NH-CH(CO)-CH-), 5.01 (d, *J* = 4.0Hz, 1H, -CH-S-), 3.83 (s, 6H, m-OCH_3_), 3.71 (s, 3H, p-OCH_3_), 3.48 (d, *J* = 18.4 Hz, 1H, -S-CH_2_-), 3.28 (d, *J* = 18.4 Hz, 1H, -S-CH_2_-), 1.99 (s, 3H, CH_3_), 1.91 (s, 3H, CH_3_). ^13^C-NMR (100 MHz, DMSO-*d*_6_) δ 170.8 (CO-NH-CH(CO)-Ph), 165.7 (CO), 164.0 (CO), 163.4 (COOH), 152.8 (Ar-C), 152.4 (Ar-C), 140.2 (Ar-C), 137.7 (Ar-C), 129.8 (Ar-C), 128.9 (Ar-C), 128.2 (Ar-C), 127.6 (Ar-C), 127.6 (CH_3_-C=C), 122.7 (-C=C-COOH), 122.7 (Ar-C), 105.4 (Ar-C), 60.0 (-CH-S-), 58.5 (p-OCH_3_), 57.2 (CH(CO)-CH-S-), 56.7 (NH-CH(CO)-Ph), 56.0 (m-OCH_3_), 28.9 (-S-CH_2_-), 21.0 (CH_3_), 19.3 (CH_3_). Anal. Calcd. for C_31_H_31_N_5_O_9_S_2_: C, 54.62; H, 4.58; N, 10.27; S, 9.41; found: C, 54.89; H, 4.61; N, 10.38; S, 9.5.

*(6R,7R)-3-methyl-7-((R)-2-(2-((S)-4-methylphenylsulfonamido)propanamido)-2-phenylacetamido)-8-oxo-5-thia-1-azabicyclo[4.2.0]oct-2-ene-2-carboxylic acid* (**4k**): White microcrystals; yield: (0.53 g, 93%); m.p. 234–236 °C; ^1^H-NMR (400 MHz, DMSO-*d*_6_) δ 9.33 (d, *J* = 8.0 Hz, 1H, NH-CH(CO)-CH-), 8.57 (d, *J* = 8.0 Hz, 1H, NH-CH(CO)-Ph), 7.99 (s, 1H, NH-SO_2_), 7.65 (d, *J* = 7.6 Hz, 2H, Ar-H), 7.39 (d, *J* = 7.6 Hz, 2H, Ar-H), 7.35–7.23 (m, 5H, Ar-H), 5.65 (dd, *J* = 8.0, 4.4 Hz, 1H, NH-CH(CO)-CH-), 5.57 (d, *J* = 8.0 Hz, 1H NH-CH(CO)-Ph), 4.95 (d, *J* = 4.4 Hz, 1H, -CH-S-), 4.07–3.95 (m, 1H, NH-CH-CH_3_), 3.44 (d, *J* = 18.0 Hz, 1H, -S-CH_2_-), 3.23 (d, *J* = 18.0 Hz, 1H, -S-CH_2_-), 2.39 (s, 3H, p-CH_3_), 1.97 (s, 3H, CH_3_), 1.03 (d, *J* = 6.8 Hz, 3H, NH-CH-CH_3_).^13^C-NMR (100 MHz, DMSO-*d*_6_) δ 170.8 (CO-NH-CH(CO)-Ph), 170.4 (CO), 163.9 (CO), 163.4 (CO), 142.5 (Ar-C), 138.1(Ar-C), 137.8(Ar-C), 129.4 (Ar-C), 128.2 (Ar-C), 127.6 (Ar-C), 126.9 (Ar-C), 126.6 (Ar-C), 126.5 (CH_3_-C=C), 122.7 (-C=C-COOH), 58.3 (-CH-S-), 57.1 (CH(CO)-CH-S-), 55.3 (NH-CH(CO)-Ph), 51.7 (NH-CH-CH_3_), 28.9 (-S-CH_2_-), 21.0 (p-CH_3_), 19.4 (CH_3_), 18.9 (NH-CH-CH_3_). Anal. Calcd. for C_26_H_28_N_4_O_7_S_2_: C, 54.53; H, 4.93; N, 9.78; S, 11.20; found: C, 54.80; H, 4.96; N, 9.88; S, 11.30.

*(6R,7R)-3-methyl-7-((R)-2-(2-((RS)-4-methylphenylsulfonamido)propanamido)-2-phenylacetamido)-8-oxo-5-thia-1-azabicyclo[4.2.0]oct-2-ene-2-carboxylic acid* (**4k**, **4k′**): White microcrystals; yield: (0.51 g, 89%); m.p. 224–227 °C; ^1^H-NMR (400 MHz, DMSO-*d*_6_) δ 9.35 (d, *J* = 7.6 Hz, 1H, NH-CH(CO)-CH-), 8.57 (d, *J* = 7.6 Hz, 1H, NH-CH(CO)-Ph), 8.01 (d, *J* = 8 Hz, 1H, NH-SO_2_), 7.72–7.58 (m, 2H, Ar-H), 7.45–7.22 (m, 7H, Ar-H), 5.73–5.62 (m, 1H, NH-CH(CO)-CH-), 5.57 (d, *J* = 8.0 Hz, 1H NH-CH(CO)-Ph), 5.46 (d, *J* = 7.6 Hz, 1H, -CH-S-), 4.07–3.92 (m, 1H, NH-CH-CH_3_), 3.45 (d, *J* = 17.2 Hz, 1H, -S-CH_2_-), 3.27 (d, *J* = 17.2 Hz, 1H, -S-CH_2_-), 2.36 (d, *J* = 15.6, 3H, p-CH_3_), 1.98 (s, 3H, CH_3_), 1.07–1.03 (m, 3H, NH-CH-CH_3_). ^13^C-NMR (100 MHz, DMSO-*d*_6_) δ 170.8 (CO-NH-CH(CO)-Ph (k)), 170.7 (CO-NH-CH(CO)-Ph (k′)), 170.4 (CO), 163.9 (CO), 163.4 (CO), 142.6 (Ar-C (k′)), 142.5 (Ar-C (k)), 138.2(Ar-C (k′)), 138.1(Ar-C (k)), 137.8 (Ar-C), 129.9 (Ar-C (k)), 129.8 (Ar-C (k′)), 129.4 (Ar-C (k)), 129.3 (Ar-C (k′)), 128.2 (Ar-C), 126.9 (Ar-C), 126.6 (Ar-C), 126.5 (CH_3_-C=C), 122.7 (-C=C-COOH), 58.4 (-CH-S-), 57.2 (CH(CO)-CH-S-), 55.3 (NH-CH(CO)-Ph), 51.7 (NH-CH-CH_3_ (k)), 51.5 (NH-CH-CH_3_ (k′)), 28.9 (-S-CH_2_-), 21.0 (p-CH_3_ (k)), 20.9 (p-CH_3_ (k′)), 19.4 (CH_3_), 19.1 (NH-CH-CH_3_ (k)), 18.9 (NH-CH-CH_3_(k)). Anal. Calcd. for C_26_H_28_N_4_O_7_S_2_: C, 54.53; H, 4.93; N, 9.78; S, 11.20; found: C, 54.77; H, 4.98; N, 9.86; S, 11.32.

### 2.2. Antimicrobial Activity

#### 2.2.1. Sensitivity Testing (Agar Diffusion Method)

Compounds (**4a**–**j**) and Cephalexin (Ceporex^®^) were dissolved in DMSO in the concentration 1 mg/mL. The microorganisms were cultivated overnight, and optical densities were adjusted 0.2–0.8 and diluted 1:100. Constructed compounds were tested against standard microbial strains in Mueller Hinton agar; Ceporex^®^ and DMSO were tested in parallel as a reference and negative control, respectively. Diameters of inhibition zones were measured in mm.

#### 2.2.2. Minimum Inhibitory Concentration MIC (Broth Dilution Method)

Compounds (**4a-j**) and Cephalexin (Ceporex^®^) were dissolved in DMSO in the concentration 2 mg/mL as a stock solution and then diluted two-fold serially to 1 µg/mL in a Mueller Hinton broth (Sigma-Aldrich, Darmstadt, Germany). The microorganisms were cultivated overnight, and optical densities were adjusted to 0.2–0.8 and diluted 1:100. The constructed compounds were tested against the standard microbial strains; Ceporex^®^ and DMSO were tested in parallel as a reference and negative control, respectively.

## 3. Results and Discussion

### 3.1. Chemistry

**Scheme 1:**
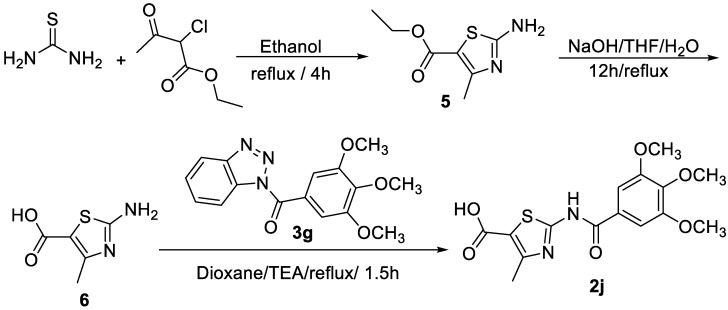
Synthesis of 4-methyl-2-(3,4,5-trimethoxybenzamido)thiazole-5-carboxylic acid (**2j**).

*N*-tosyl-l-tryptophan (**2h**), *N*-tosylanthranilic acid (**2i**), *N*-tosyl-l-alanine (**2k**), and *N*-tosyl-dl-alanine (**2k**, **2k′**) were prepared according to the reported procedure by the reaction of the corresponding amino acid with *p*-toluenesulfonyl chloride in the presence of triethylamine TEA [18]. The presence of the thiazole moiety in many cephalosporin antibiotics motivated us to synthesize 4-methyl-2-(3,4,5-trimethoxy benzamido)thiazole-5-carboxylic acid (**2j**) (Scheme 1) [19]. Condensation of thiourea and ethyl-2-chloroacetoacetate gave ethyl 2-amino-4-methylthiazole-5-carboxylate (**5**) in 95% yield [20]. Compound (**5**) was then hydrolyzed using sodium hydroxide to give the free carboxylic acid (**6**) [21,22]. Coupling of (**6**) with *N*-(3,4,5-trimethoxybenzoyl)benzotriazole (**3g**) afforded 4-methyl-2-(3,4,5-trimethoxybenzamido)thiazole-5-carboxylic acid (**2j**) in 90% yield.

**Scheme 2:**
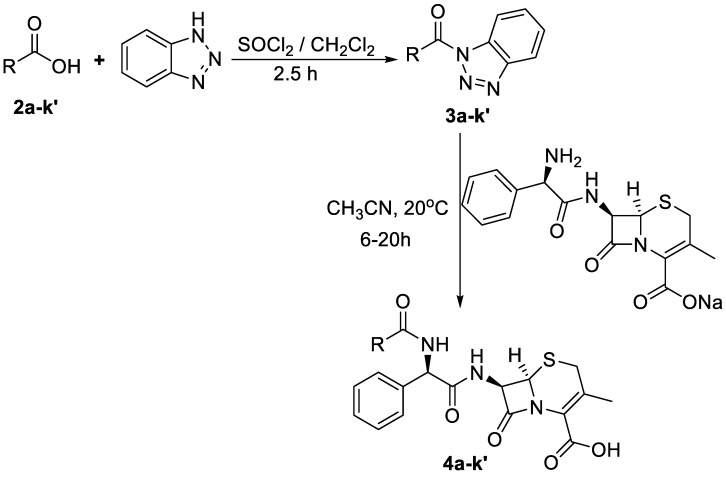
Synthesis of target compounds *N*-acylcephalexins (**4a**–**k′**).

*N*-acylbenzotriazoles **3a**–**k′** (Scheme 2, Table 1) were prepared in 82%–96% yields via the reaction of carboxylic acids **2a**–**k′** with four equivalents of 1*H*-benzotriazole and one equivalent of SOCl_2_ in CH_2_Cl_2_ at 25 °C for 3 h [23]. Subsequently, *N*-acylbenzotriazoles **3a**–**j** and the racemic mixture **3k**, **3k′** were stirred with cephalexin sodium in acetonitrile for 6–20 h at 25 °C to afford *N*-acylcephalexins **4a**–**j** and the diastereomeric mixture **4k**, **4k′** in 82%–92% yield (Scheme 2, Table 1). Novel *N*-acylbenzotriazoles **3h**–**k′** and *N*-acylcephalexins **4a**–**k′** were characterized by ^1^H-NMR, ^13^C-NMR, and elemental analysis. The diastereomeric mixture **4k**, **4k′** was prepared to confirm that the original chirality was maintained during the reaction of cephalexin with chiral *N*-acylbenzotriazoles under the current reaction conditions. The presence of strong absorption at 1764–1770 cm^−1^ in the IR spectra of **4a,b,d** and **g** confirmed that the β-lactam ring was not cleaved during coupling with *N*-acylbenzotriazoles.

### 3.2. Antimicrobial Activity

**Figure 2:**
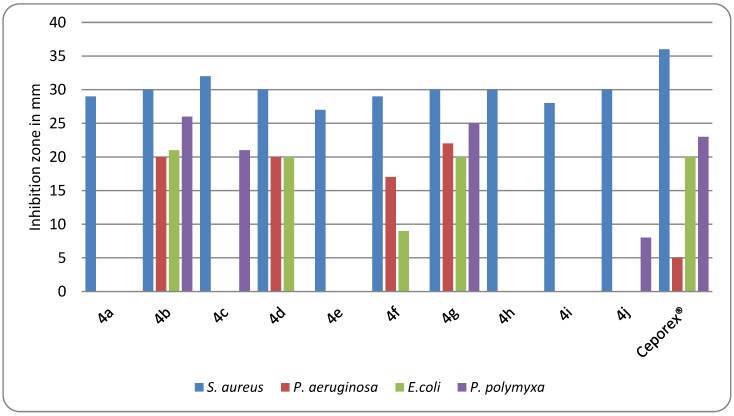
Inhibition measured in mm.

Synthesized compounds **4a**–**j** were tested in vitro for their antimicrobial activity against *Staphylococcus aureus* (ATCC 6538), *Pseudomonas aeruginosa* (ATCC 27853), *Escherichia coli* (ATCC 10536), *Paenibacillus polymyxa* (ATCC 842), and *Candida albicans* (ATCC 10231). Cephalexin (Ceporex^®^) manufactured by: SmithKline Beecham, Harm-Giza, Egypt was used as a reference compound. Sensitivity testing (agar diffusion method) and MIC (broth microdilution method) were performed according to the reported method [26]. The antimicrobial activity of the test compounds as well as cephalexin sodium is depicted in Table 2 and Table 3 and (Figure 2).

All the synthesized compounds **4a**–**j** showed antimicrobial activity against *S.aureus* comparable to Ceporex^®^. Furthermore, compounds **4c,e,g** exhibited bactericidal activity against *E. coli* (G-ve bacteria) and *P. polymyxa* (Gm+ve anaerobic bacteria) close to that of Ceporex^®^. The most interesting result was the strong antimicrobial activity of **4b,c,e,g** against the resistant strain of *P.aeruginosa* which was significantly higher than that of Ceporex^®^. *N*-nicotinylcephalexin (**4c**) and *N*-(3,4,5-trimethoxybenzoyl)cephalexin (**4g**) enjoyed a broader spectrum of antibacterial activity than cephalexin.

## 4. Conclusions

In conclusion, we have utilized a mild and efficient protocol for acylating cephalexin as an example of cephalosporins containing amino nucleophiles using *N*-acylbenzotriazole methodology. The protocol described herein enabled the modulation of the antibacterial activity of cephalosporins. The preliminary antimicrobial susceptibility testing of the novel synthesized targets led to two promising cephalosporin antimicrobials with a broader spectrum of activity.

## Figures and Tables

**Table 1:** RCO-, melting points (m.p.), and yields of *N*-acylbenzotriazoles **3a-k′** and *N*-acylcephalexines **4a-k′**.

RCO-a–k′	3a–k′ Yield %	3a–k′m.p. (°C) m.p. (°C) [Lit.]	Time/h 4a–k′	4a–k′ Yield %	4a–k′ m.p. (°C)
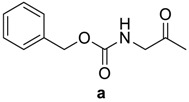	94	107–109 (106–108) [24]	10	87	210–212
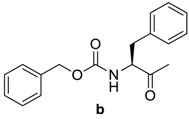	95	150–153 (151–152) [24]	12	86	216–218
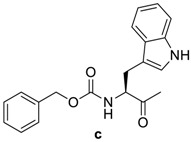	95	99–101 (100–101) [25]	12	84	168–170
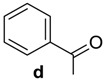	94	111–112 (112–113) [14]	9	84	132–134
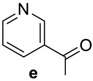	94	101–102 (101–102) [15]	8	82	200–202
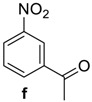	90	156–157 (155–156) [16]	10	87	226–228
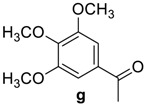	96	125–127 (126–128) [17]	6	91	197–199
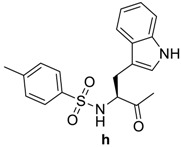	87	175–176	12	86	125–127
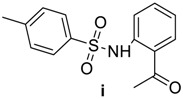	89	148–150	12	82	137–139
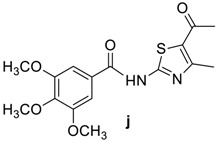	82	107–110	20	89	179–181
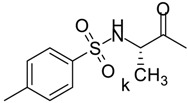	90	142–144	14	93	234–236
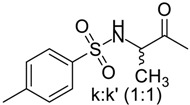	90	142–144	14	89	224–227

**Table 2:** Inhibition zones measured in mm for *N*-acylcephalexins **4a**–**j** and cephalexin (Ceporex^®^).

Target	*Staphylococcus Aureus*	*Pseudomonas Aeruginosa*	*E. coli*	*Paenibacillus Polymyxa*
**4a**	27	-	-	-
**4b**	29	17	9	-
**4c**	30	22	20	25
**4d**	29	-	-	-
**4e**	30	20	20	-
**4f**	32	-	-	21
**4g**	30	20	21	26
**4h**	30	-	-	-
**4i**	28	-	-	-
**4j**	30	-	-	8
**Ceporex**	36 (S)	5 (R)	20 (S)	23 (S)

S: sensitive; R: resistance

**Table 3:** Broth dilution method measured in µg/mL for *N*-acylcephalexins **4a**–**j** and cephalexin (Ceporex®).

Target	*Staphylococcus Aureus*	*Pseudomonas Aeruginosa*	*E. coli*	*Paenibacillus Polymyxa*
**4a**	4	-	-	-
**4b**	2	16	-	-
**4c**	≤1	8	8	4
**4d**	2	-	-	-
**4e**	≤1	8	8	-
**4f**	≤1	-	-	4
**4g**	≤1	8	4	4
**4h**	≤1	-	-	-
**4i**	2	-	-	-
**4j**	≤1	-	-	-
**Ceporex**	≤1 (S)	-	8 (S)	4 (S)

## Supplementary Materials

Detailed NMR spectra are available online at http://www.mdpi.com/2218-0532/84/3/484/s1.

## References

[1] Zucca M., Savoia D. (2010). The Post-Antibiotic Era: Promising Developments in the Therapy of Infectious Diseases. Int. J. Biomed. Sci..

[2] Biek M., Critchley I.A., Riccobene T.A., Thye D.A. (2010). Ceftaroline fosamil: A novel broad-spectrum cephalosporin with expanded anti-Gram-positive activity. J. Antimicrob. Chemother..

[3] Singh S.B., Barrett J.F. (2006). Empirical antibacterial drug discovery—Foundation in natural products. Biochem. Pharmacol..

[4] Tareq F.S., Lee M.A., Lee H.S., Lee Y.J., Lee J.S., Hasan C.M., Islam M.T., Shin H.J. (2014). Gageotetrins A–C., Noncytotoxic Antimicrobial Linear Lipopeptides from a Marine Bacterium Bacillus subtilis. Org. Lett..

[5] Coates A.R., Halls G., Hu Y. (2011). Novel classes of antibiotics or more of the same?. Br. J. Pharmacol..

[6] Heyningen E.V. (1965). The Chemistry of Cephalosporin Antibiotics. III. Acylation of Cephalosporadesates. J. Med. Chem..

[7] Alwan S.M. (2012). Synthesis and Preliminary Antimicrobial Activities of New Arylideneamino-1,3,4-thiadiazole-(thio/dithio)-acetamido Cephalosporanic Acids. Molecules.

[8] Hecker S.J., Calkins T., Price M.E., Huie K., Chen S., Glinka T.W., Dudley M.N. (2003). Prodrugs of Cephalosporin RWJ-333441 (MC-04,546) with Improved Aqueous Solubility. Antimicrob. Agents Chemother..

[9] Watanabe S., Mizukami S., Akimoto Y., Hori Y., Kikuchi K. (2011). Intracellular Protein Labeling with Prodrug-Like Probes Using a Mutant β-Lactamase Tag. Chem. A Eur. J..

[10] Daloia E., Lim G., Melton J., Roubie J.A. (1993). Convenient Method for the Preparation of (Z)-(2-Aminothiazol-4-yl)-2-methoxyimino-acetylchloride Hydrochloride. Synth. Commun..

[11] Saud M.D., Muna I.K., Huda A.H., Ikbal R.H. (2014). Synthesis of New Derivatves of β-Lactam Antibiotics. Int. J. PharmTech Res..

[12] Katritzky A.R., Suzuki K., Wang Z. (2005). Acylbenzotriazoles as Advantageous *N*-, *C*-, *S*-, and *O*-Acylating Agents. Synlett.

[13] Ibrahim M.A., Panda S.S., Birs A.S., Serrano J.C., Gonzalez C.F., Alamry K.A., Katritzky A.R. (2014). Synthesis and antibacterial evaluation of amino acid–antibiotic conjugates. Bioorg. Med. Chem. Lett..

[14] Katritzky A.R., Vakulenko A., Jain R. (2003). The preparation of *N*-acylbenzotriazoles from aldehydes. Arkivoc.

[15] El-Nachef C., Bajaj K., Koblick J., Katritzky A.R. (2012). Microwave-Assisted Formation of Peptide–Vitamin Conjugates. Eur. J. Org. Chem..

[16] Wet-osot S., Duangkamol C., Pattarawarapan M., Phakhodee W. (2015). Facile synthesis of *N*-acylbenzotriazoles from carboxylic acids. Monatshefte Chem..

[17] Agarwal P.K., Dathi M.D., Saifuddin M., Kundu B. (2012). Engineering of indole-based tethered biheterocyclic alkaloid meridianin into β-carboline-derived tetracyclic polyheterocycles via amino functionalization/6-endo cationic π-cyclization. Beilstein J. Org. Chem..

[18] Simsek S., Horzella M., Kalesse M. (2007). Oxazaborolidinone-Promoted Vinylogous Mukaiyama Aldol Reactions. Org. Lett..

[19] Chantot J.F., Bryskier A. (1985). HR 810 (cefpirome). Experimental evaluation of the in vitro and in vivo antibiotic activity of a new amino-2-thiazole methoxy- imino cephalosporin. Pathol. Biol..

[20] Oniga S., Oniga O., Chirtoc I., Ionescu M., Tiperciuc B., Ghiran D. (2005). Activitatea antimicrobiană a unor 3-*N*-acetil-5[2′-(acetil-amino)-4′metil- 5′tiazolil]-2-aril-Δ4-1,3,4-oxadiazoline. Farmacia.

[21] Gharat L.A., Narayana L., Thomas A., Khairatkar N., Shah D.M. (2010). Thiazole Derivatives as Stearoyl CoA Desaturase Inhibitors.

[22] Fu J., Hou D., Kamboj R. (2008). Aminothiazole Derivatives as Human Stearoyl-Coa Desaturase Inhibitors.

[23] Katritzky A.R., Abo-Dya N.E., Tala S.R., Ghazvini-Zadeh E.H., Bajaj K., El-Feky S.A. (2010). Efficient and selective syntheses of *S*-Acyl and *N*-Acyl glutathiones. Synlett.

[24] Katritzky A.R., Abo-Dya N.E., Tala S.R., Gyanda K., Abdel-Samii Z.K. (2009). An efficient method for the preparation of peptide alcohols. Org. Biomol. Chem..

[25] Katritzky A.R., Tala S.R., Abo-Dya N.E., Ibrahim T.S., El-Feky S.A., Gyanda K., Pandya K.M. (2011). Chemical Ligation of *S*-Scylated Cysteine Peptides to Form Native Peptides via 5-, 11-, and 14-Membered Cyclic Transition States. J. Org. Chem..

[26] Luber P., Bartelt E., Genschow E., Wagner J., Hahn H. (2003). Comparison of Broth Microdilution, E Test, and Agar Dilution Methods for Antibiotic Susceptibility Testing of *Campylobacter jejuni* and *Campylobacter coli*. J. Clin. Microbiol..

