# Supplementary material for: Benzotriazole-Mediated Synthesis and Antibacterial Activity of Novel N-Acylcephalexins
Source: Sci Pharm. 2016 Apr 13;84(3):484–96. doi: 10.3390/scipharm84030484 (PMC5064239; doi:10.3390/scipharm84030484)
Supplement: Supplementary file 1 [file scipharm-84-00484-s001.pdf]

# Supplementary Materials: Benzotriazole-Mediated Synthesis and Preliminary Antibacterial Activity of Novel *N*-Acylcephalexins

**Khalid A. Agha, Nader E. Abo-Dya, Tarek S. Ibrahim, Eatedal H. Abdel-Aal and Wael A. Hegazy**

**Figure S1.** $^{1}$H and $^{13}$C NMR spectra of compounds **7d–j**.

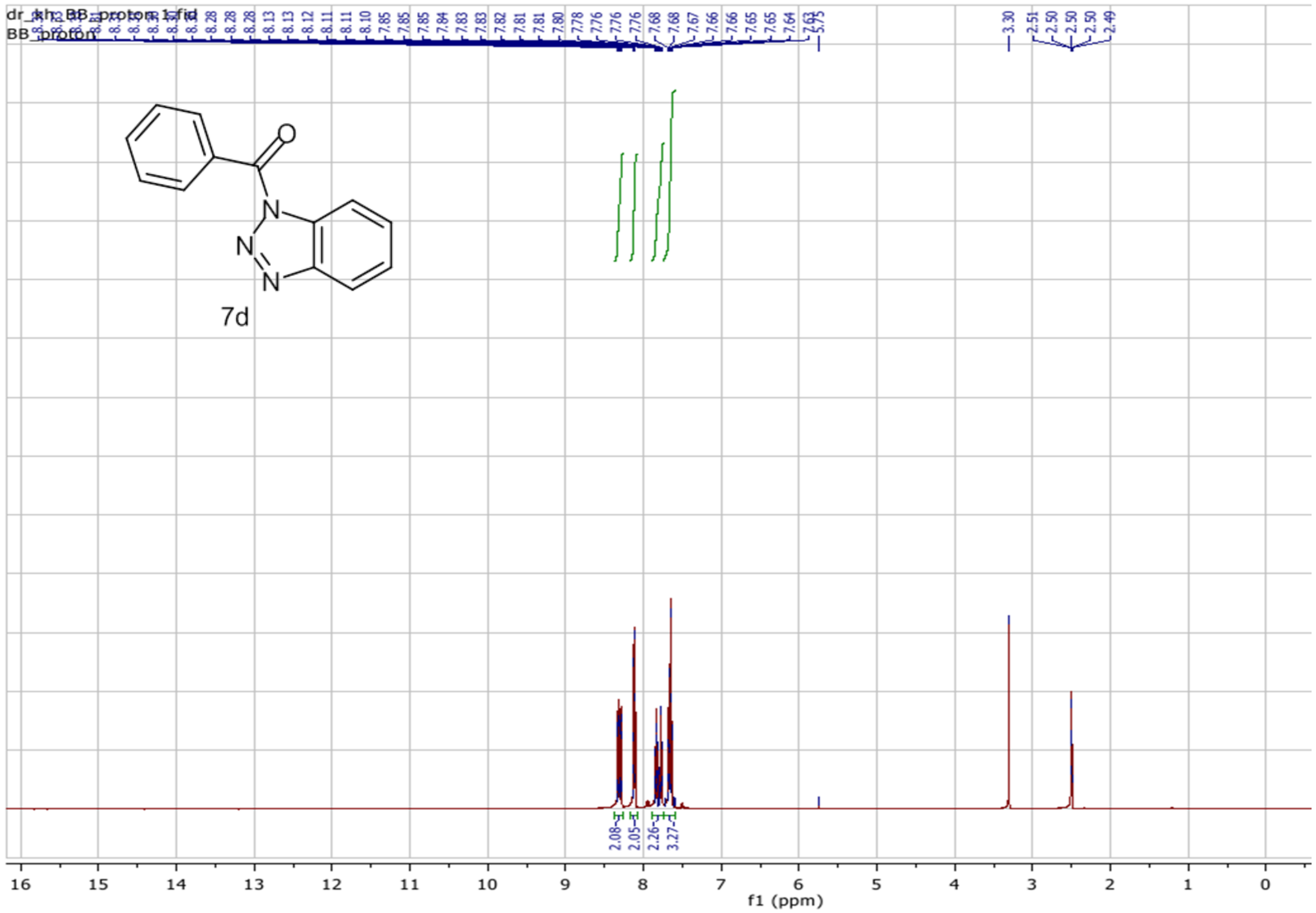

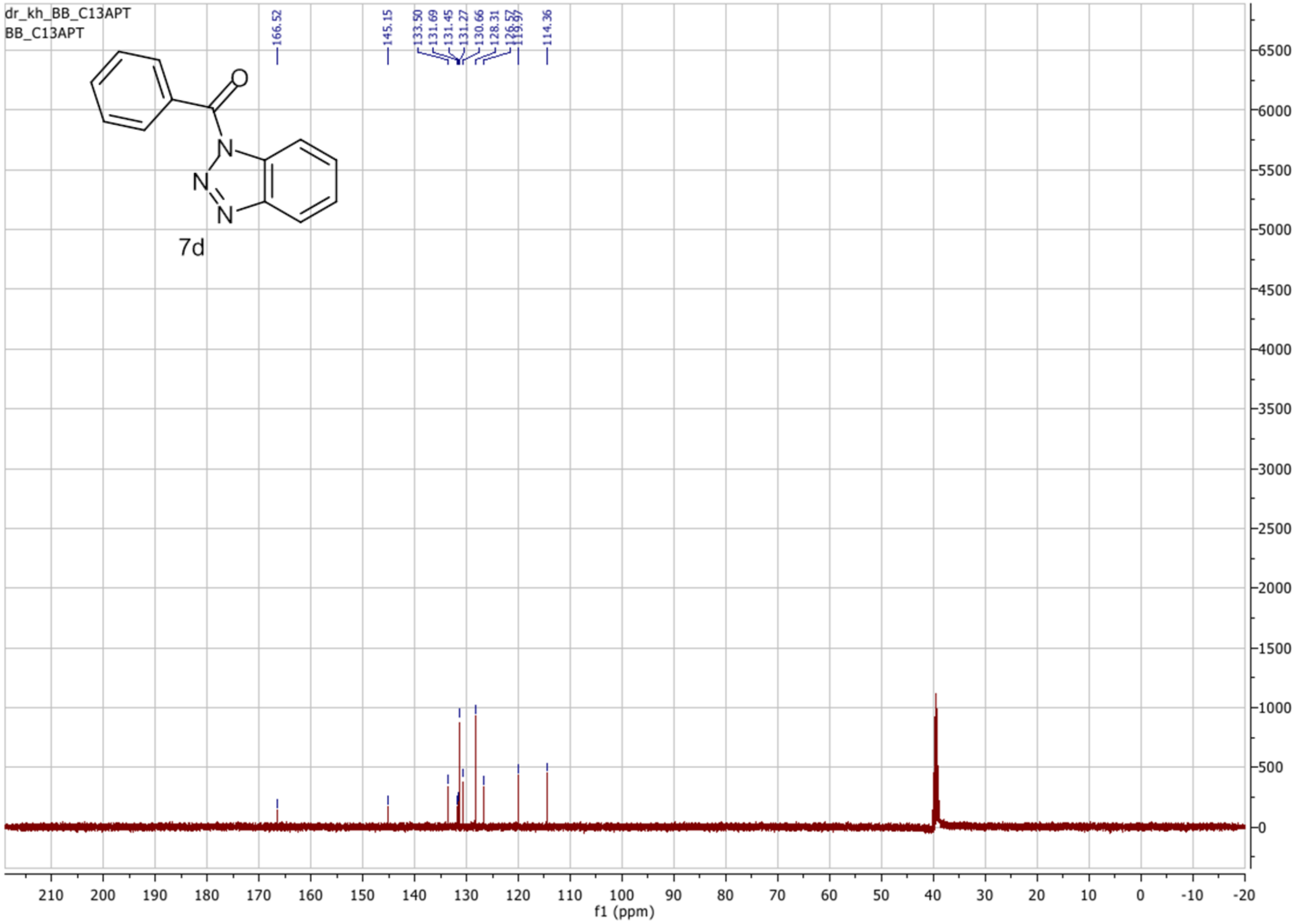
dr_kh_BB_C13APT
BB_C13APT
7d
166.52
145.15
133.50
131.69
131.45
131.27
130.66
128.31
126.57
119.97
114.36
f1 (ppm)

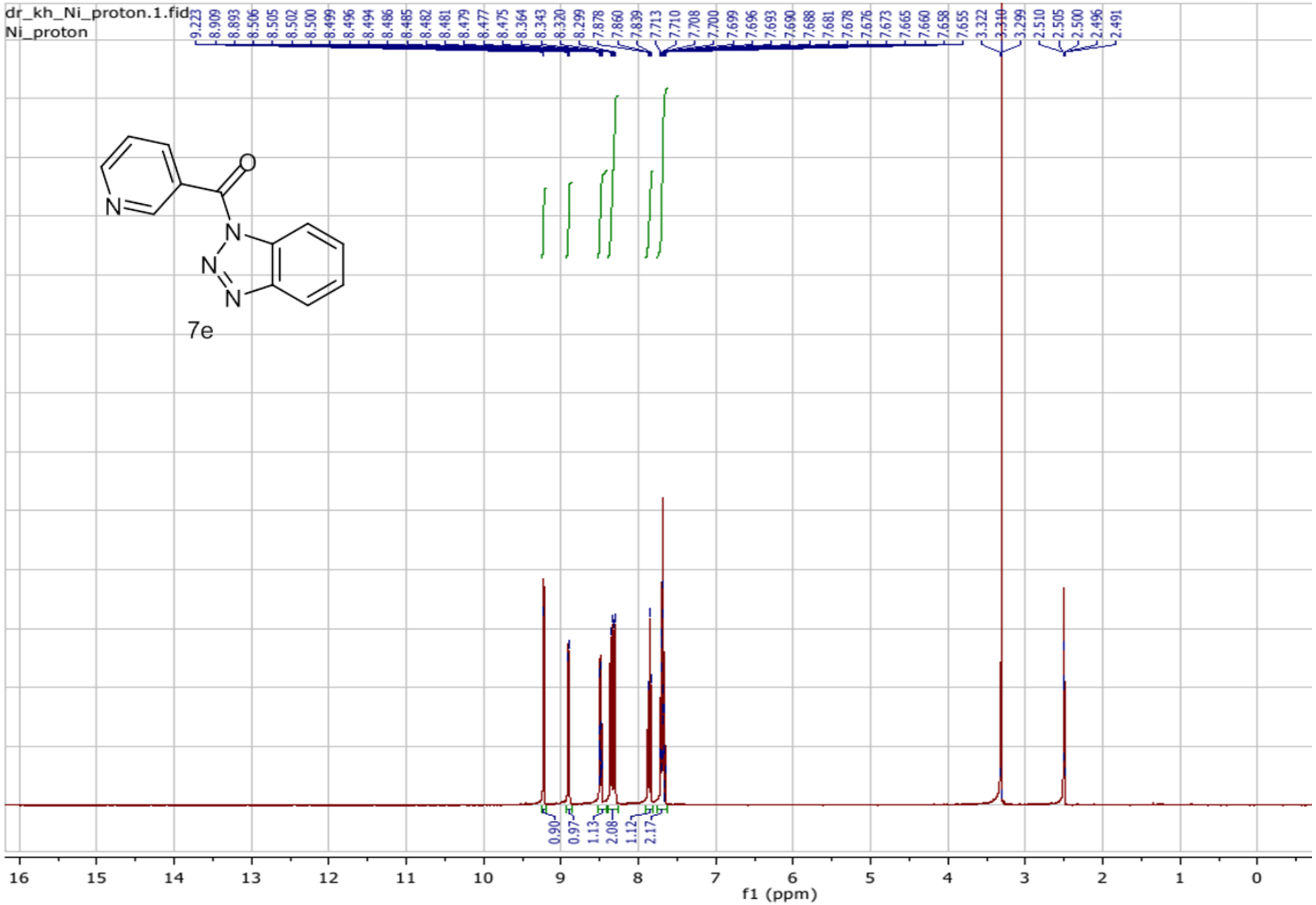
dr_kh_Ni_proton.1.fid
Ni_proton
9.223
8.909
8.893
8.506
8.505
8.502
8.500
8.499
8.496
8.494
8.486
8.485
8.482
8.481
8.479
8.477
8.475
8.364
8.343
8.320
8.299
7.878
7.860
7.839
7.713
7.710
7.708
7.700
7.699
7.696
7.693
7.690
7.688
7.681
7.678
7.676
7.673
7.665
7.660
7.658
7.655
3.322
3.310
3.299
2.510
2.505
2.500
2.496
2.491
0.90
0.97
1.13
2.08
1.12
2.17
16
15
14
13
12
11
10
9
8
7
6
5
4
3
2
1
0
f1 (ppm)
7e

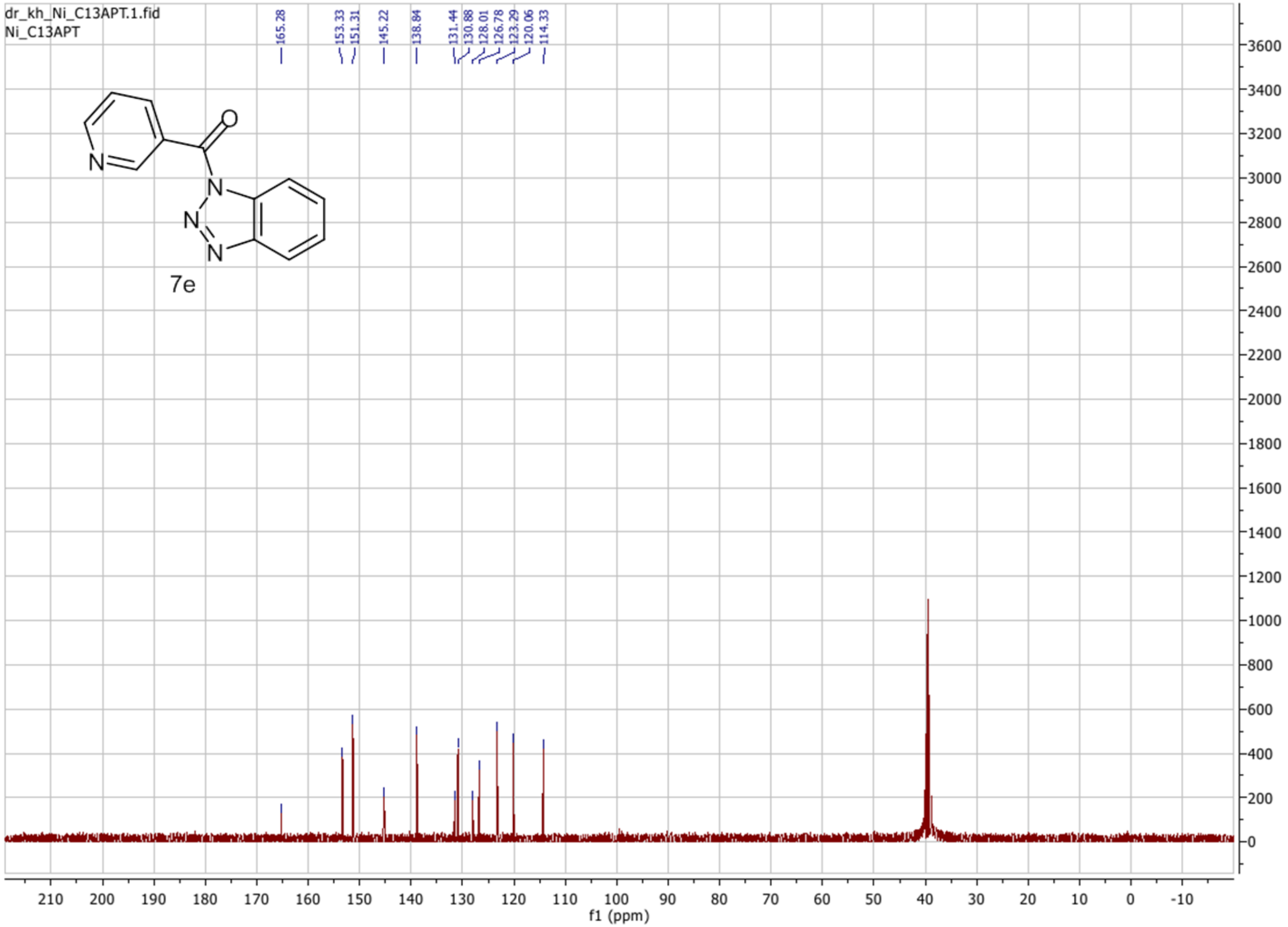
dr_kh_Ni_C13APT.1.fid
Ni_C13APT
165.28
153.33
151.31
145.22
138.84
131.44
130.88
128.01
126.78
123.29
120.06
114.33
7e
f1 (ppm)

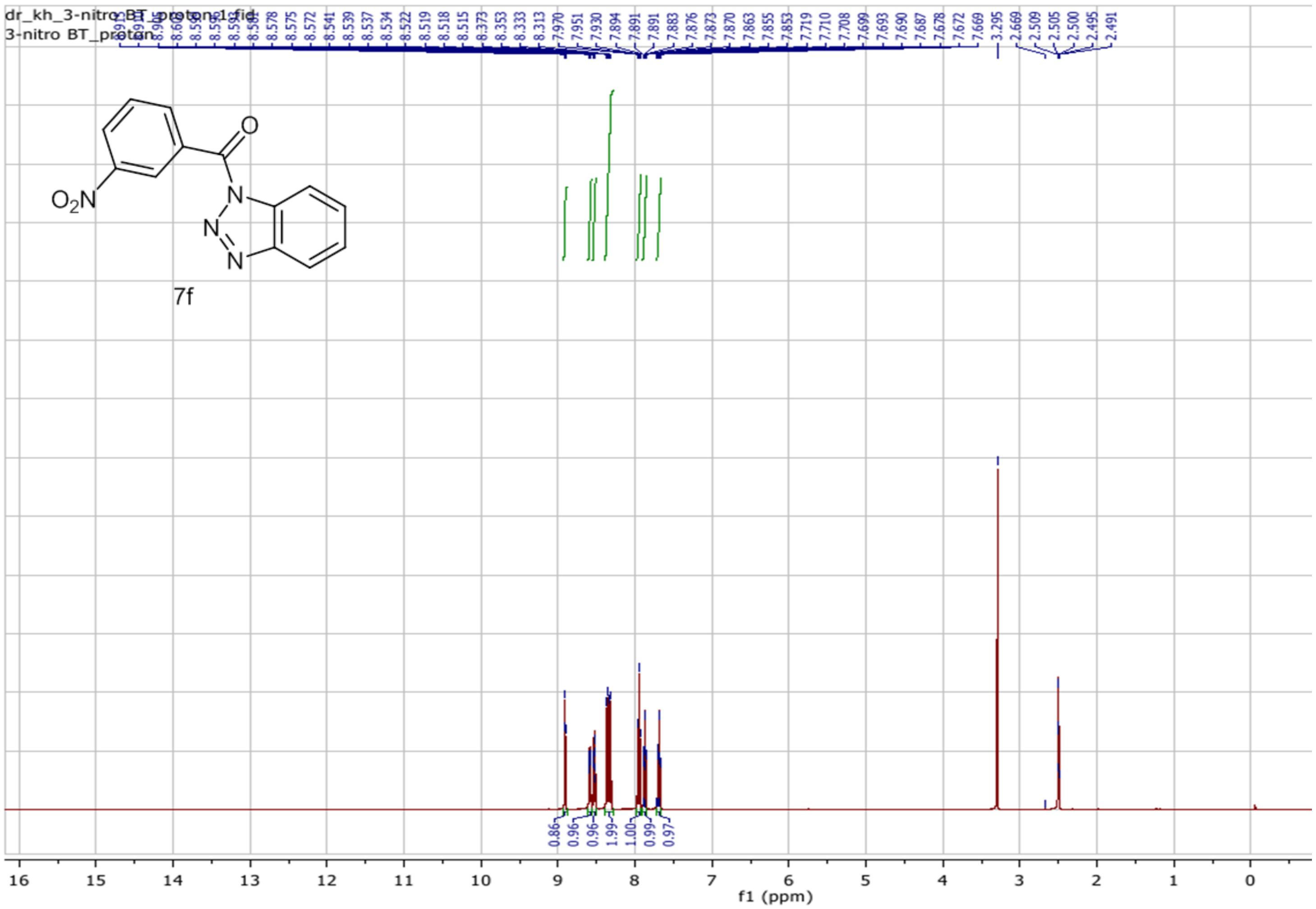
dr_kh_3-nitro BT_proton.1.fid
3-nitro BT_proton
7f
f1 (ppm)

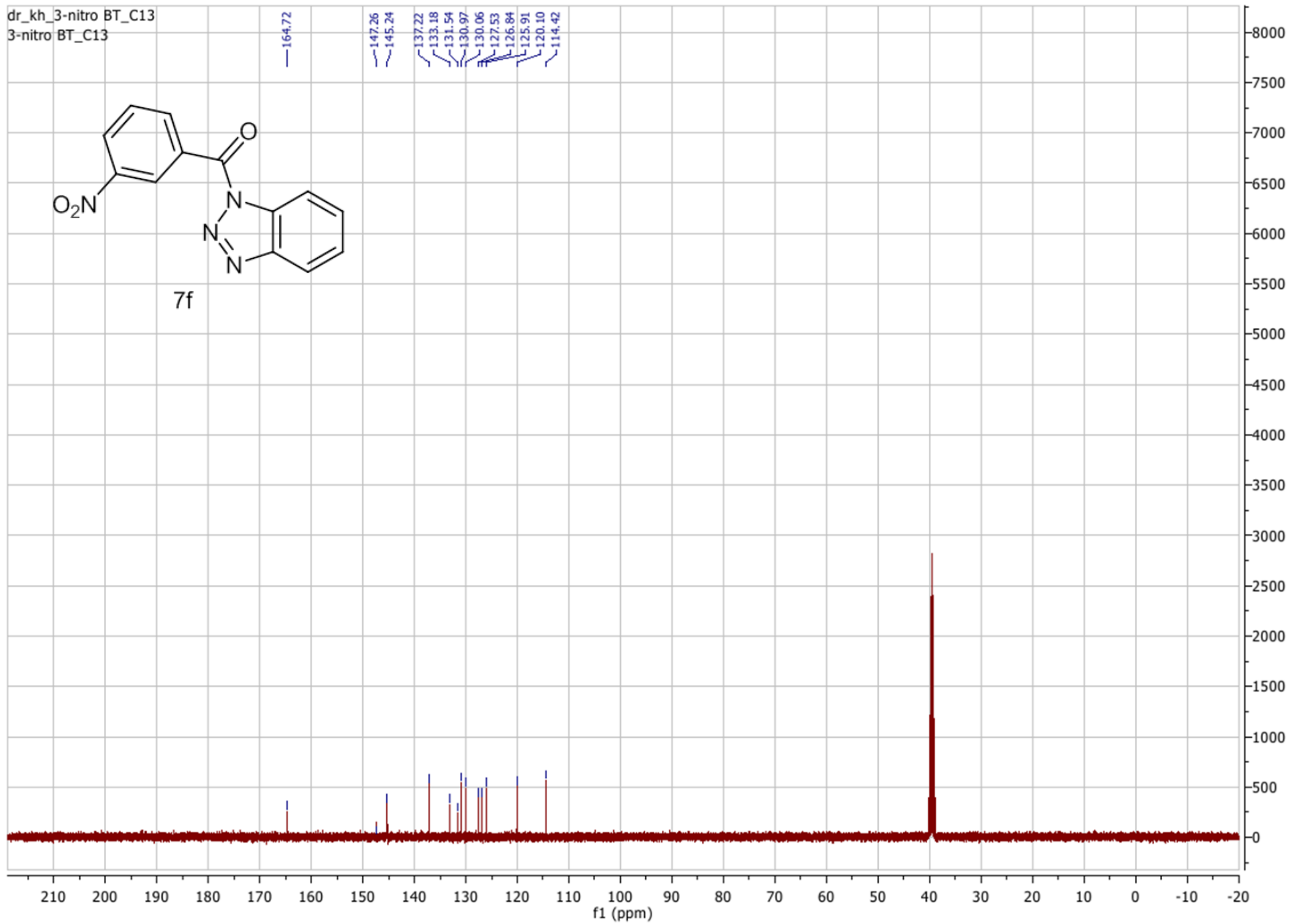
dr_kh_3-nitro BT_C13
3-nitro BT_C13
164.72
147.26
145.24
137.22
133.18
131.54
130.97
130.06
127.53
126.84
125.91
120.10
114.42
7f
f1 (ppm)

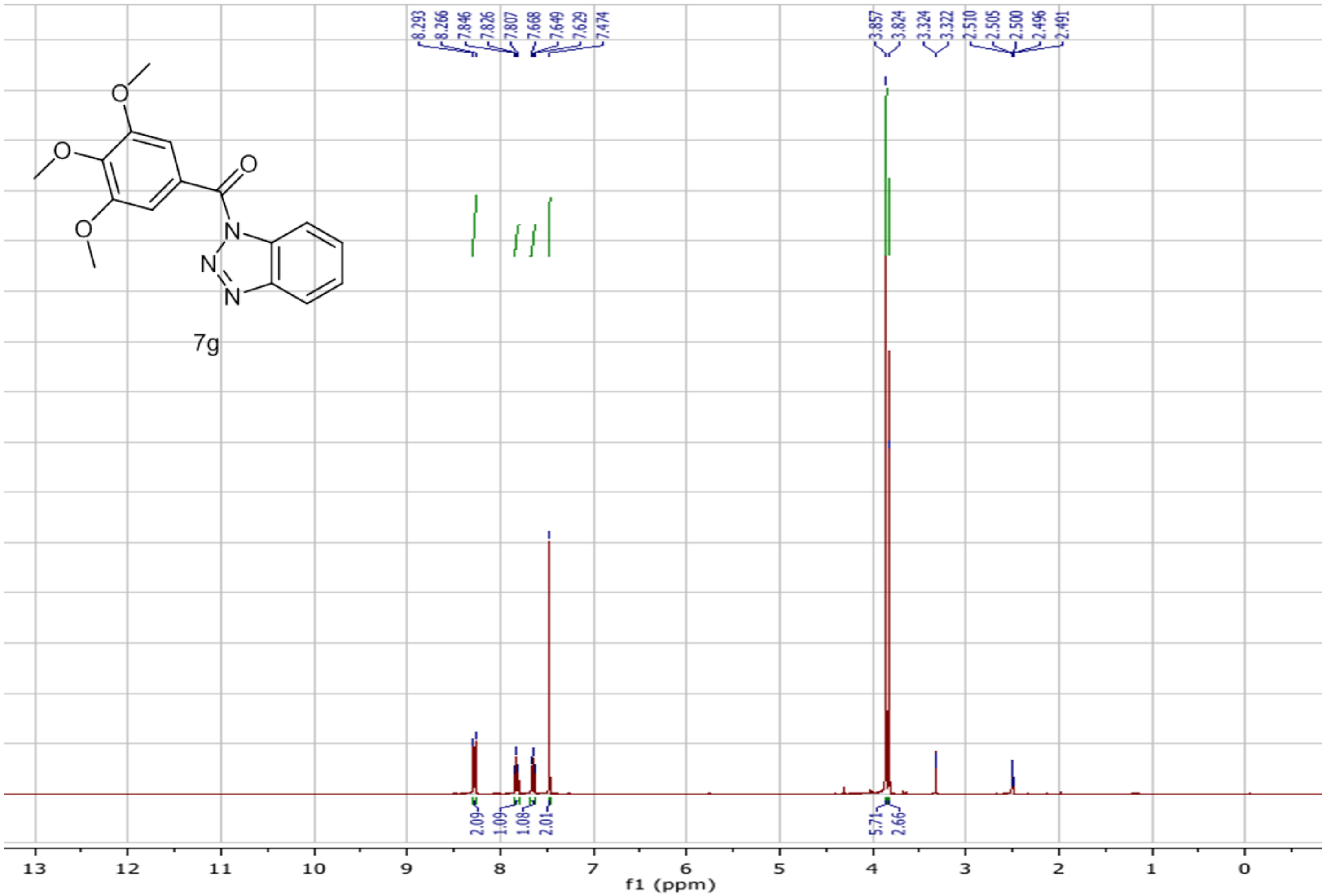
7g
8.293
8.266
7.846
7.826
7.807
7.668
7.649
7.629
7.474
3.857
3.824
3.324
3.322
2.510
2.505
2.500
2.496
2.491
2.09
1.09
1.08
2.01
5.71
2.66
f1 (ppm)

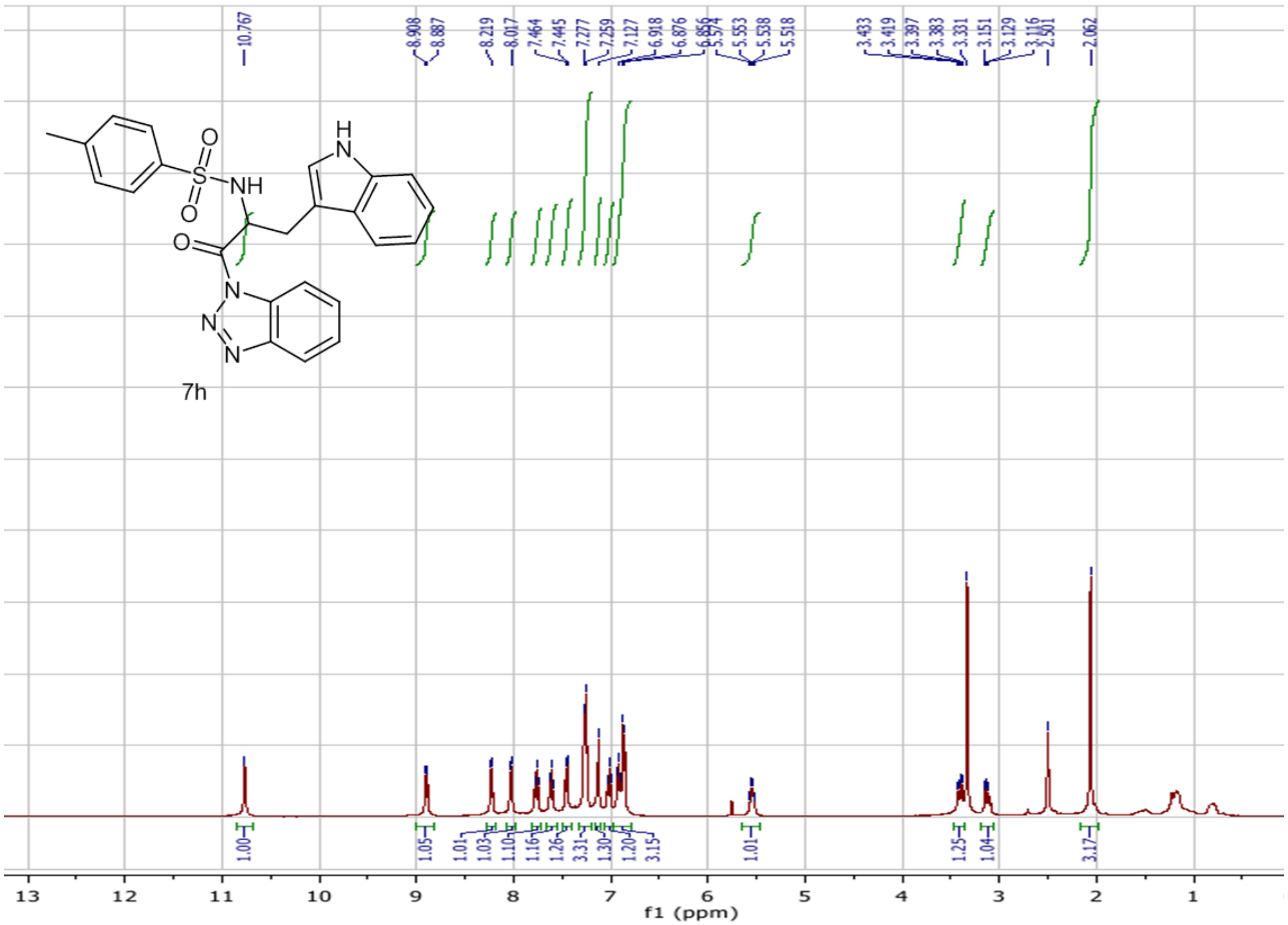

7h

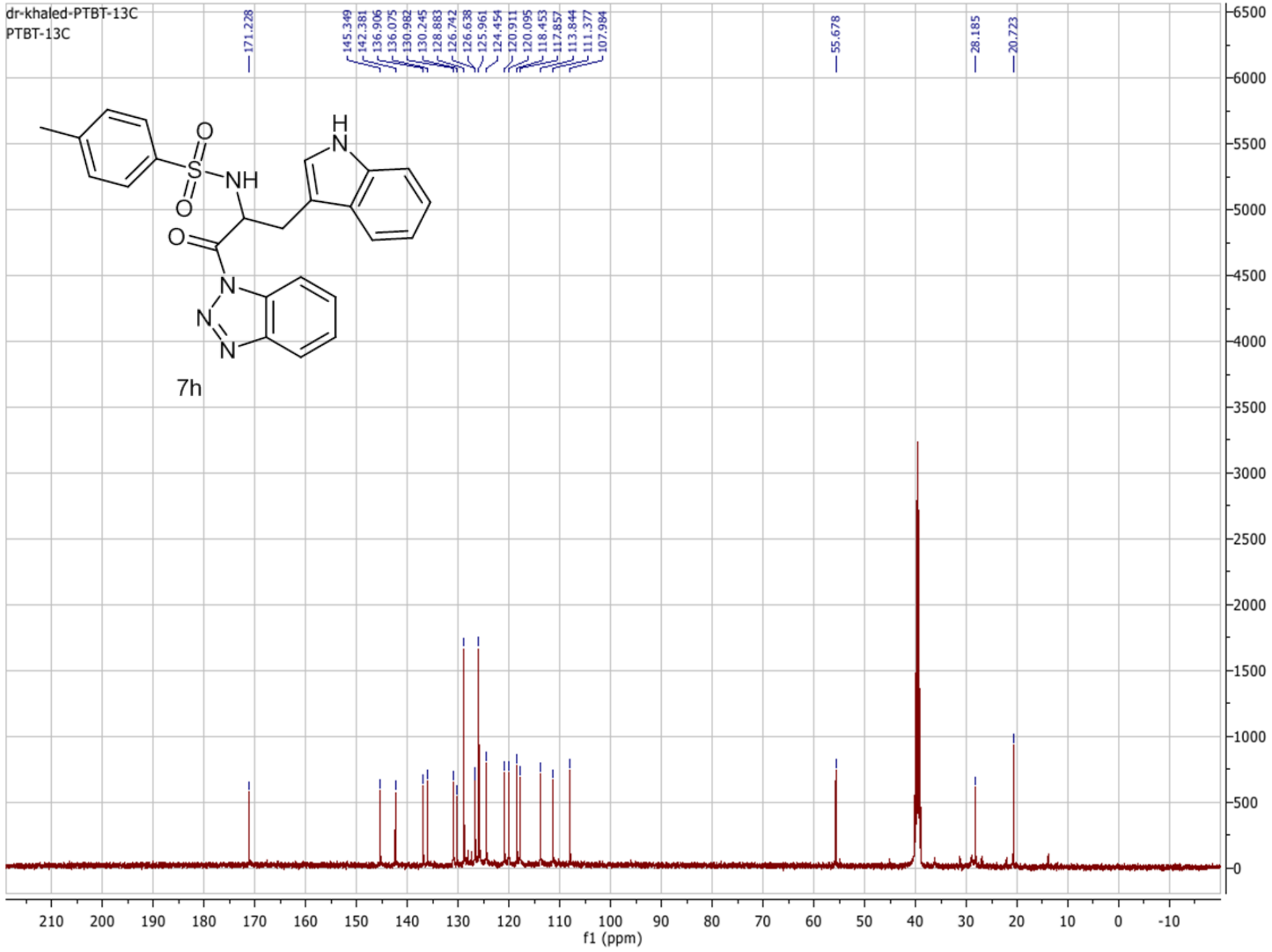
dr-khaled-PTBT-13C
PTBT-13C
171.228
145.349
142.381
136.906
136.075
130.982
130.245
128.883
126.742
126.638
125.961
124.454
120.911
120.095
118.453
117.857
113.844
111.377
107.984
55.678
28.185
20.723
7h
f1 (ppm)

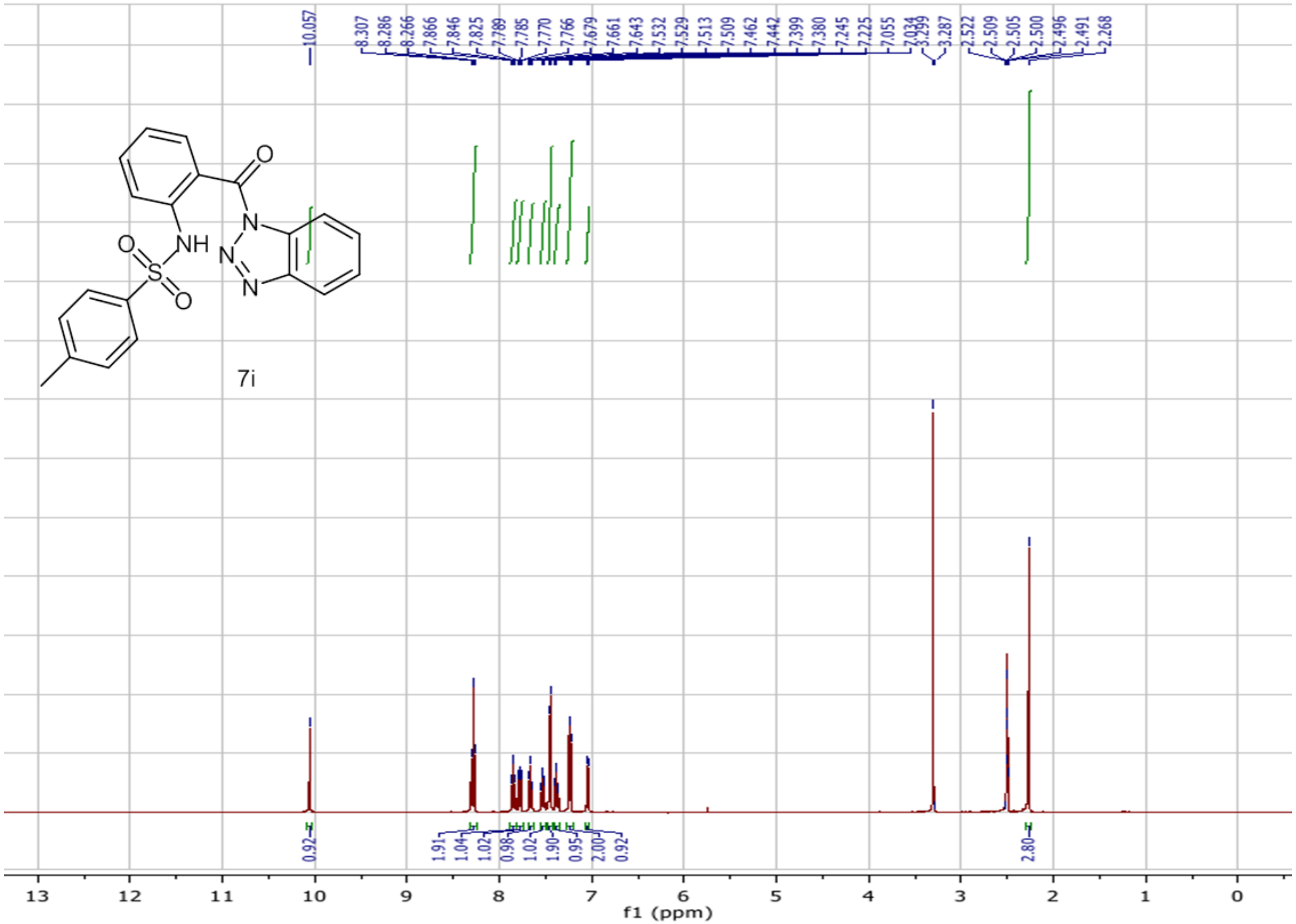
7i
f1 (ppm)

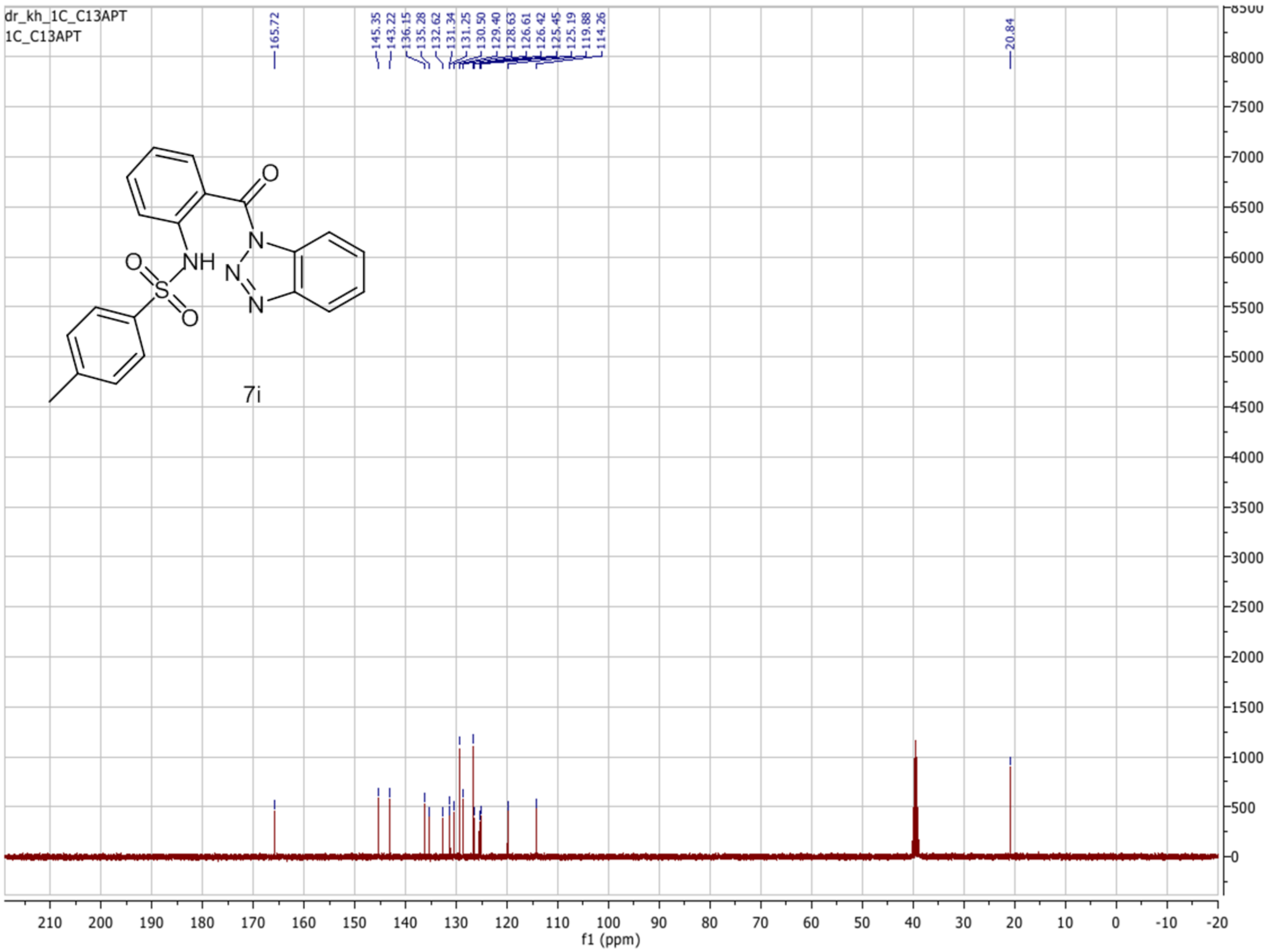
dr_kh_1C_C13APT
1C_C13APT
165.72
145.35
143.22
136.15
135.28
132.62
131.34
131.25
130.50
129.40
128.63
126.61
126.42
125.45
125.19
119.88
114.26
20.84
7i
f1 (ppm)

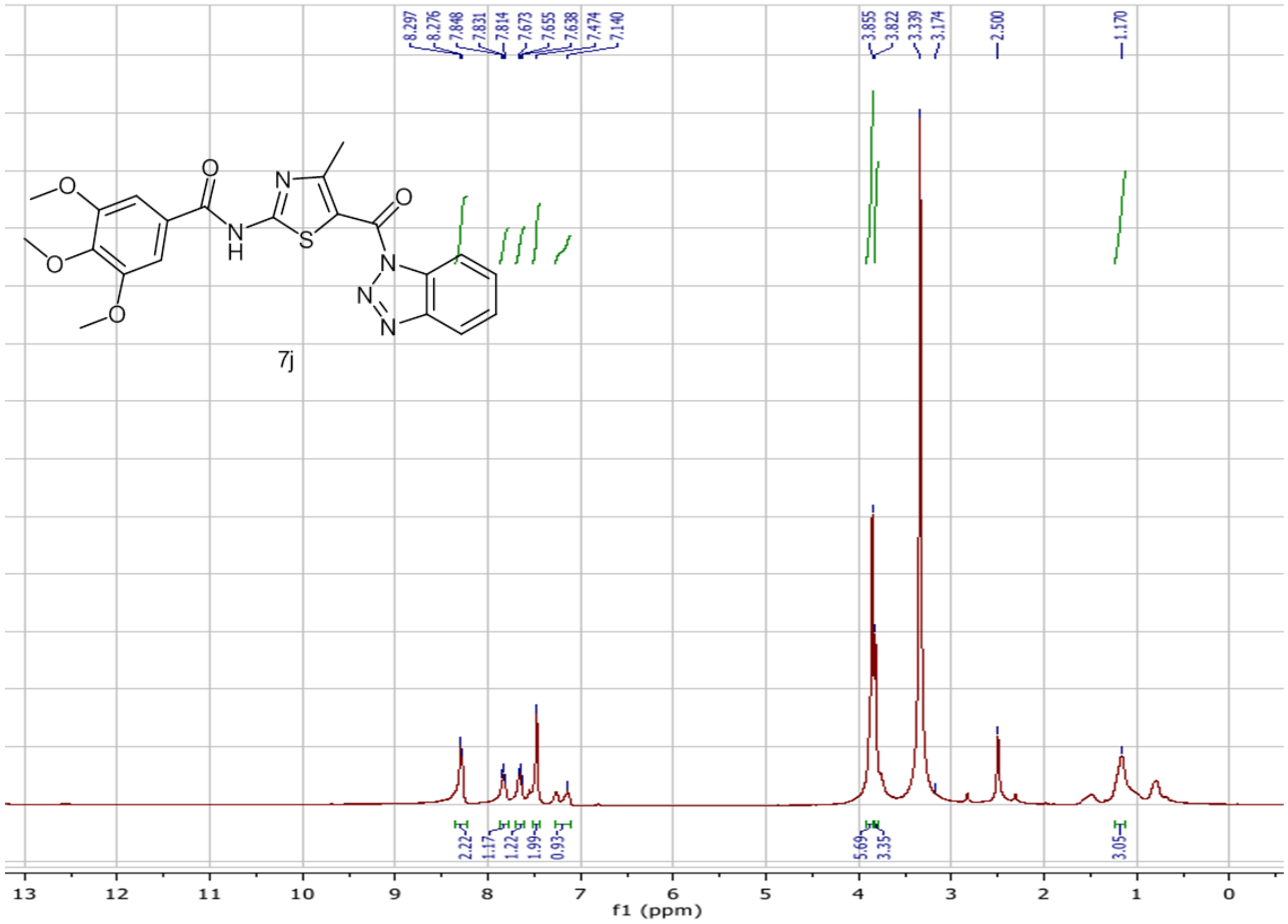

7j
8.297
8.276
7.848
7.831
7.814
7.673
7.655
7.638
7.474
7.140
3.855
3.822
3.339
3.174
2.500
1.170
2.22
1.17
1.22
1.99
0.93
5.69
3.35
3.05
f1 (ppm)

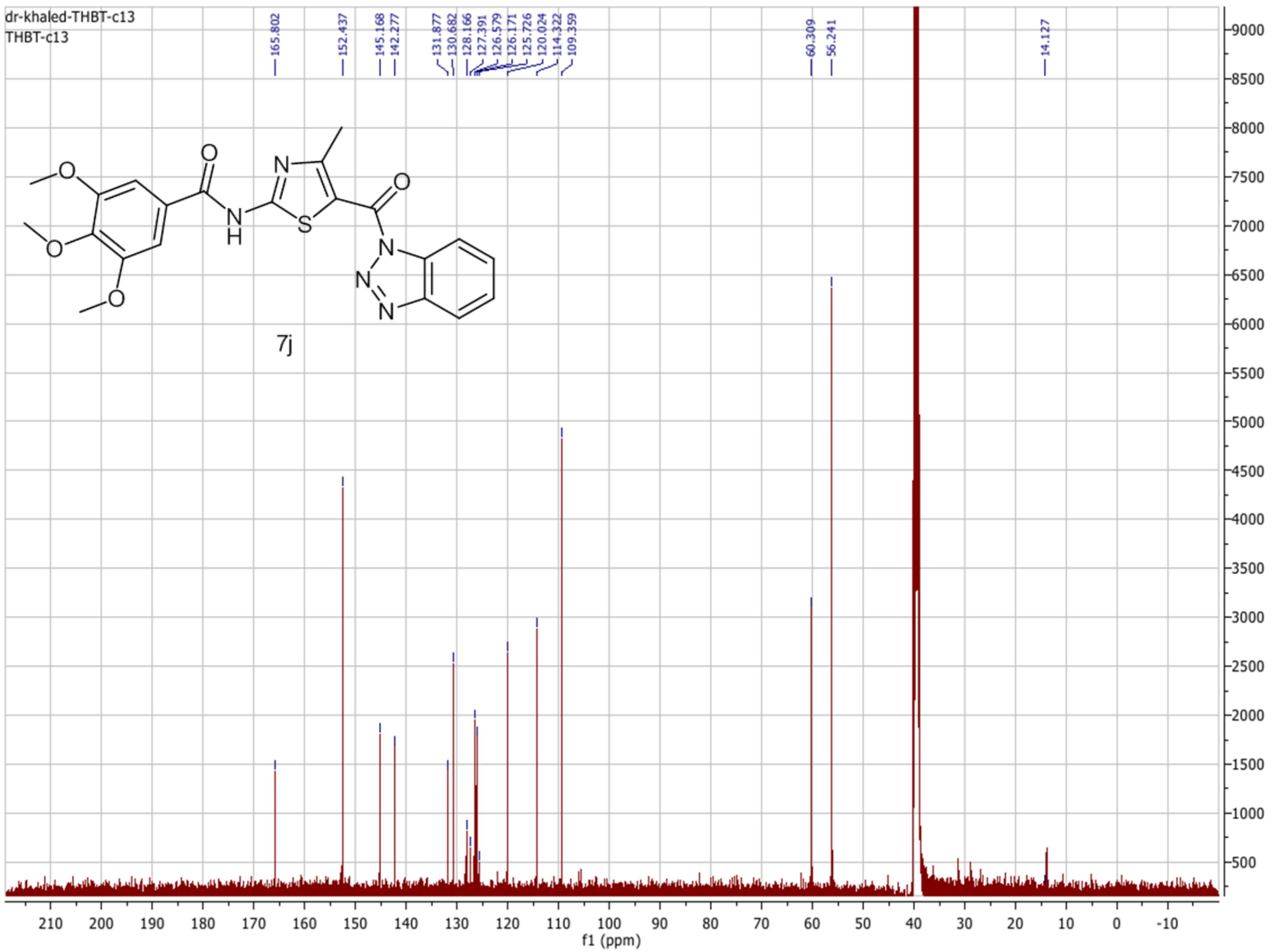
dr-khaled-THBT-c13
THBT-c13
165.802
152.437
145.168
142.277
131.877
130.682
128.166
127.391
126.579
126.171
125.726
120.024
114.322
109.359
60.309
56.241
14.127
7j
f1 (ppm)

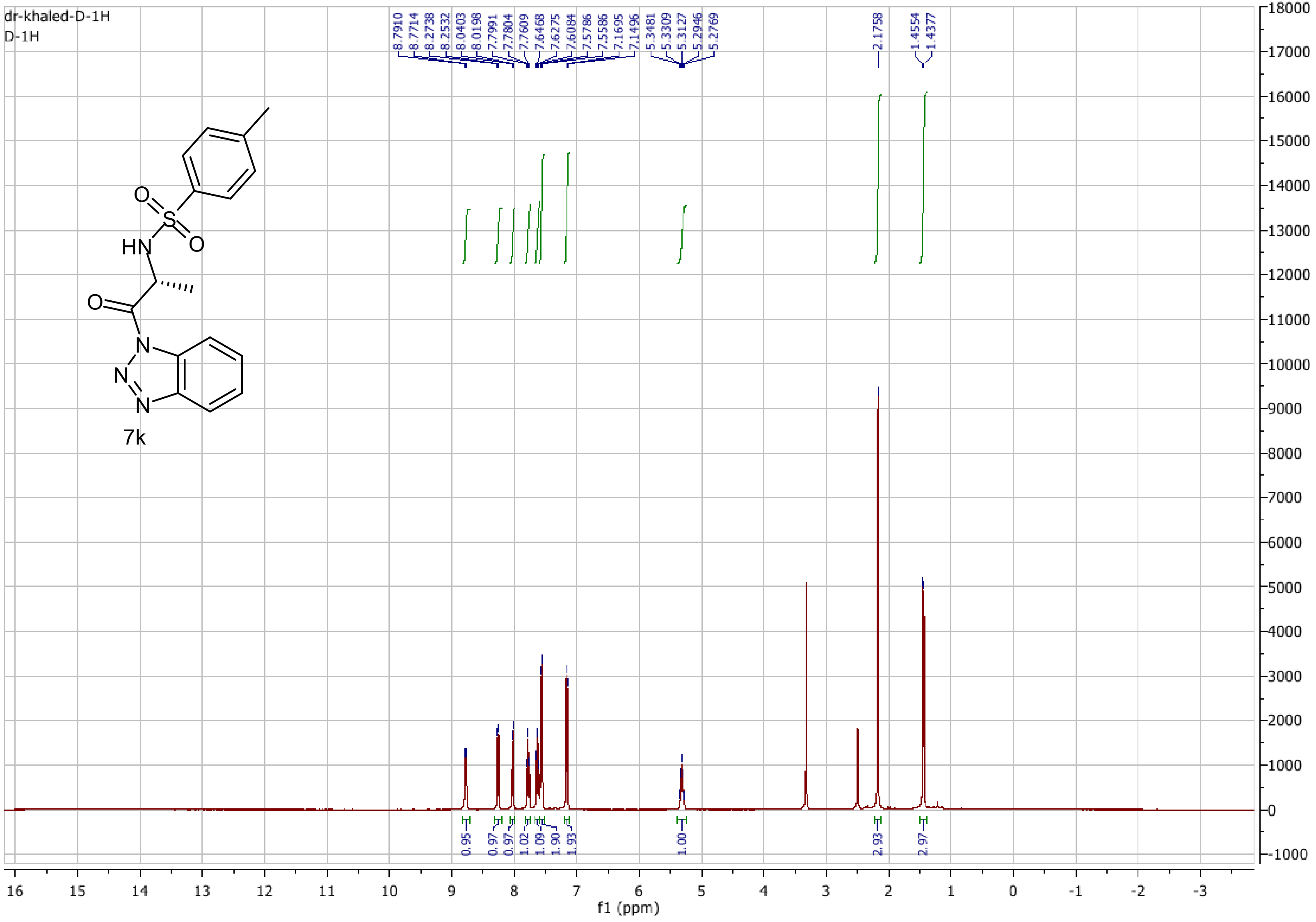
dr-khaled-D-1H
D-1H
7k
f1 (ppm)

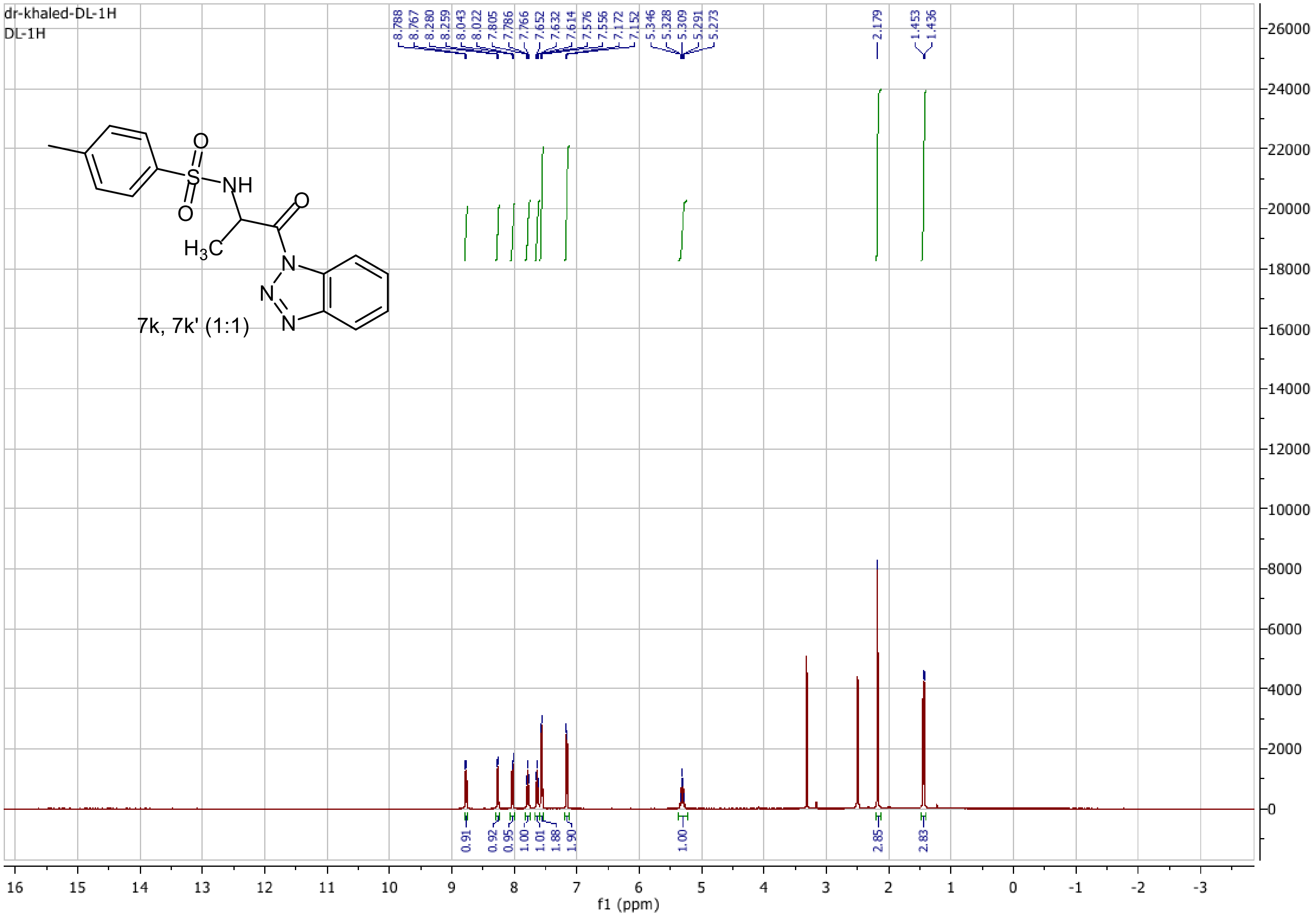
dr-khaled-DL-1H
DL-1H
7k, 7k' (1:1)
8.788
8.767
8.280
8.259
8.043
8.022
7.805
7.786
7.766
7.652
7.632
7.614
7.576
7.556
7.172
7.152
5.346
5.328
5.309
5.291
5.273
2.179
1.453
1.436
0.91
0.92
0.95
1.00
1.01
1.88
1.90
1.00
2.85
2.83
f1 (ppm)

**Figure S2.** $^{1}$H and $^{13}$C NMR spectra of compounds **8a–j**.

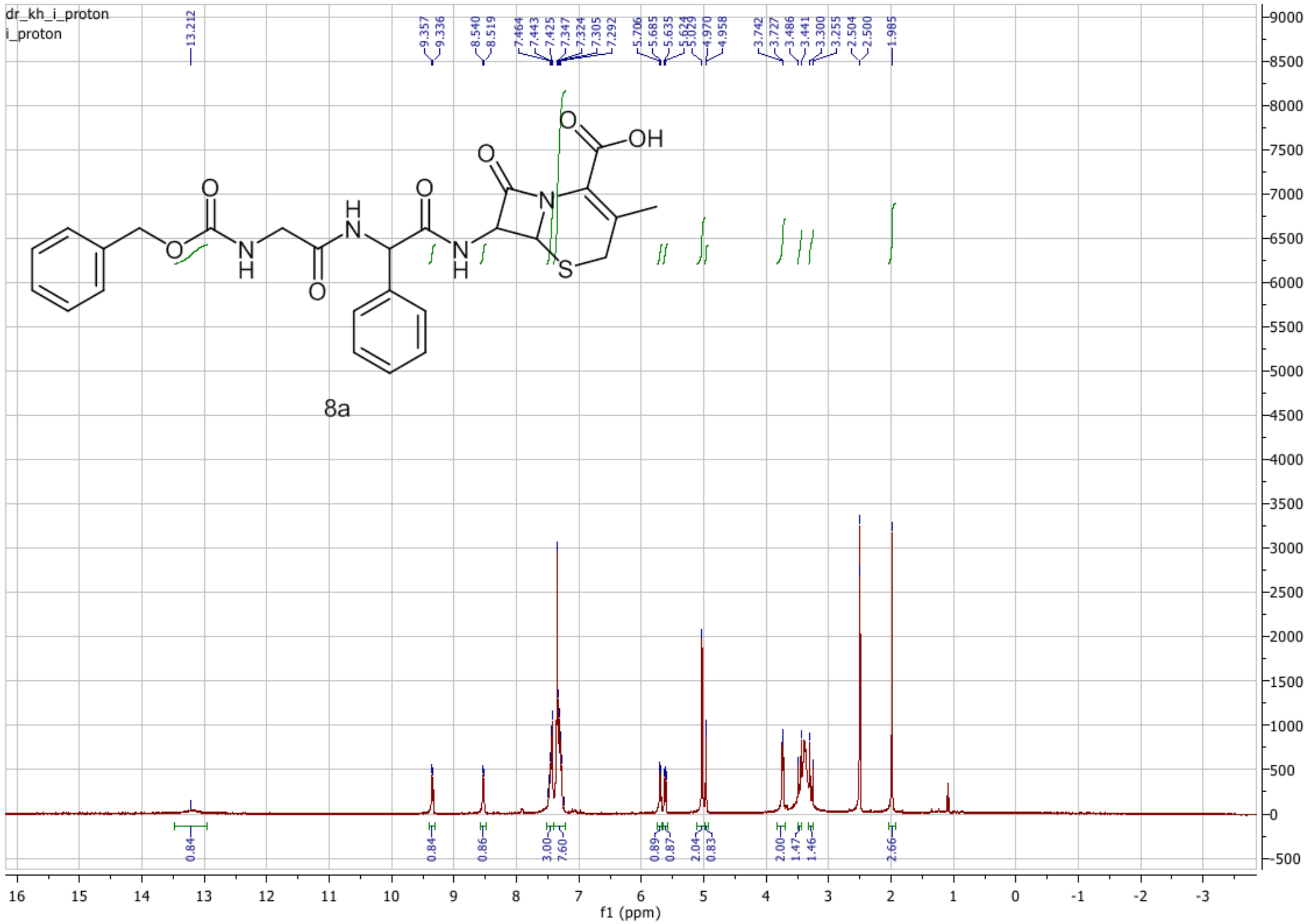

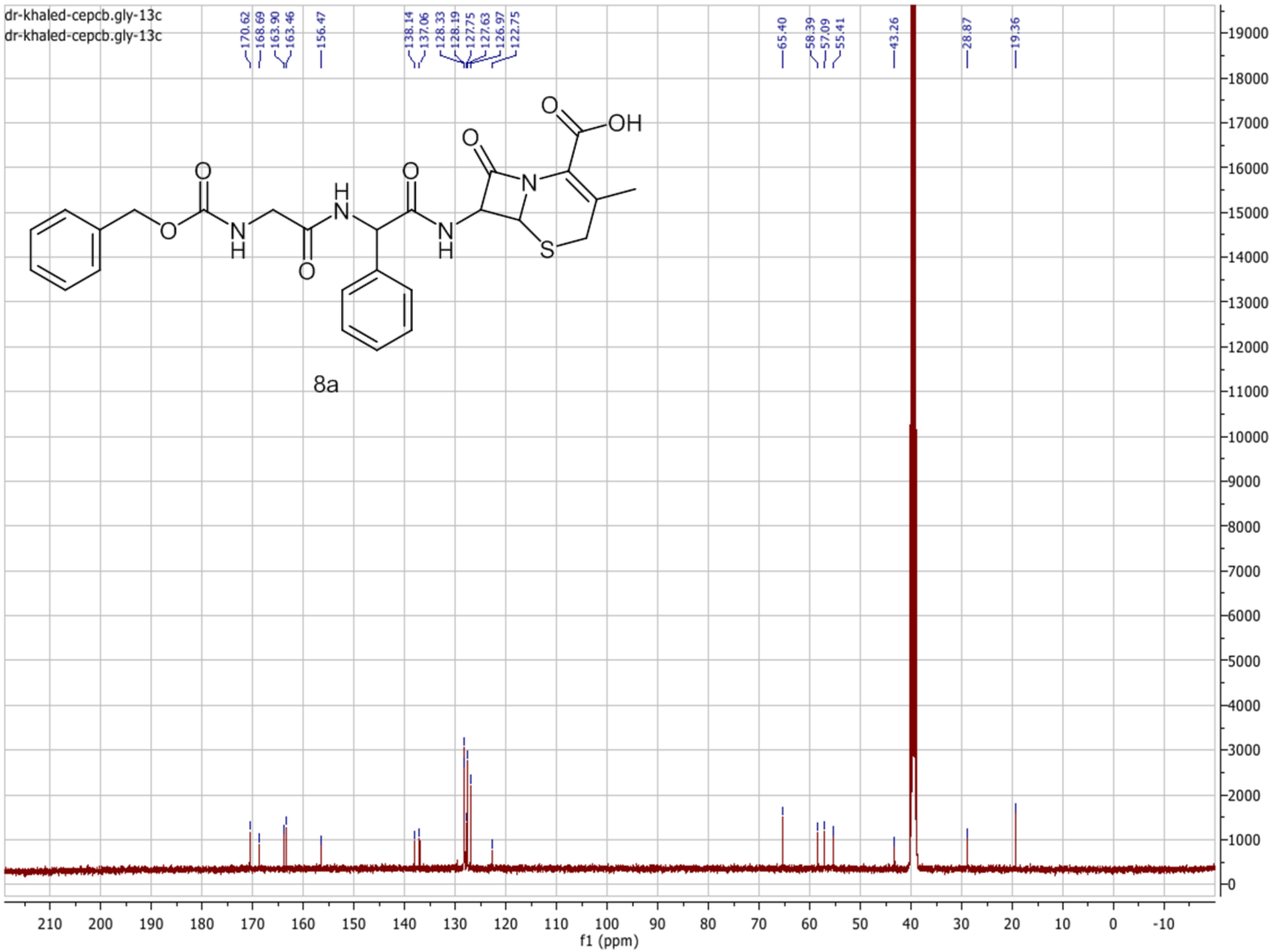
dr-khaled-cepcb.gly-13c
dr-khaled-cepcb.gly-13c
170.62
168.69
163.90
163.46
156.47
138.14
137.06
128.33
128.19
127.75
127.63
126.97
122.75
65.40
58.39
57.09
55.41
43.26
28.87
19.36
8a
f1 (ppm)

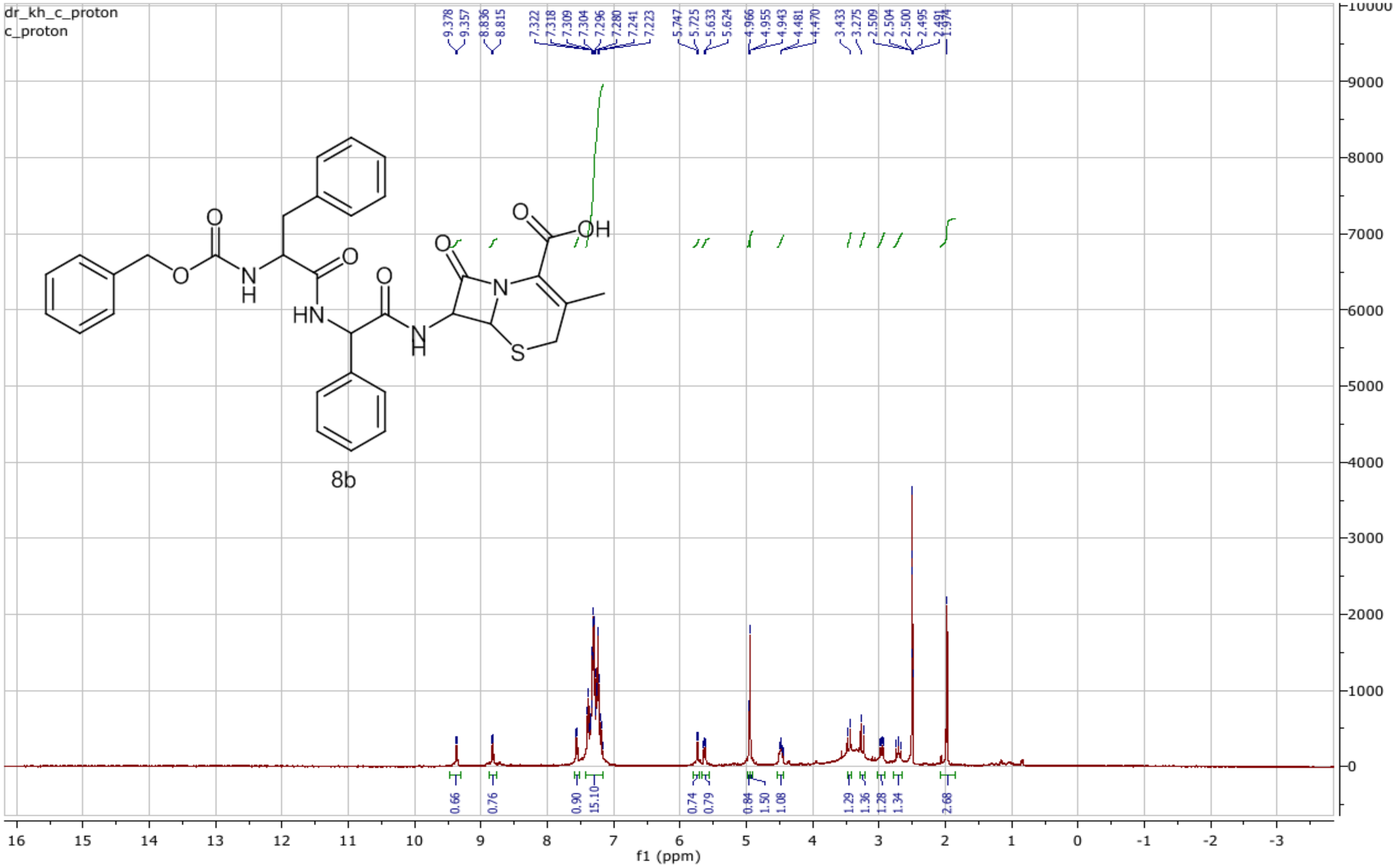
dr_kh_c_proton
c_proton
8b
f1 (ppm)

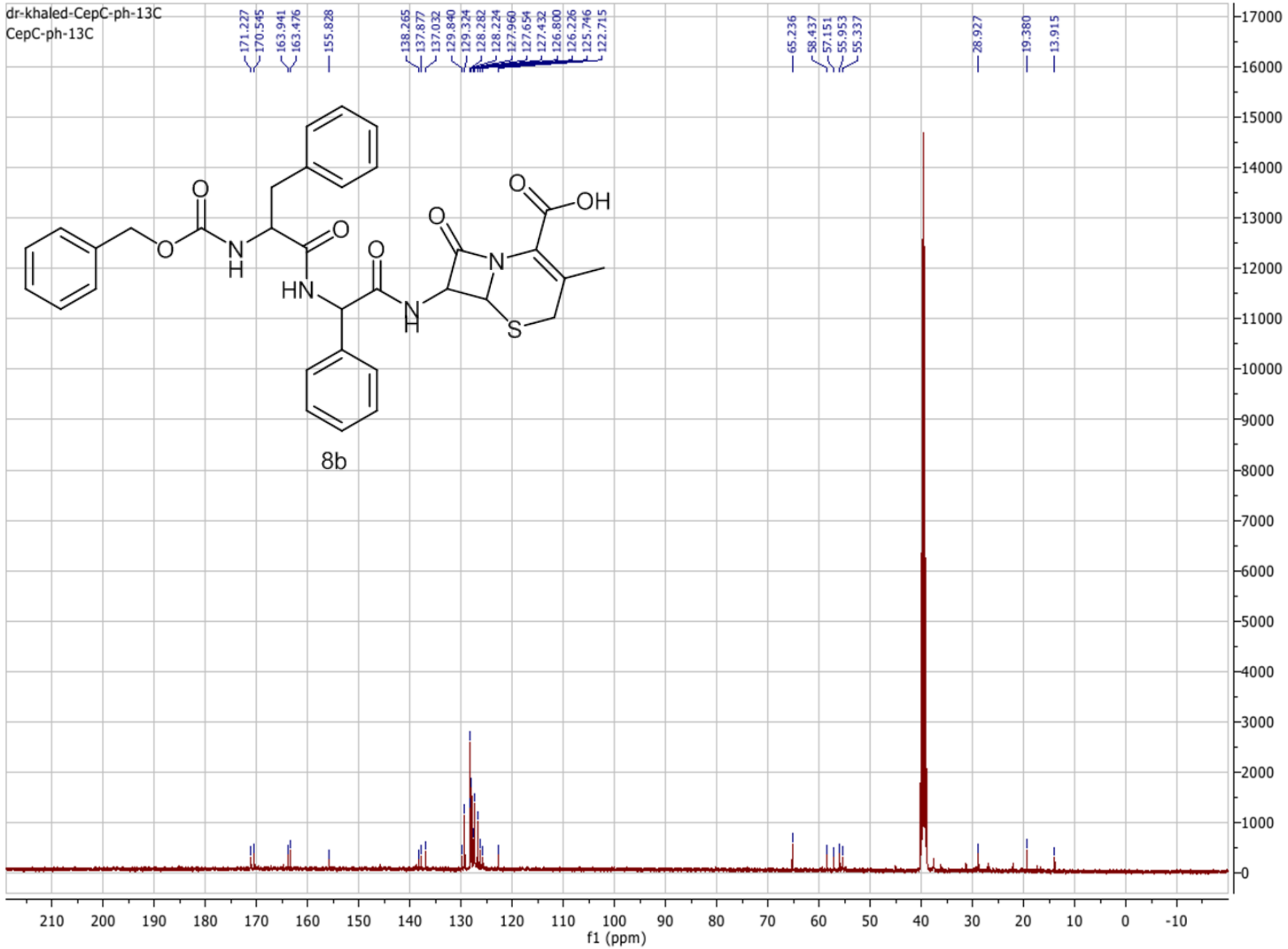
dr-khaled-CepC-ph-13C
CepC-ph-13C
171.227
170.545
163.941
163.476
155.828
138.265
137.877
137.032
129.840
129.324
128.282
128.224
127.960
127.654
127.432
126.800
126.226
125.746
122.715
65.236
58.437
57.151
55.953
55.337
28.927
19.380
13.915
8b
f1 (ppm)

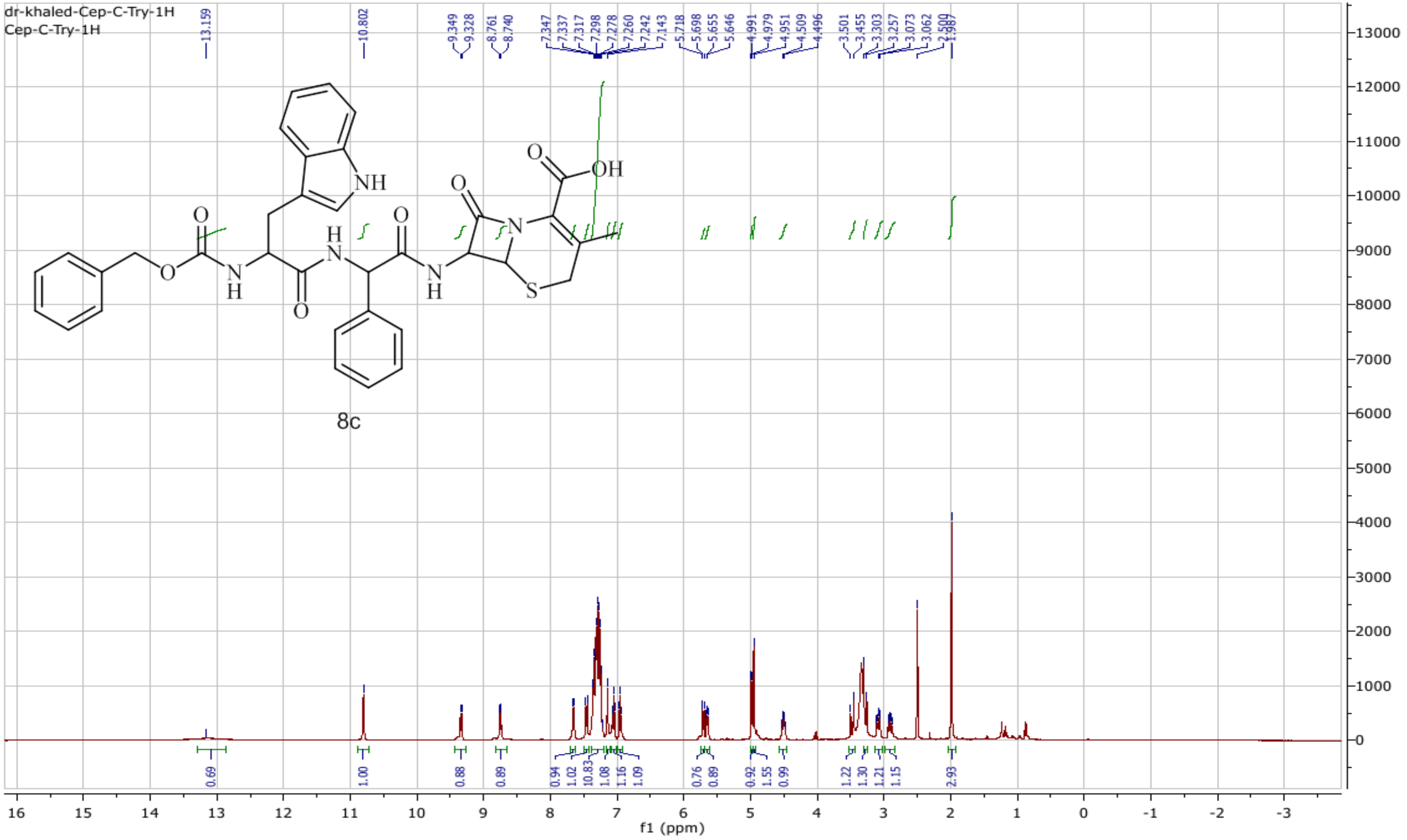

dr-khaled-Cep-C-Try-1H
Cep-C-Try-1H
8c
f1 (ppm)

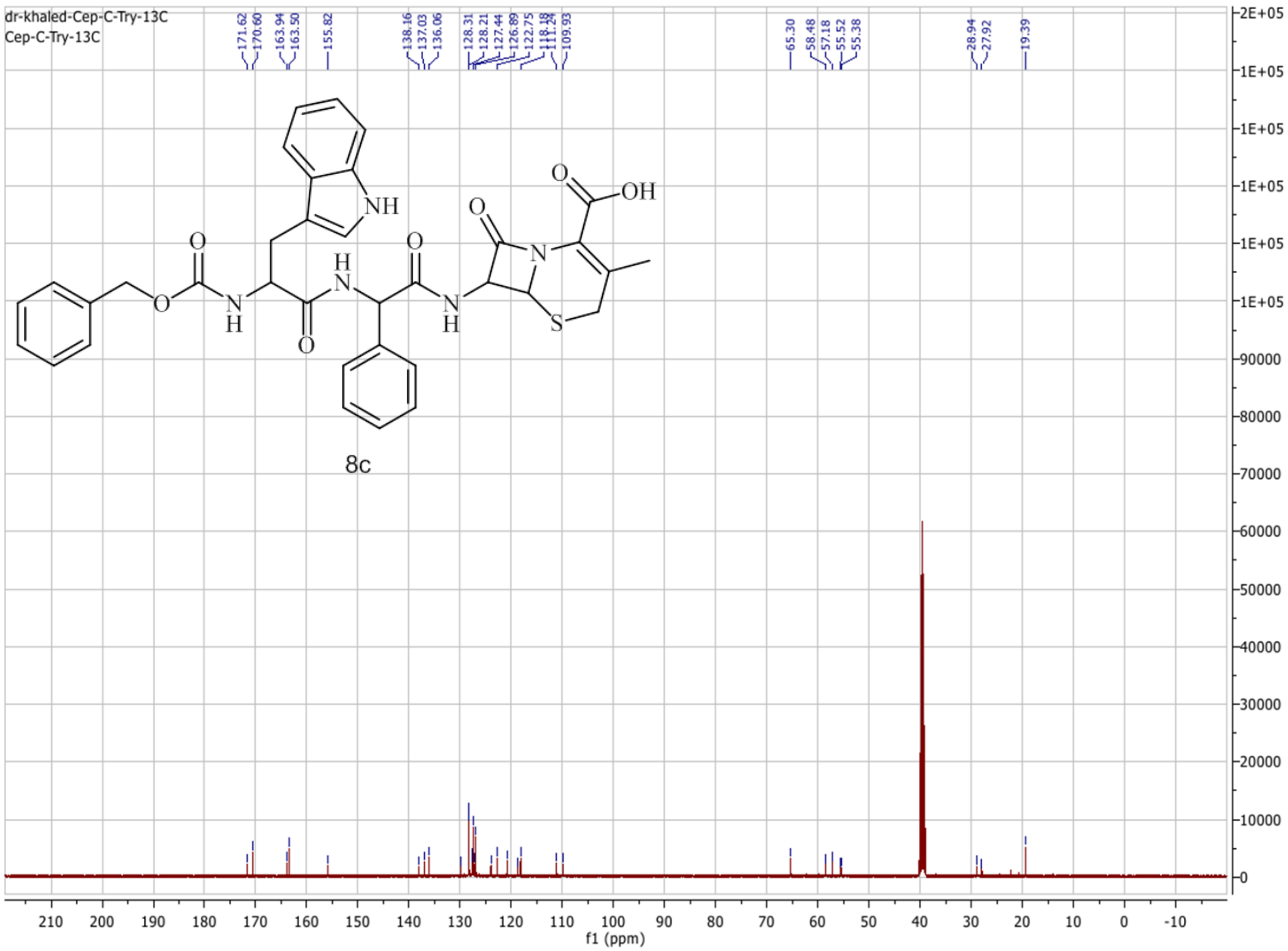
dr-khaled-Cep-C-Try-13C
Cep-C-Try-13C
171.62
170.60
163.94
163.50
155.82
138.16
137.03
136.06
128.31
128.21
127.44
126.89
122.75
109.93
65.30
58.48
57.18
55.52
55.38
28.94
27.92
19.39
NH
O
OH
N
S
H
8c
f1 (ppm)

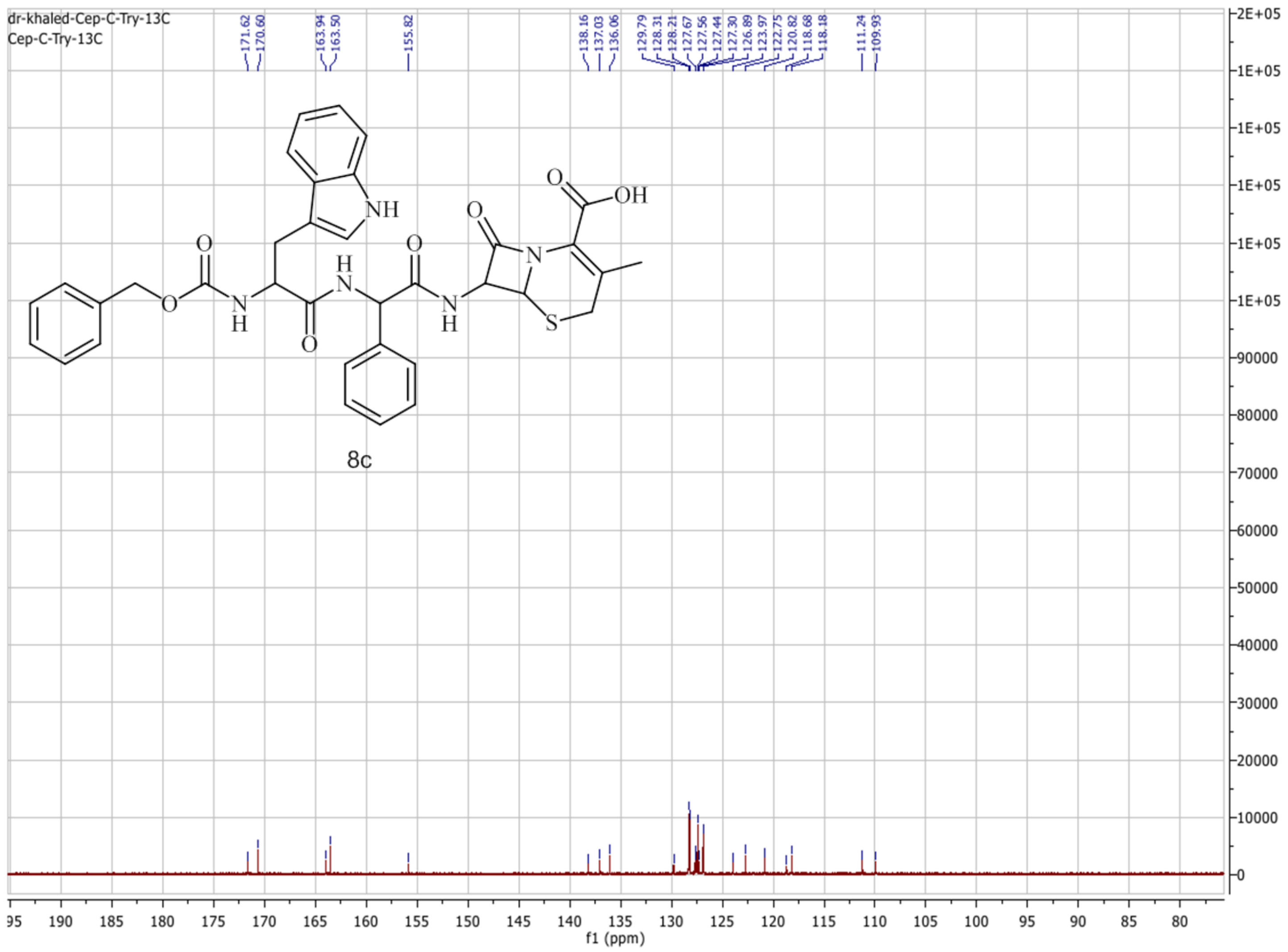
dr-khaled-Cep-C-Try-13C
Cep-C-Try-13C
171.62
170.60
163.94
163.50
155.82
138.16
137.03
136.06
129.79
128.31
128.21
127.67
127.56
127.44
127.30
126.89
123.97
122.75
120.82
118.68
118.18
111.24
109.93
O
NH
O
OH
O
O
H
N
N
O
N
H
O
N
H
S
O
8c
f1 (ppm)

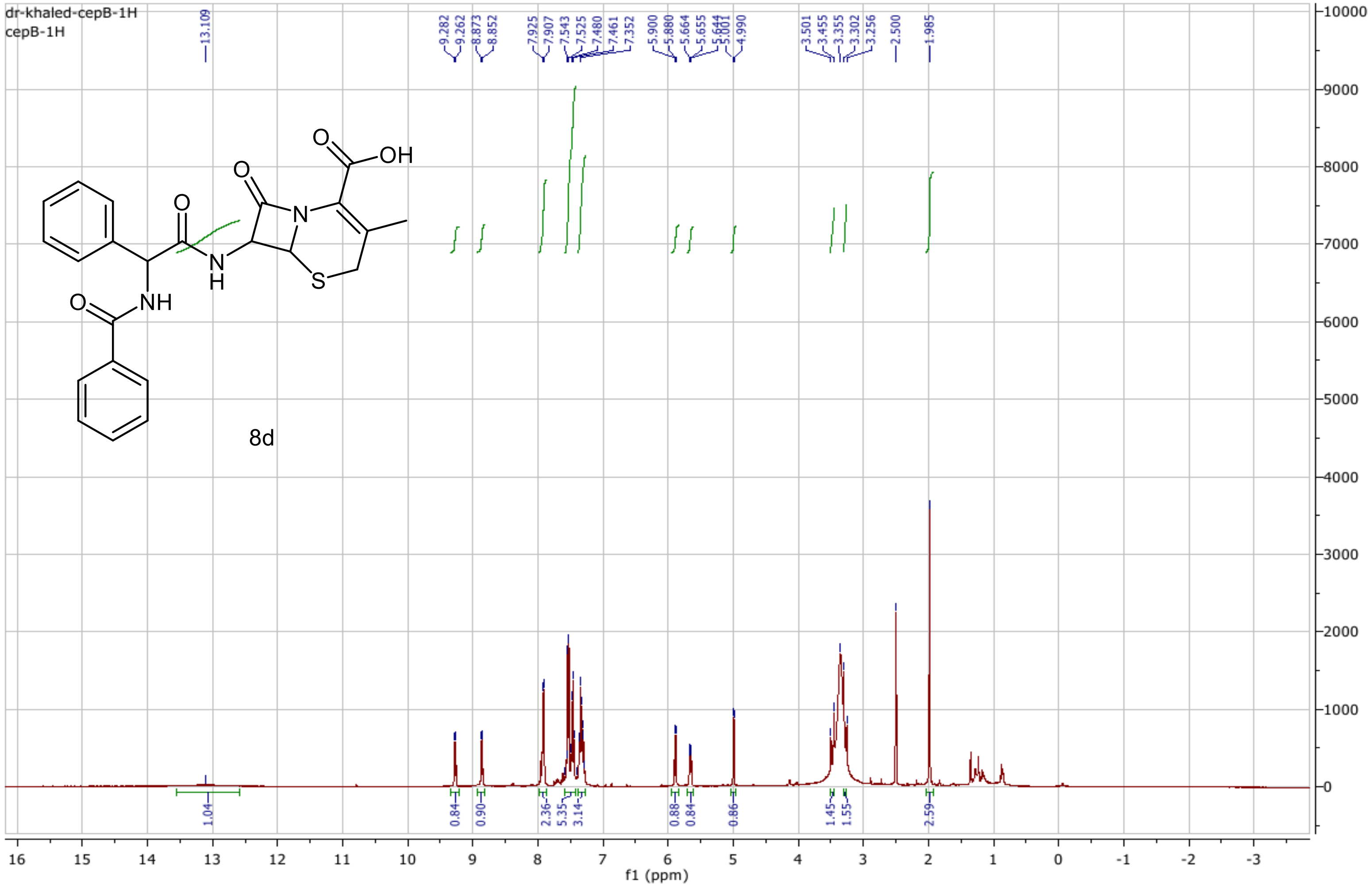
dr-khaled-cepB-1H
cepB-1H
8d
f1 (ppm)

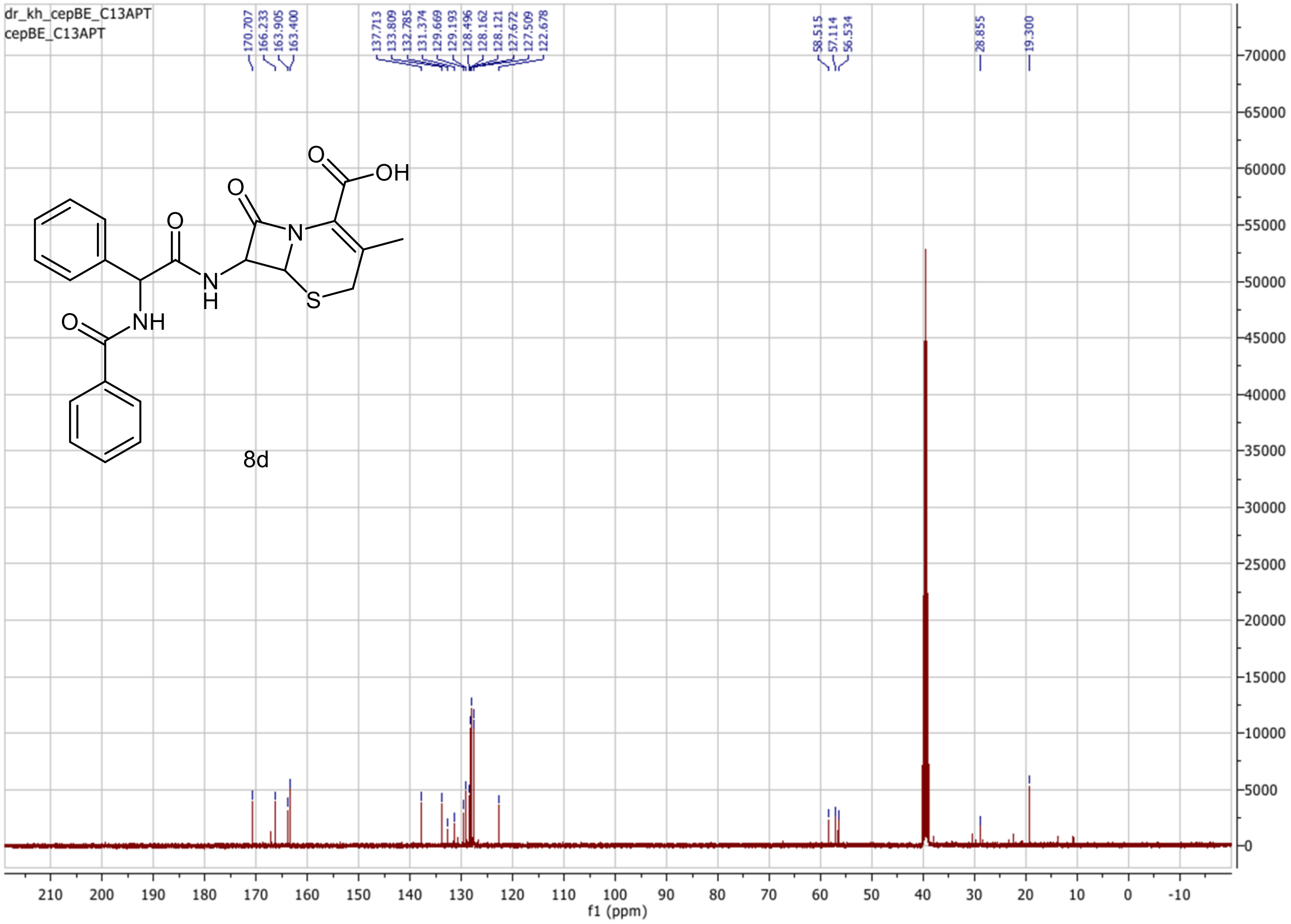
dr_kh_cepBE_C13APT
cepBE_C13APT
170.707
166.233
163.905
163.400
137.713
133.809
132.785
131.374
129.669
129.193
128.496
128.162
128.121
127.672
127.509
122.678
58.515
57.114
56.534
28.855
19.300
8d
f1 (ppm)

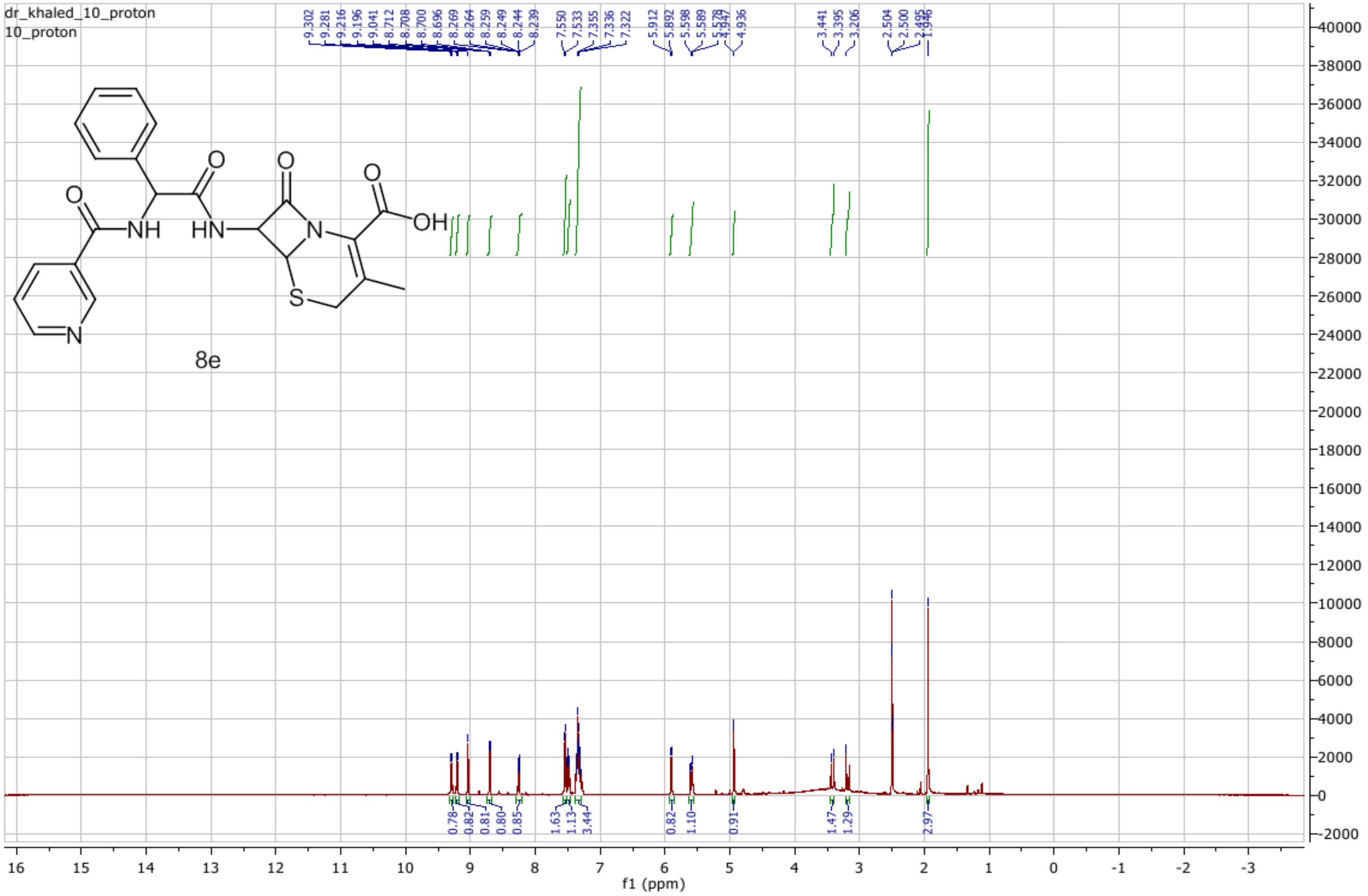
dr_khaled_10_proton
10_proton
8e
f1 (ppm)

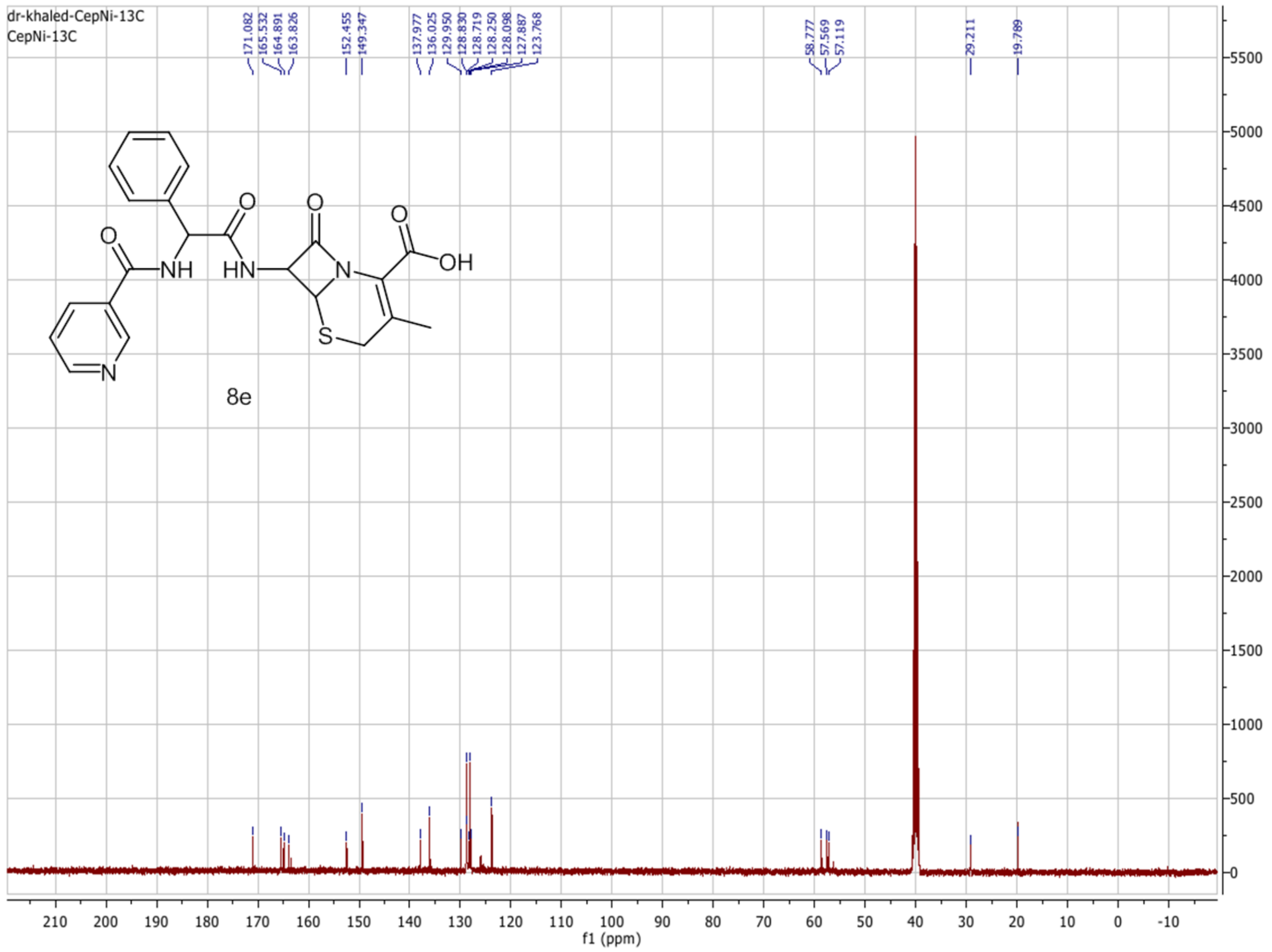
dr-khaled-CepNi-13C
CepNi-13C
171.082
165.532
164.891
163.826
152.455
149.347
137.977
136.025
129.950
128.830
128.719
128.250
128.098
127.887
123.768
58.777
57.569
57.119
29.211
19.789
8e
O
NH
HN
N
S
OH
f1 (ppm)

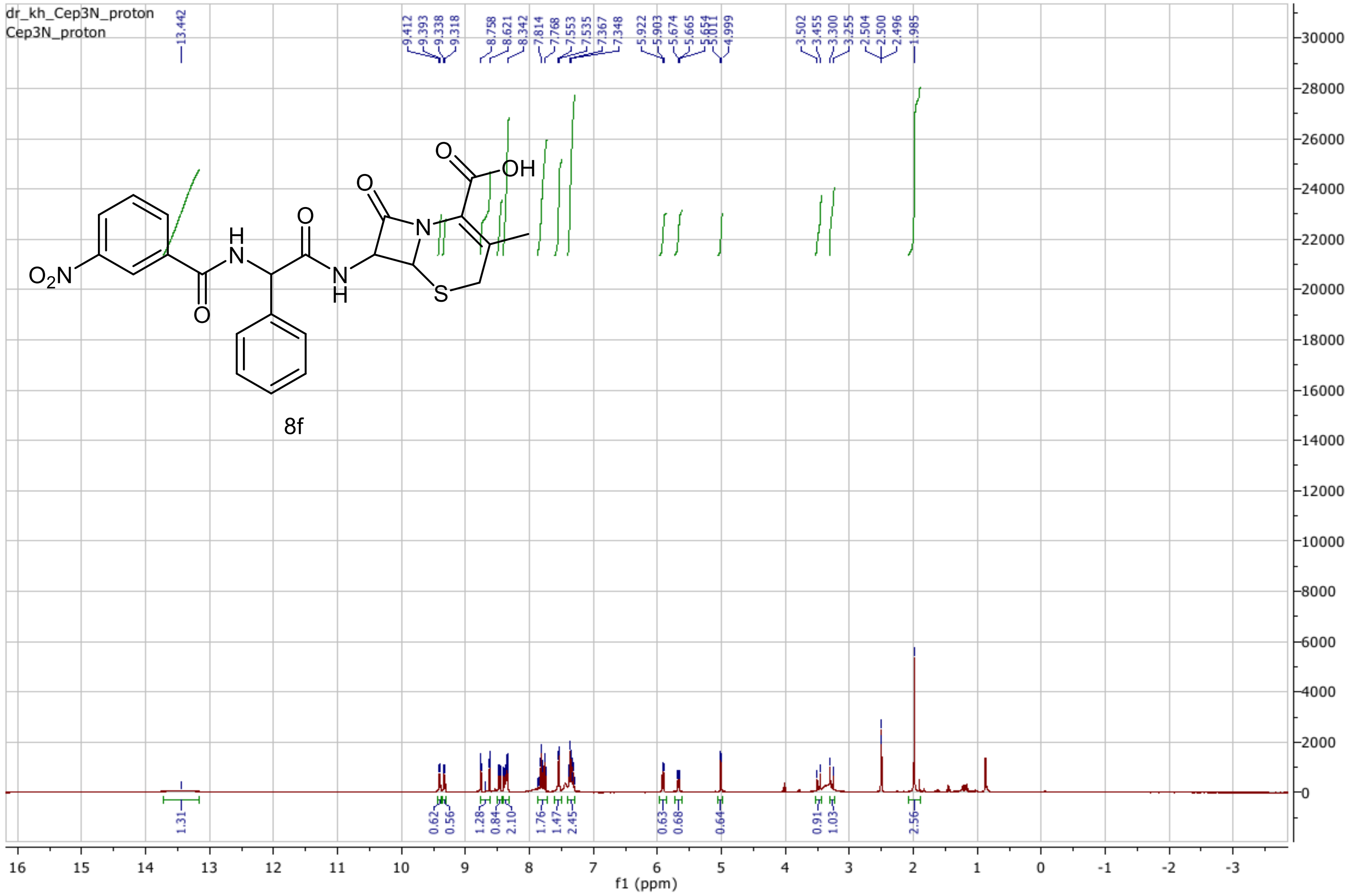
dr_kh_Cep3N_proton
Cep3N_proton
8f
f1 (ppm)

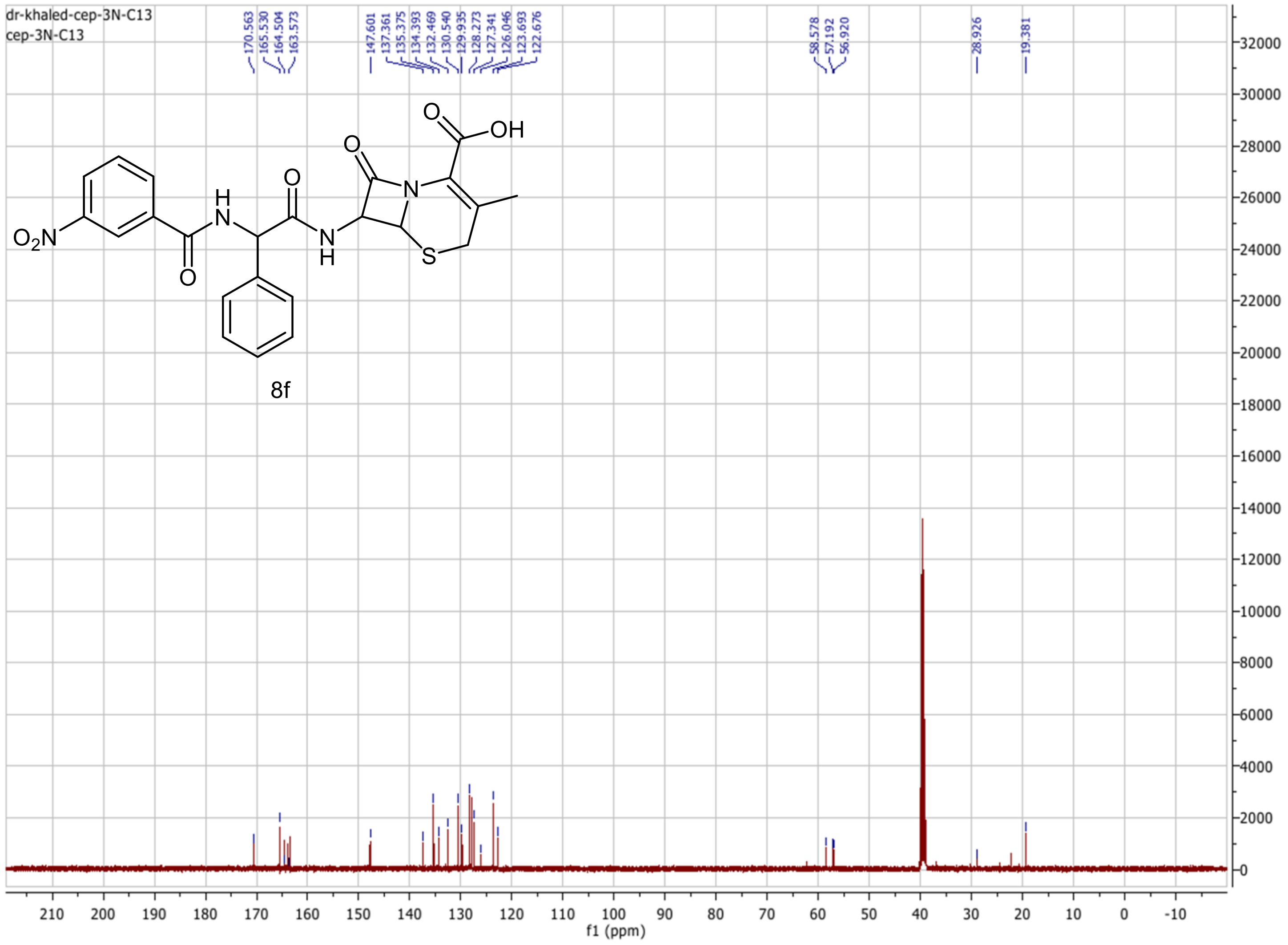
dr-khaled-cep-3N-C13
cep-3N-C13
170.563
165.530
164.504
163.573
147.601
137.361
135.375
134.393
132.469
130.540
129.935
128.273
127.341
126.046
123.693
122.676
58.578
57.192
56.920
28.926
19.381
O₂N
8f
f1 (ppm)

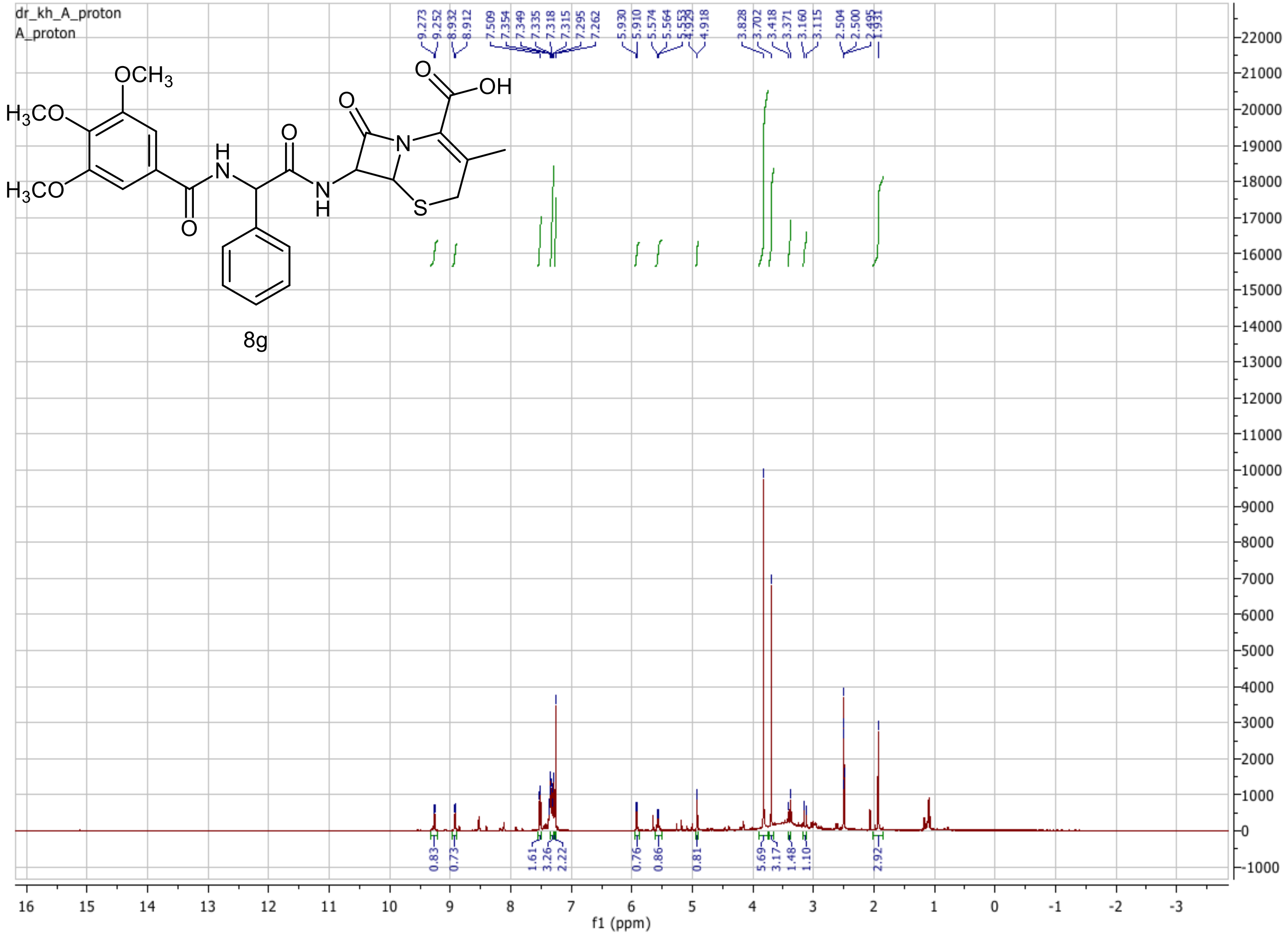
dr_kh_A_proton
A_proton
OCH3
H3CO
H3CO
H
N
O
O
N
H
O
N
S
O
OH
8g
9.273
9.252
8.932
8.912
7.509
7.354
7.349
7.335
7.318
7.315
7.295
7.262
5.930
5.910
5.574
5.564
5.553
4.929
4.918
3.828
3.702
3.418
3.371
3.160
3.115
2.504
2.500
2.495
1.931
0.83
0.73
1.61
3.26
2.22
0.76
0.86
0.81
5.69
3.17
1.48
1.10
2.92
f1 (ppm)

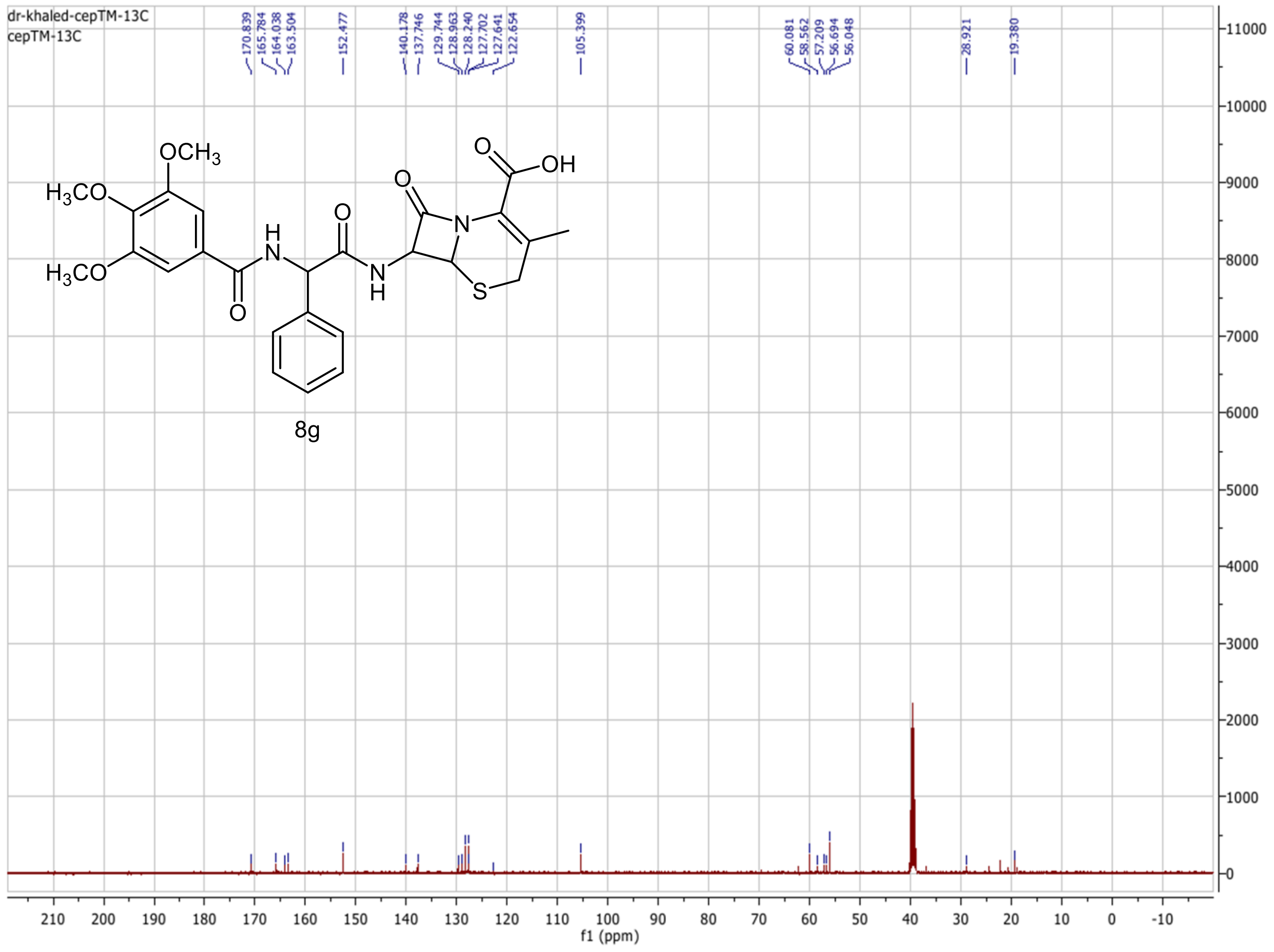
dr-khaled-cepTM-13C
cepTM-13C
170.839
165.784
164.038
163.504
152.477
140.178
137.746
129.744
128.963
128.240
127.702
127.641
122.654
105.399
60.081
58.562
57.209
56.694
56.048
28.921
19.380
8g
f1 (ppm)

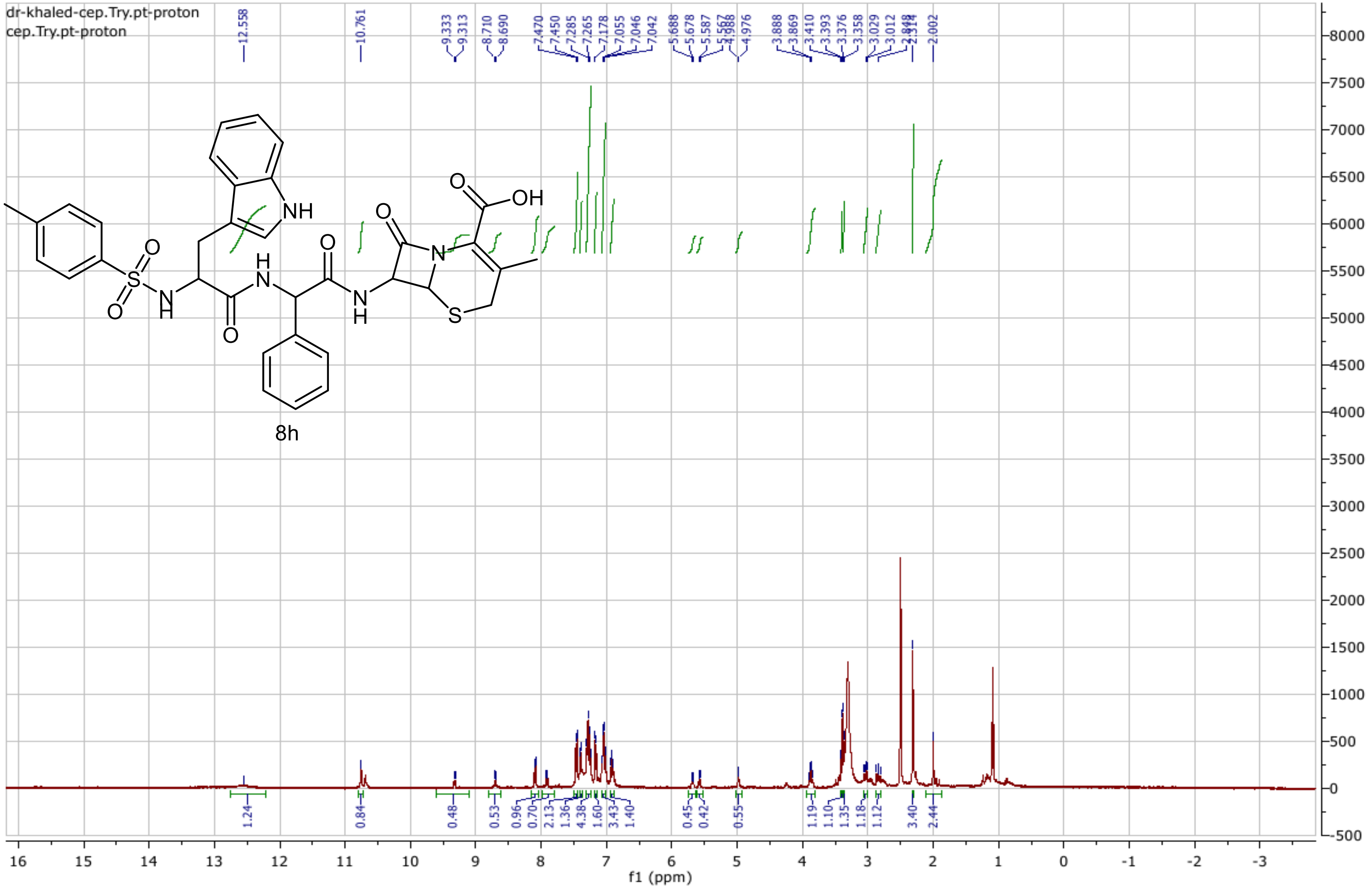
dr-khaled-cep.Try.pt-proton
cep.Try.pt-proton
8h
f1 (ppm)

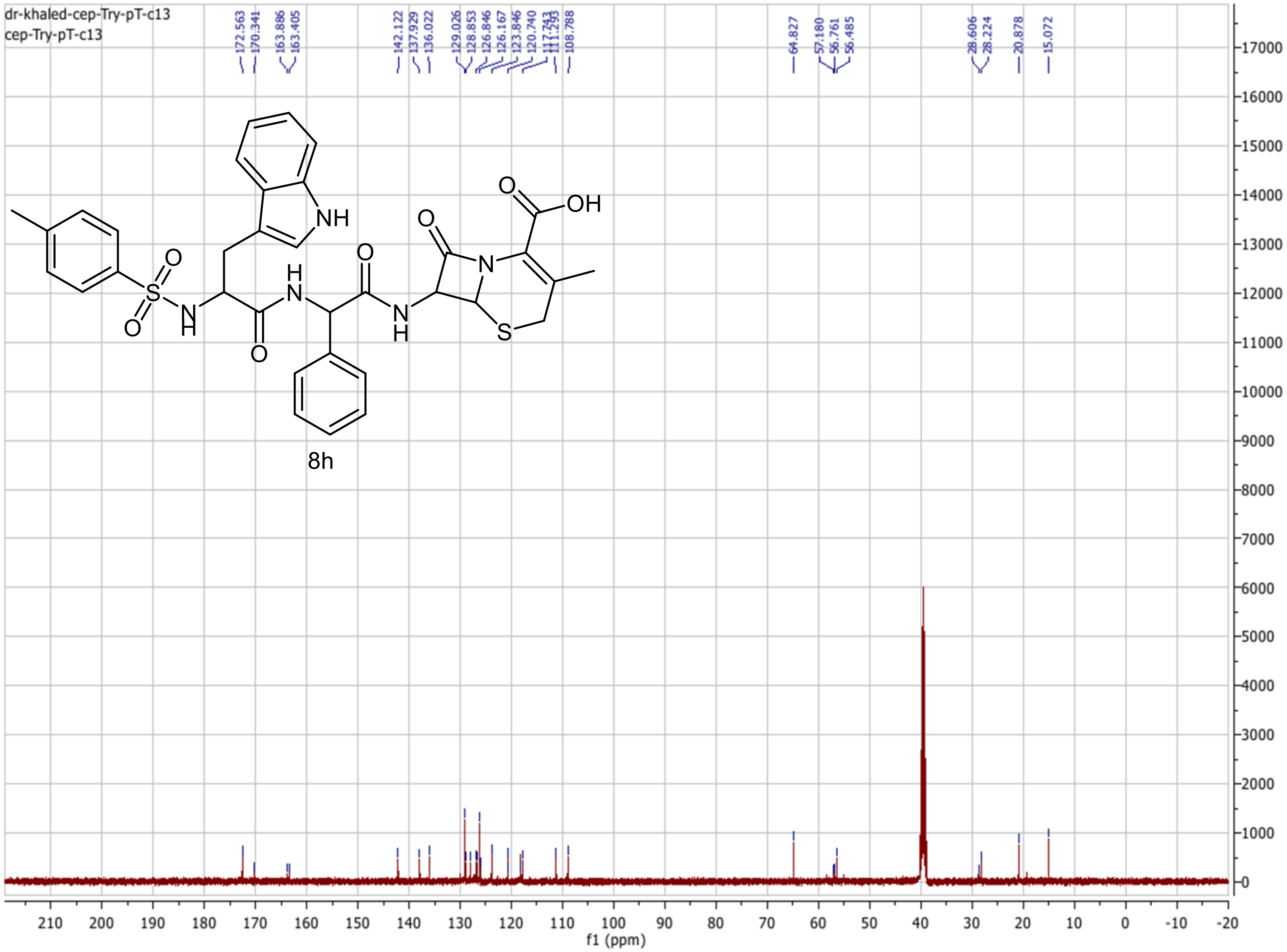
dr-khaled-cep-Try-pT-c13
cep-Try-pT-c13
172.563
170.341
163.886
163.405
142.122
137.929
136.022
129.026
128.853
126.846
126.167
123.846
120.740
117.743
111.293
108.788
64.827
57.180
56.761
56.485
28.606
28.224
20.878
15.072
8h
f1 (ppm)

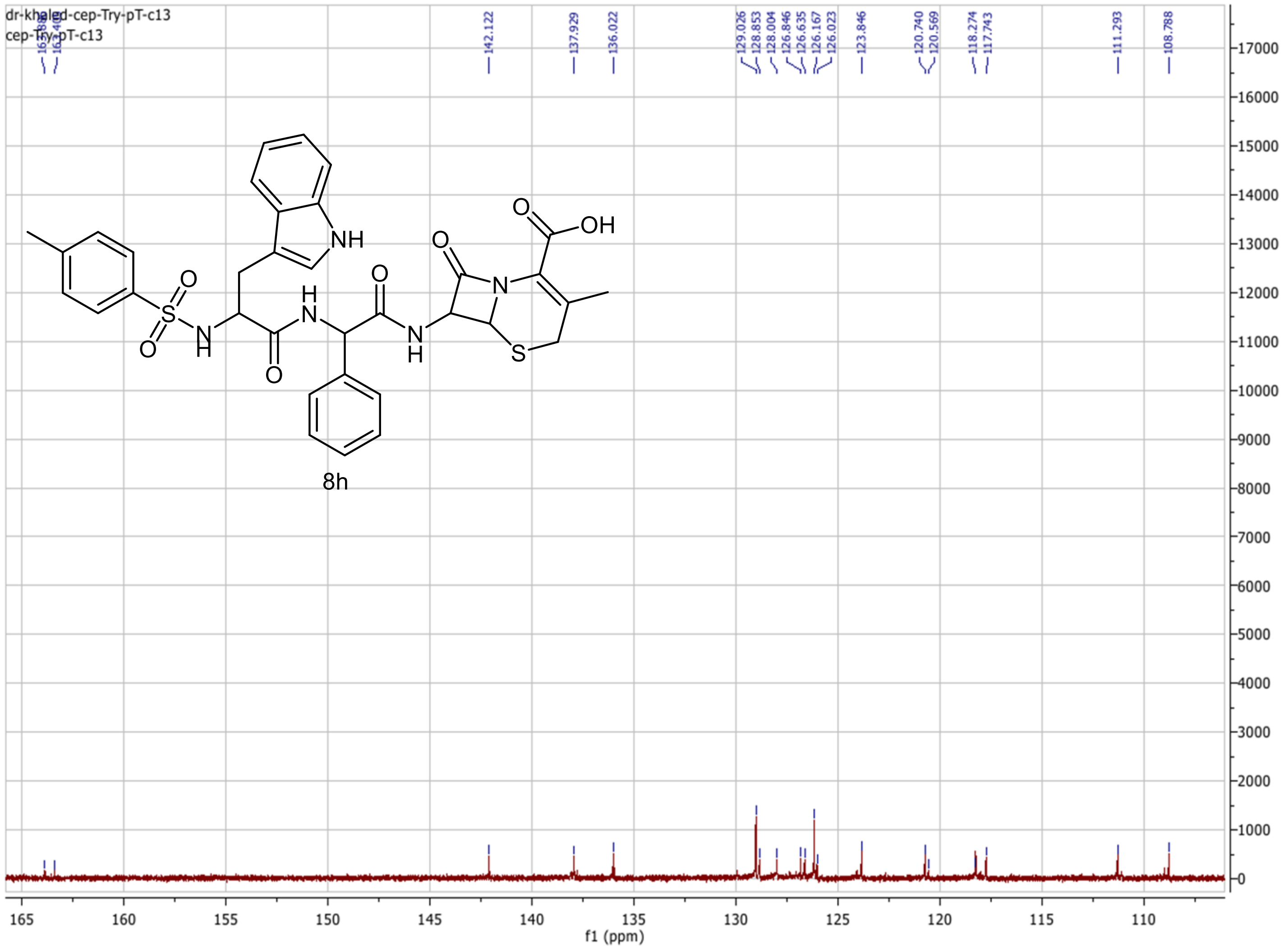

dr-khaled-cep-Try-pT-c13
cep-Try-pT-c13
142.122
137.929
136.022
129.026
128.853
128.004
126.846
126.635
126.167
126.023
123.846
120.740
120.569
118.274
117.743
111.293
108.788
8h
f1 (ppm)

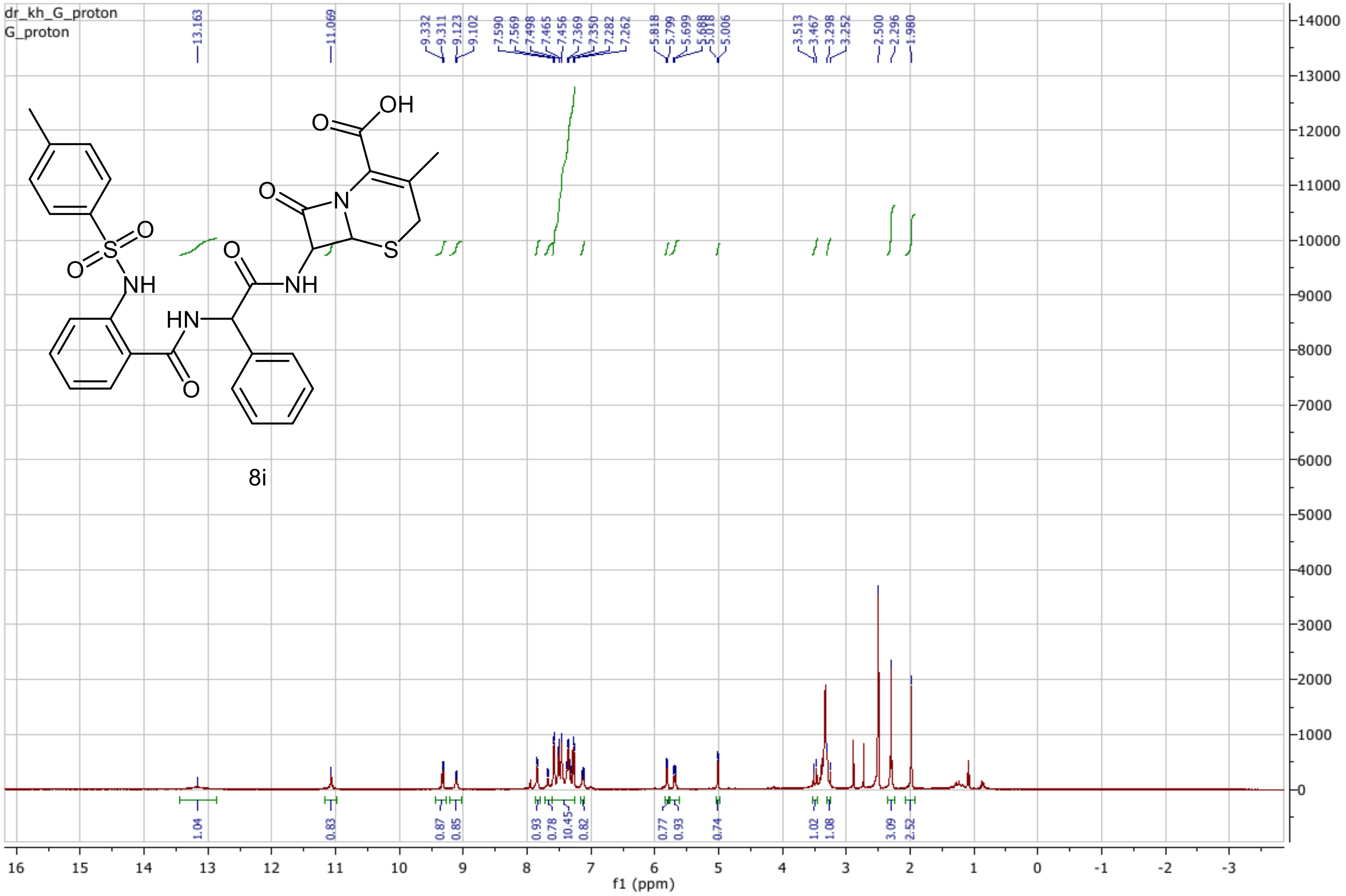
dr_kh_G_proton
G_proton
8i
f1 (ppm)

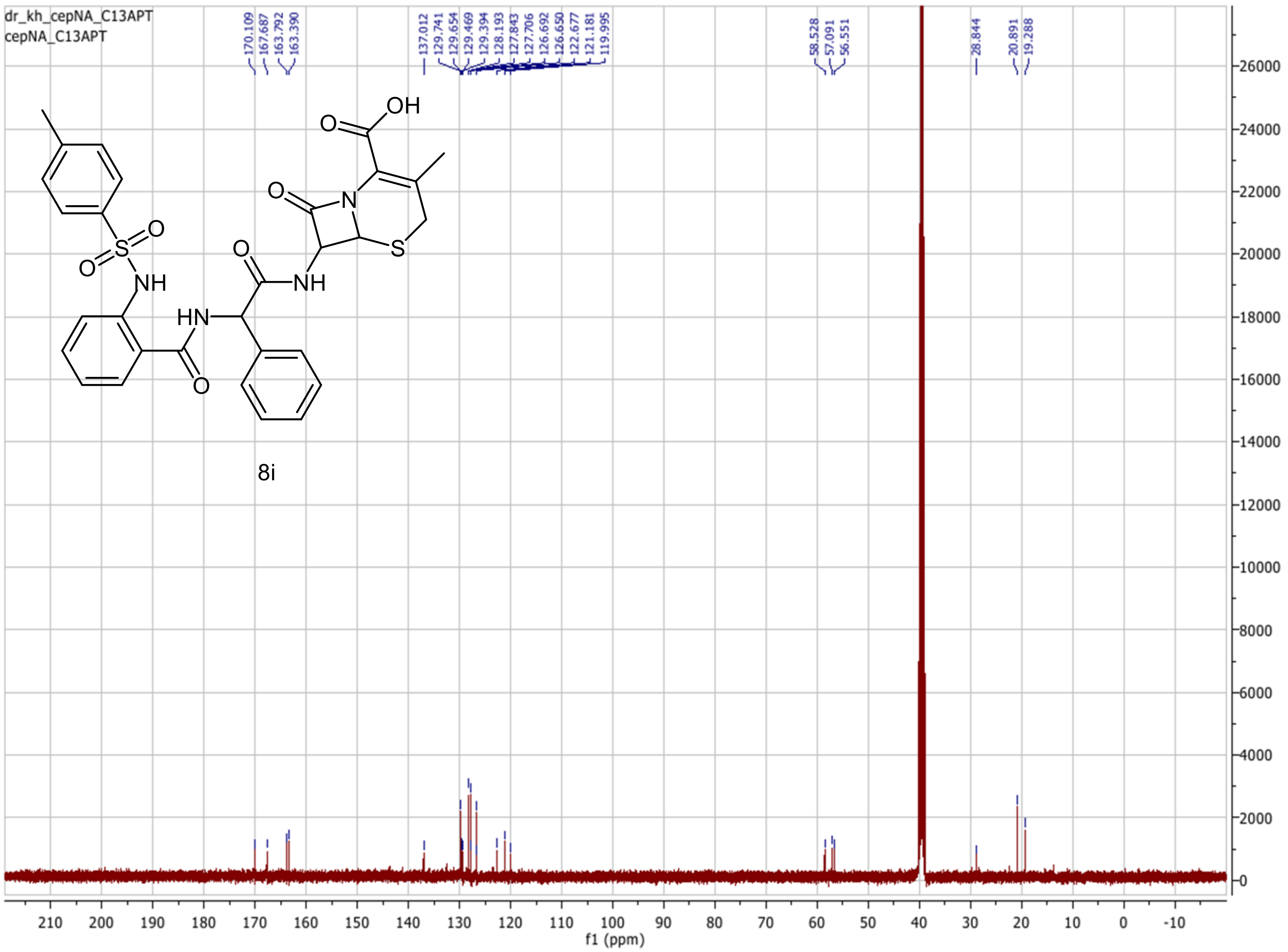

dr_kh_cepNA_C13APT
cepNA_C13APT
170.109
167.687
163.792
163.390
137.012
129.741
129.654
129.469
129.394
128.193
127.843
127.706
126.692
126.650
122.677
121.181
119.995
58.528
57.091
56.551
28.844
20.891
19.288
8i
f1 (ppm)

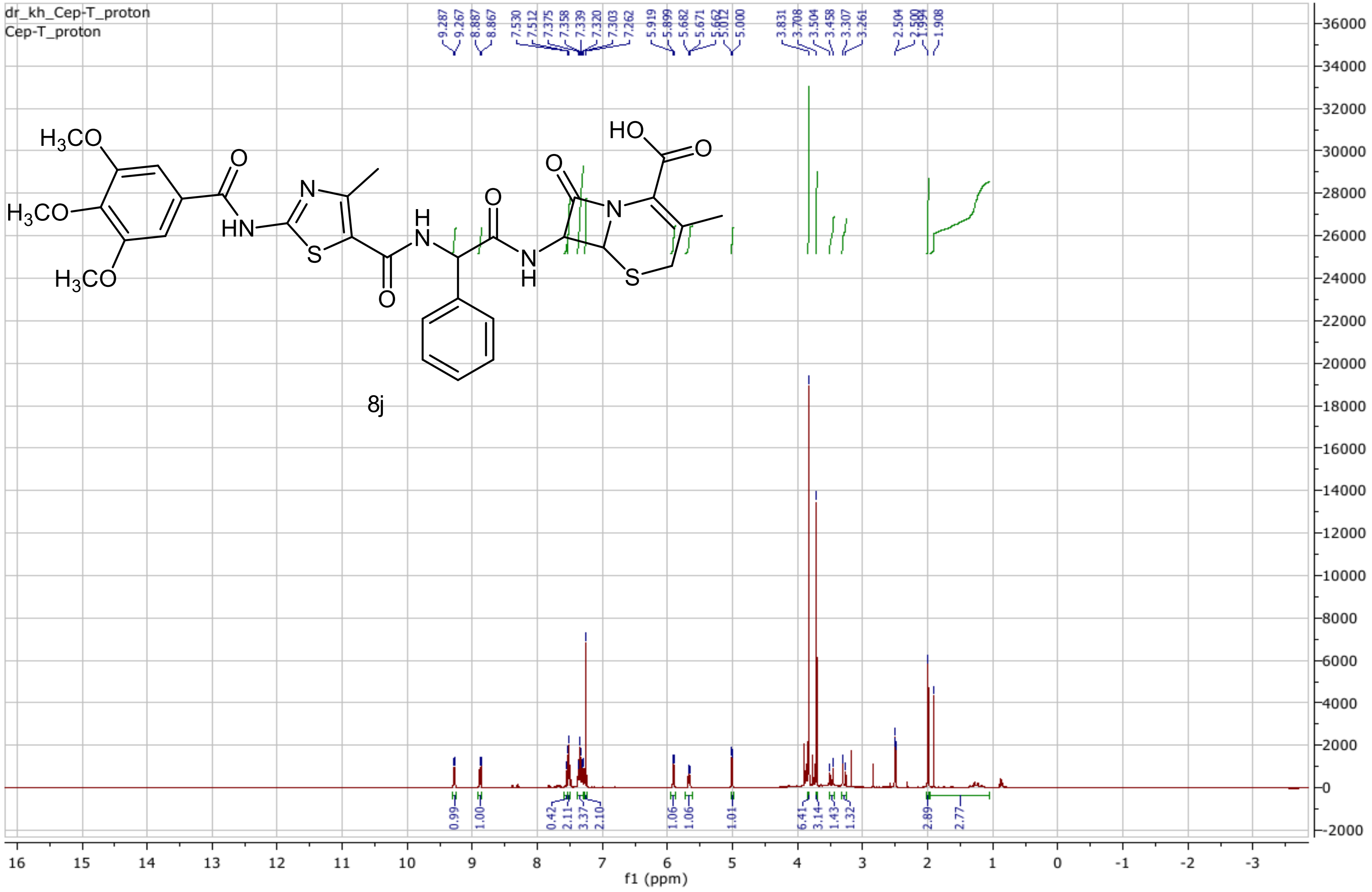
dr_kh_Cep-T_proton
Cep-T_proton
8j
f1 (ppm)

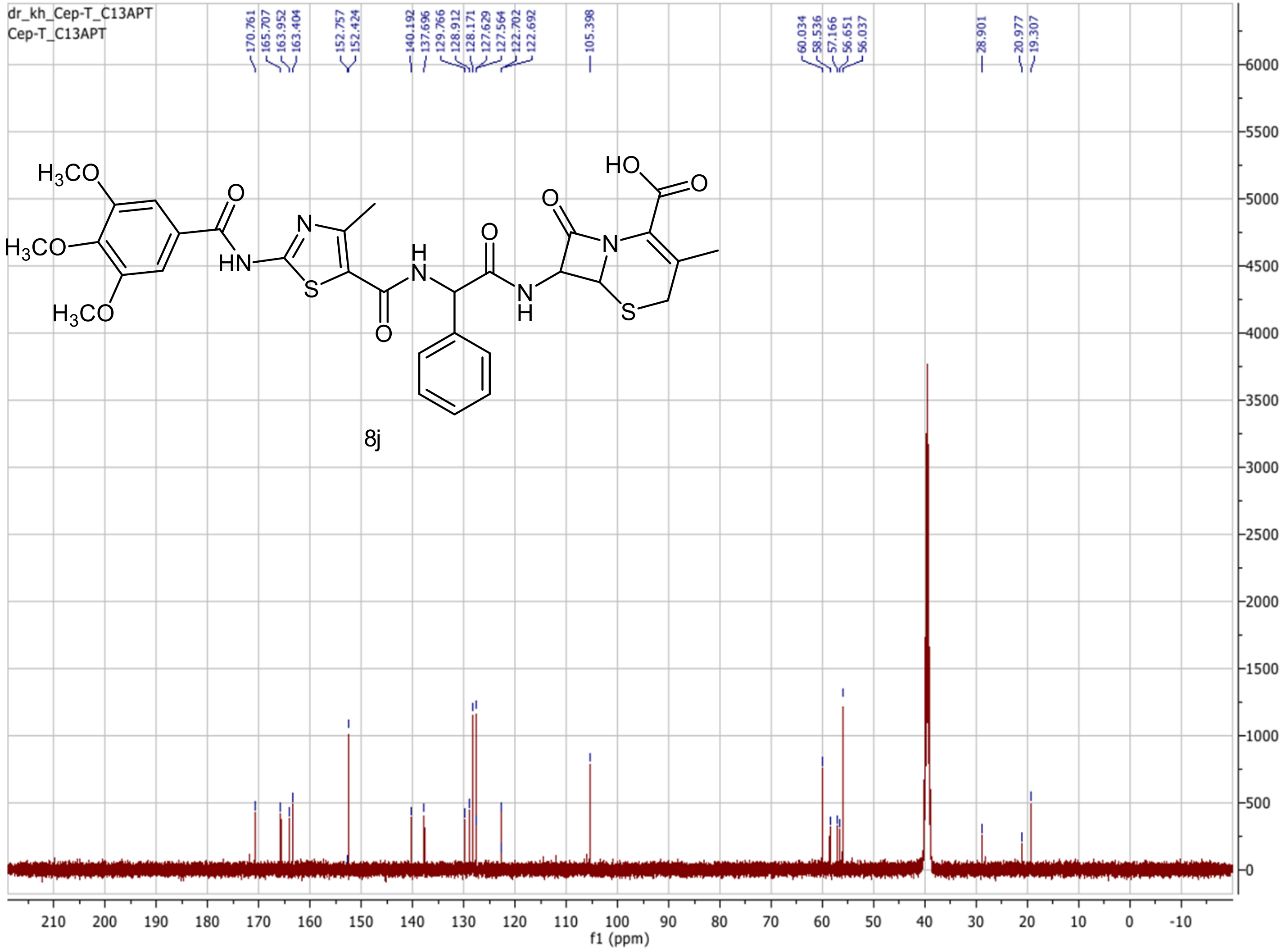
dr_kh_Cep-T_C13APT
Cep-T_C13APT
8j
f1 (ppm)

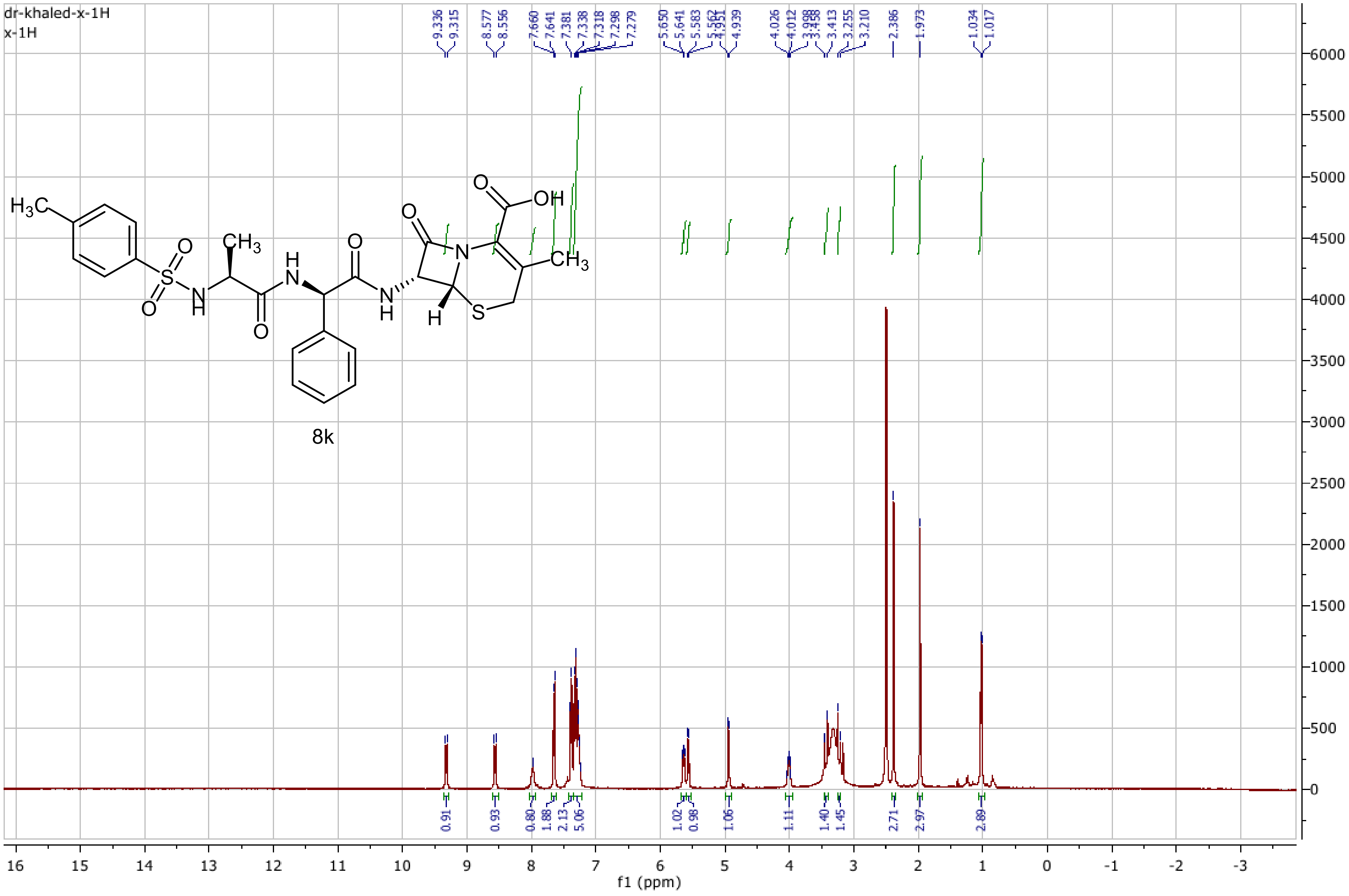
dr-khaled-x-1H
x-1H
8k
f1 (ppm)

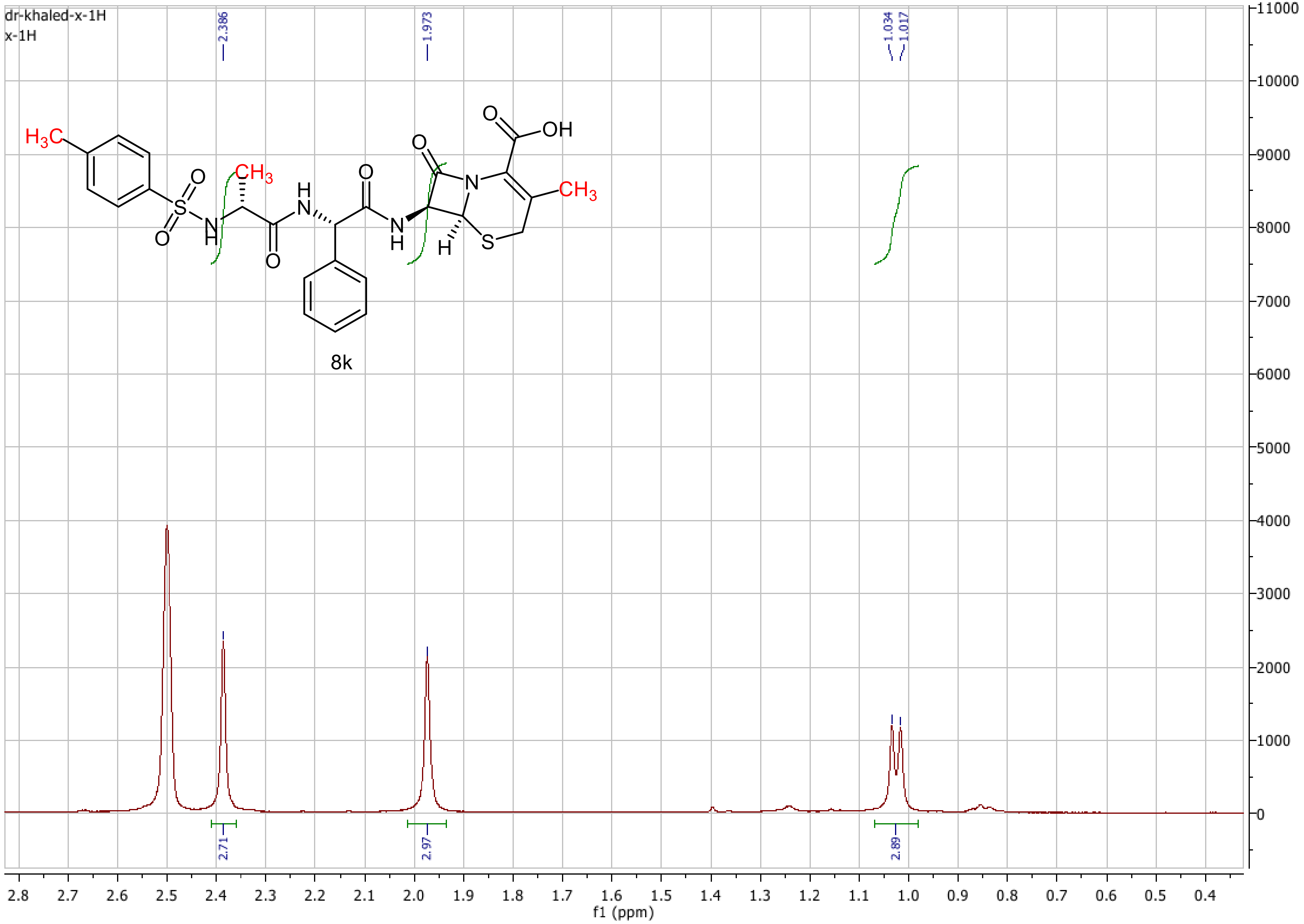


8k

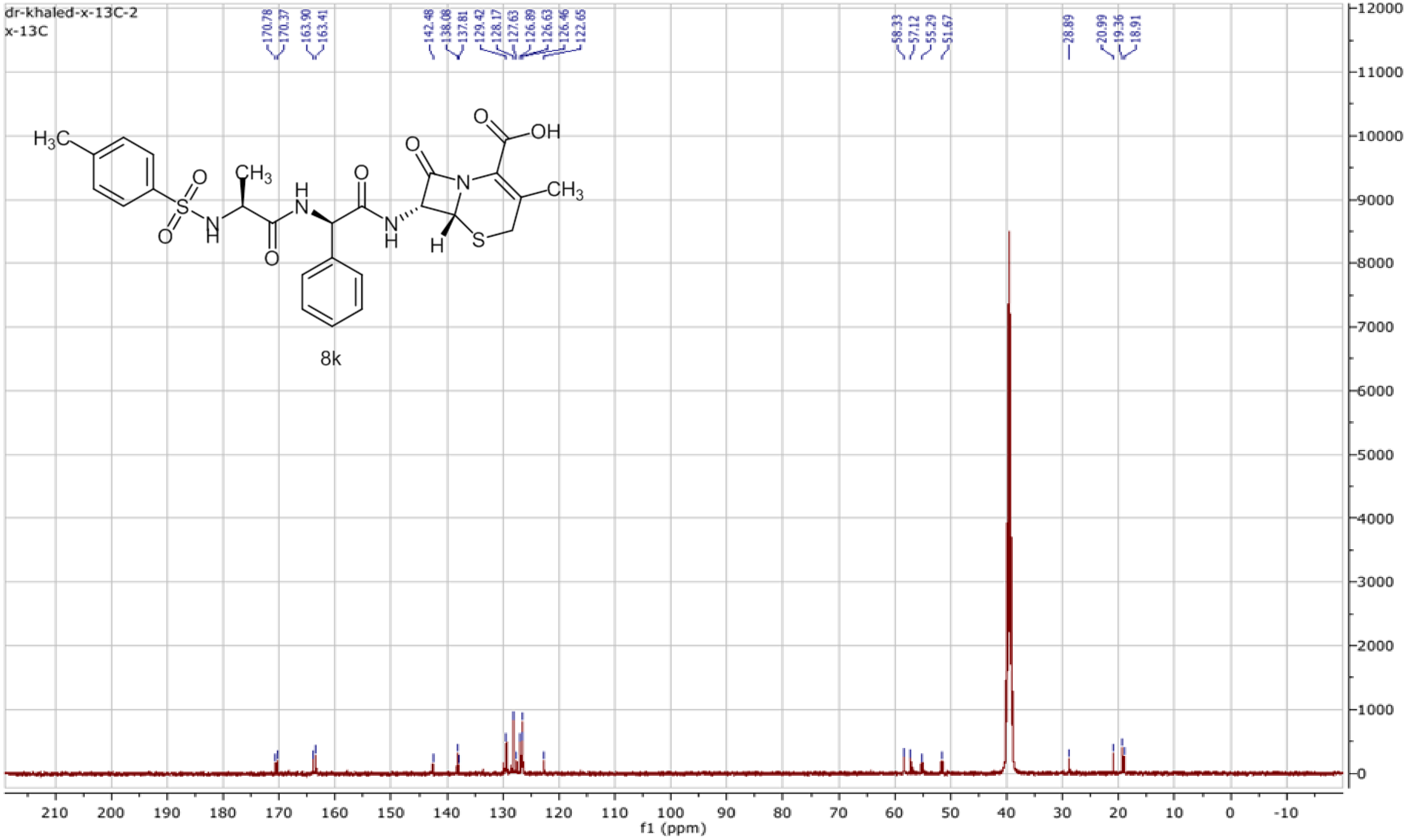
dr-khaled-x-13C-2
x-13C
170.78
170.37
163.90
163.41
142.48
138.08
137.81
129.42
128.17
127.63
126.89
126.63
126.46
122.65
58.33
57.12
55.29
51.67
28.89
20.99
19.36
18.91
8k
f1 (ppm)

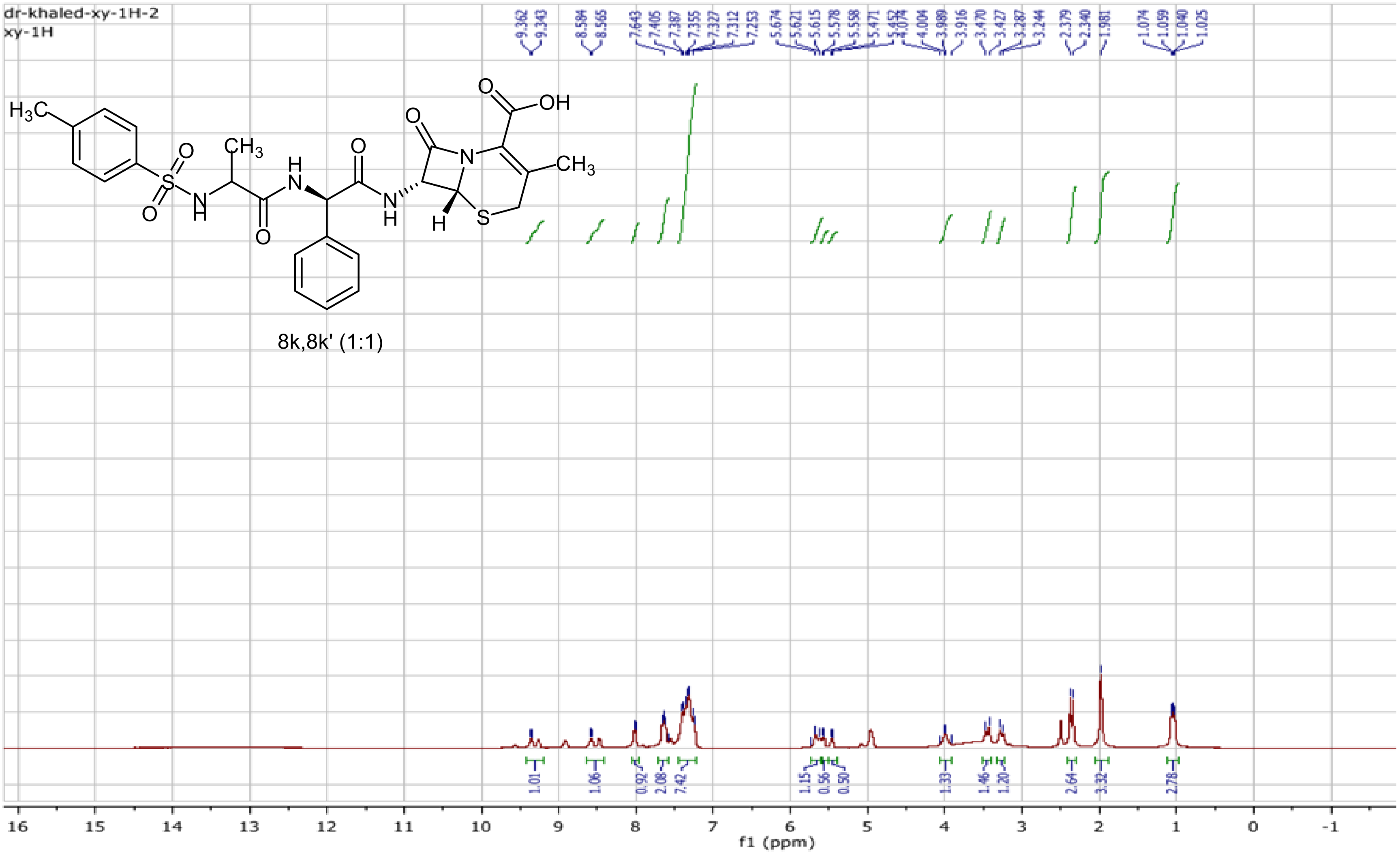
dr-khaled-xy-1H-2
xy-1H
8k,8k' (1:1)
f1 (ppm)

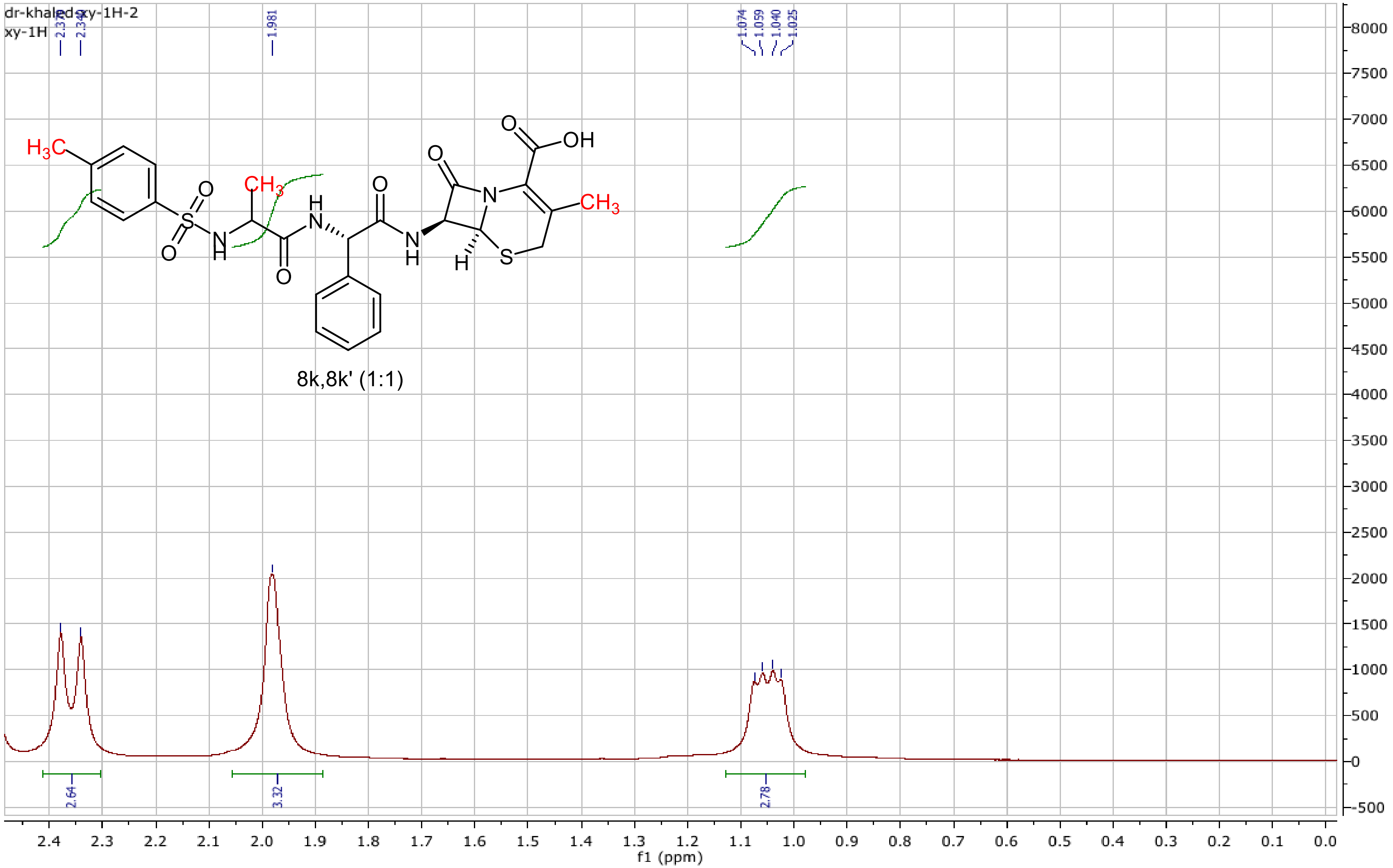
dr-khaled-xy-1H-2
xy-1H
2.370
2.340
1.981
1.074
1.059
1.040
1.025
8k,8k' (1:1)
2.64
3.32
2.78
f1 (ppm)

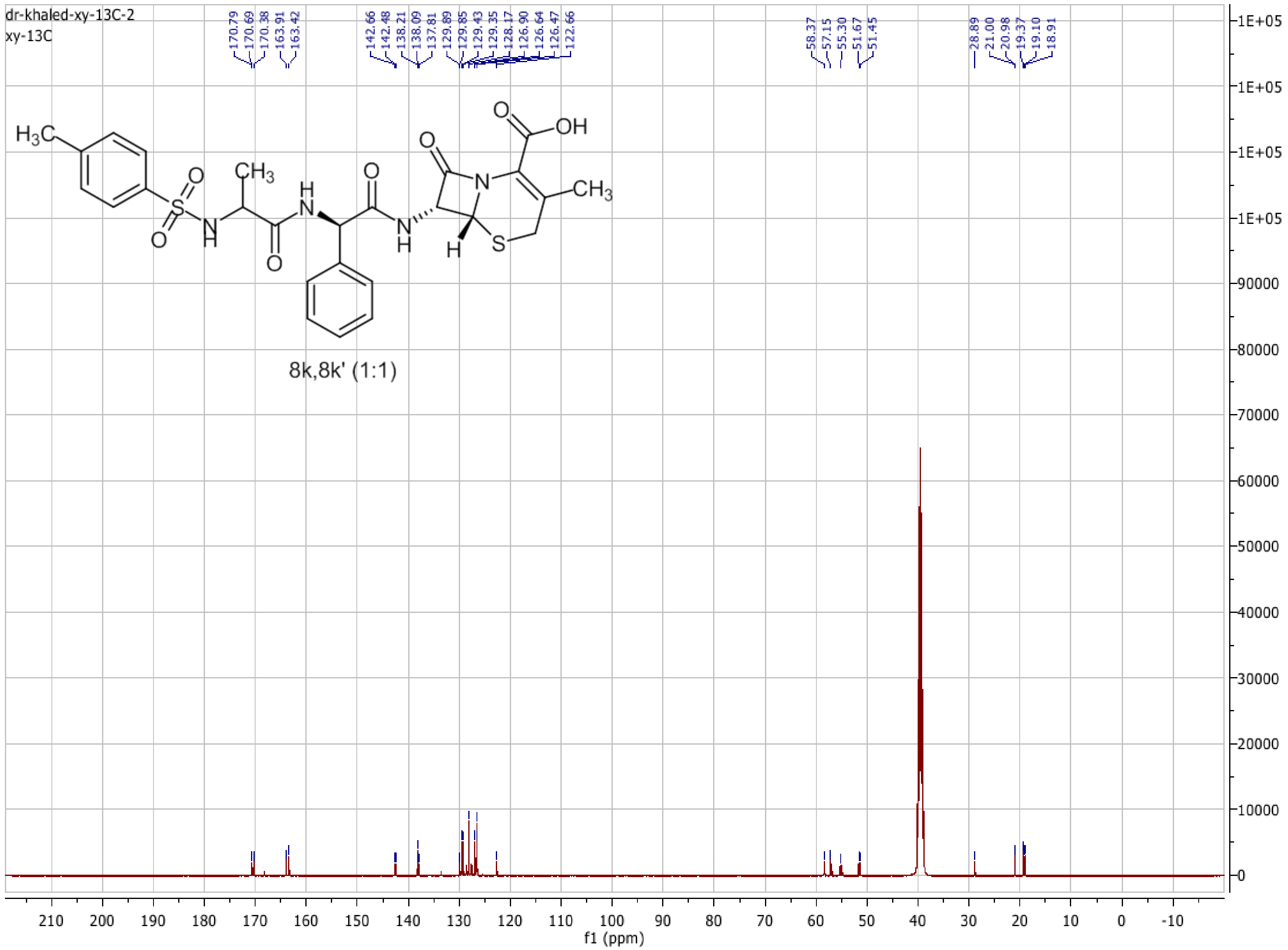
dr-khaled-xy-13C-2
xy-13C
170.79
170.69
170.38
163.91
163.42
142.66
142.48
138.21
138.09
137.81
129.89
129.85
129.43
129.35
128.17
126.90
126.64
126.47
122.66
58.37
57.15
55.30
51.67
51.45
28.89
21.00
20.98
19.37
19.10
18.91
8k,8k' (1:1)
f1 (ppm)
